# Foxs1-mediated transformation of CD34^+^ fibroblast to myCAFs promotes tumor growth

**DOI:** 10.1038/s44321-026-00476-8

**Published:** 2026-07-04

**Authors:** Junyao Yang, Ting Chen, Run Zhang, Jing Xu, Xinghe Zhao, Mei Yang, Zheyi Chen, Shasha Sun, Xiaocui Chen, Shuqiong Zhang, Liujun Jiang, Yingxia Zheng, Yanhua Hu, Qingbo Xu, Wei Lin, Lisong Shen, Li Zhang

**Affiliations:** 1 Department of Laboratory Medicine, Xin Hua Hospital, Shanghai Jiao Tong University School of Medicine, Shanghai, China; 2 Faculty of Medical Laboratory Science, College of Health Science and Technology, Shanghai Jiao Tong University School of Medicine, Shanghai, China; 3 Institute of Artificial Intelligence Medicine, Shanghai Academy of Experimental Medicine, Shanghai, China; 4 Department of Cardiology, The First Affiliated Hospital, School of Medicine, Zhejiang University, Hangzhou, China; 5 Department of Cardiology and Institute for Developmental and Regenerative Cardiovascular Medicine, Xinhua Hospital, School of Medicine, Shanghai Jiaotong University, Shanghai, China; 6 Department of Pediatric Orthopedics, Xin Hua Hospital, Shanghai Jiao Tong University School of Medicine, Shanghai, China; 7 State Key Laboratory for Oncogenes and Related Genes, Department of Cardiology, Shanghai Cancer Institute, Renji Hospital, School of Medicine, Shanghai Jiaotong University, Shanghai, China

**Keywords:** Cancer

## Abstract

Cancer-associated fibroblasts (CAFs) represent a major structural component of solid tumors and play crucial roles in cancer progression and drug resistance. However, their developmental origin, differentiation trajectory, and therapeutic potential remain poorly defined. Using advanced approaches—including inducible genetic lineage tracing, single-cell RNA sequencing, and spatial transcriptomic profiling—we identified a population of Cd34^+^Pi16^+^ fibroblast progenitors (Cd34^+^ CAFs) in both melanoma and gastric cancer. We delineated their differentiation trajectory toward Acta2^+^ CAFs, driven by the upregulation of the transcription factor Foxs1. This work establishes the developmental origin of Acta2^+^ CAFs and experimentally validates the *Cd34*^+^ to *Acta2*^+^ transition. By reverse-matching the transcriptional signatures of Acta2^+^ CAF differentiation with the CMap/LINCS L1000 drug perturbation database, we identified four small-molecule candidates predicted to inhibit tumor-induced *Foxs1* upregulation. These compounds effectively suppressed Cd34^+^ CAF differentiation, maintaining the progenitor-like Cd34^+^ state. Collectively, this study proposes a novel antitumor strategy that targets CAF lineage development to restrain tumor progression.

## Synopsis

A comprehensive atlas of CD34⁺ fibroblast progenitors within the tumor microenvironment was generated using single-cell sequencing and lineage tracing, delineating their differentiation trajectory toward myofibroblasts and identifying potential therapeutic targets to block this transition.

- In situ sequencing reveals that CD34⁺Pi16⁺ CAFs originate adjacent to blood vessels, suggesting a vascular adventitial progenitor origin.
- Dual-recombinase lineage tracing combined with diphtheria toxin ablation demonstrates that CD34⁺Pi16⁺ cells are an essential source of tumor myofibroblasts required for tumor growth.
- Foxs1 directly binds the α-SMA promoter to drive CD34⁺ to Acta2⁺ CAF conversion, and its knockdown suppresses tumor growth in vivo.
- Machine learning-based screening of CMap/LINCS L1000 identifies disulfiram and three other compounds that block CAF differentiation and restrain tumor progression.
- Pharmacological intervention preserves the CD34⁺Pi16⁺ progenitor state, with single-cell sequencing confirming that WH-4-023 reverses CAF phenotypic transition.

## Abstract (web summary)

A comprehensive atlas of CD34⁺ fibroblast progenitors within the tumor microenvironment was generated using single-cell sequencing and lineage tracing, delineating their differentiation trajectory toward myofibroblasts and identifying potential therapeutic targets to block this transition.

## Graphical abstract

**Figure:**
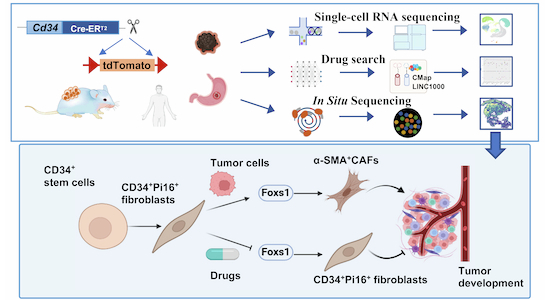

**Figure:**
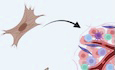

The paper explained **Problem**

Cancer-associated fibroblasts (CAFs) are a major stromal component and are linked to tumor growth, tissue remodeling, and treatment resistance. The clinical challenge is that CAFs are highly heterogeneous, so broad stromal targeting is often ineffective. A key biomedical question is which CAF states are most harmful, how these states arise during tumor progression, and whether their differentiation process can be interrupted therapeutically.

**Results**

This study used a discovery-to-validation pipeline centered on single-cell technologies. Single-cell RNA sequencing in melanoma and gastric cancer models identified an early progenitor-like fibroblast state and a later myofibroblastic, tumor-supporting CAF state. Similar populations were confirmed in human datasets and tumor tissues. Lineage-tracing experiments showed that early fibroblasts can transition into the pro-tumor CAF state over time, and selective depletion of this lineage reduced tumor burden in vivo. Spatial transcriptomic analysis added anatomical context, showing that early-lineage fibroblasts are enriched near vascular regions. To move from mechanism to intervention, the harmful differentiation signature was matched against CMap/LINCS perturbation databases, yielding repurposable compounds that were experimentally validated to suppress CAF-state transition features and inhibit tumor growth in preclinical models.

**Impact**

The main translational advance is the technical route itself: single-cell cell-state mapping, lineage and spatial validation, and signature-based computational drug discovery in one framework. This approach provides a practical strategy to discover tumor-microenvironment therapies that complement cancer-cell-directed treatment. More broadly, it supports a precision-oncology model that targets pathological cell-state transitions, not only static cell types.

## Introduction

The tumor microenvironment (TME) comprises a complex assembly of malignant, stromal, and immune cells that collectively influence tumor initiation, progression, and therapy response (Jimenez-Sanchez et al, 2017; Morrissy et al, 2017). While substantial research has focused on the phenotypic and functional diversity of tumor and immune cells, cancer-associated fibroblasts (CAFs) have emerged as central yet incompletely understood players in solid tumors (Yang et al, 2023). Despite growing attention and investment in CAF research (Chen and Song, 2019; Huang and Brekken, 2019; Huang et al, 2022), their origin and lineage relationships remain elusive.

Similar to epithelial tumor cells, CAFs exhibit extensive inter- and intratumoral heterogeneity. Beyond their structural role in the stromal matrix, CAFs possess diverse phenotypes and functions that critically shape tumor behavior. Ohlund et al first identified myofibroblastic CAFs (myCAFs) and inflammatory CAFs (iCAFs) in pancreatic cancer (Ohlund et al, 2017), revealing their distinct roles in modulating tumor growth and drug resistance (Elyada et al, 2019). Subsequently, antigen-presenting CAFs (apCAFs) were described in breast cancer (Bartoschek et al, 2018). α-SMA^+^ CAFs, corresponding to myCAFs, are known to promote tumor proliferation and progression. Understanding CAF heterogeneity and lineage dynamics is thus essential for uncovering novel therapeutic opportunities (McAndrews et al, 2022; Sun et al, 2022; Wu et al, 2021).

Single-cell RNA sequencing (scRNA-seq) has revolutionized the identification of cellular subpopulations, including CAF subsets, by enabling unsupervised clustering and high-resolution molecular profiling. However, the functional significance, lineage relationships, and therapeutic relevance of these subpopulations often require further experimental validation. In our scRNA-seq dataset from tumor tissues, we identified a distinct population of Cd34^+^ CAFs. Cd34 encodes a transmembrane glycoprotein expressed on early hematopoietic and endothelial progenitor cells and is widely recognized as a marker of hematopoietic stem/progenitor cells (Dong et al, 2023). We previously characterized *Cd34*^+^ cell differentiation into endothelial cells (Wang et al, 2022), and *Cd34*^+^ fibroblasts have been observed across multiple organs (Pu et al, 2022). These findings prompted us to explore the potential presence and functional significance of the Cd34^+^ lineage in tumors.

To trace the fate of these cells, we employed an inducible genetic lineage-tracing mouse model, allowing for temporal tracking of specific cell populations using canonical markers or fluorescent reporters (Bowling et al, 2020). This technique, widely applied to studies of cell differentiation, tissue development, and disease progression, provides crucial insight into the origin and dynamics of defined cell lineages (Chen et al, 2018).

In this study, we delineated the complete atlas of Cd34 lineage cells within the TME. Through integrative single-cell and spatial transcriptomic analyses of mouse and human tumors, we characterized *Cd34*^+^ fibroblast lineages, identified their differentiation trajectories, and uncovered their contribution to tumor biology. Furthermore, leveraging transcriptomic reversal matching and computational drug screening, we identified compounds capable of halting *Cd34*^+^ to *Acta2*^+^ CAF differentiation—offering a promising new avenue for antitumor therapy.

## Results

### Identification of *Cd34*^+^ fibroblasts in the melanoma and gastric cancer tumor microenvironment (TME)

The CAF population comprises phenotypically and functionally heterogeneous subtypes that are broadly distributed across cancers (Ansell and Vonderheide, 2013; Biffi and Tuveson, 2021). To comprehensively characterize CAF heterogeneity, we focused on melanoma and gastric cancer (GC)—representative tumors originating from ectodermal and endodermal lineages, respectively. Subcutaneous tumor models were established in mice using GFP-labeled B16 (melanoma) and MFC (GC) cells. Tumors were collected on days 10 and 20 post-inoculation, and non-tumor (GFP⁻) cells were isolated and profiled using the 10x Genomics Chromium platform to generate a single-cell atlas of the non-tumor microenvironment (Appendix Fig. S1). We obtained high-quality transcriptomes for 46,170 cells.

**Figure 1:**
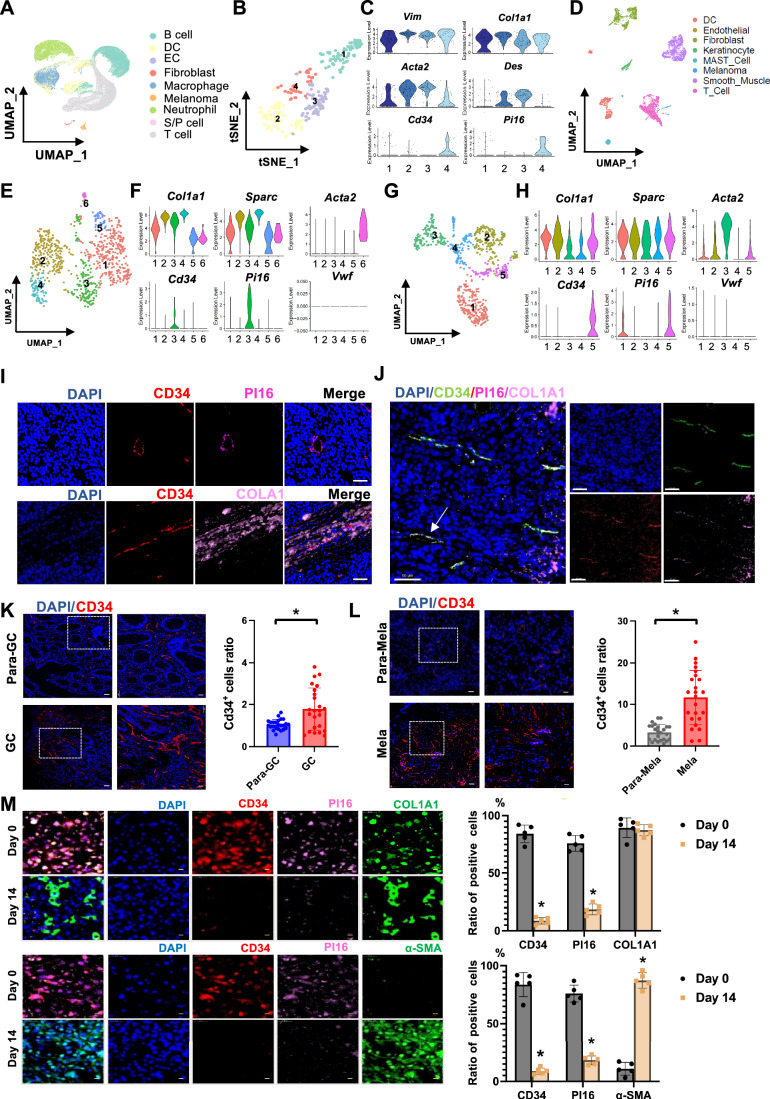
Characterization of *Cd34*^*+*^ fibroblasts in mouse subcutaneous and human gastric tumors. (**A**) UMAP plots showing major cell types in the mouse melanoma microenvironment, color-coded by cell identity. (**B**) UMAP plots of total fibroblasts from melanoma samples, color-coded by cluster. (**C**) Violin plots displaying normalized expression of *Cd34*, *Pi16*, *Acta2*, and *Des*. *n* = 6 (240 cells in total). Cluster 1: 77 cells; Cluster 2: 68 cells; Cluster 3: 59 cells; Cluster 4: 36 cells. (**D**) UMAP plot of major cell types in the human melanoma microenvironment. (**E**) UMAP plot of 793 melanoma fibroblasts from the fibroblast atlas. (**F**) Violin plots of *Col1a1*, *Sparc*, *Acta2*, *Cd34*, *Pi16*, and *Vwf* expression across fibroblast subclusters. *n *= 1 independent scRNA-seq experiment (793 cells in total).Cluster 1: 282 cells; Cluster 2: 227 cells; Cluster 3: 146 cells; Cluster 4: 79 cells; Cluster 5: 44 cells; Cluster 6: 15 cells. (**G**) UMAP plots of 725 fibroblasts from human gastric cancer samples, color-coded by cluster. (**H**) Violin plots of normalized expression levels of selected marker genes across fibroblast subclusters. *n* = 5 independent scRNA-seq experiments (725 cells in total).Cluster 1: 206 cells; Cluster 2: 173 cells; Cluster 3: 137 cells; Cluster 4: 114 cells; Cluster 5: 95 cells. (**I**, **J**) Immunofluorescence (IF) staining of *Cd34*, *Pi16*, and *Col1a1* in mouse melanoma (**I**) and human gastric cancer. (**J**) White arrows indicate representative CD34^+^PI16^+^COL1A1^+^ cells. (**K**, **L**) IF staining of *Cd34* in gastric cancer (**K**) and melanoma (**L**) tumor sections (*n* = 6), including paired para-cancerous and cancerous tissue, counterstained with DAPI (blue). Quantification of *Cd34*+ cells in gastric cancer and melanoma tumor sections. Data represent mean ± SD; *n* = 24. Statistical significance was assessed using a two-tailed Student’s *t* test; Significant exact *P* values were: (**K**) Para-GC vs. GC, *P* = 0.0010. (**L**) Para-Mela vs. Mela, *P* = 2.76 × 10^−7^. *P < 0.05. (**M**) IF staining for CD34, PI16, COL1A1, and α-SMA in isolated CD34*⁺* fibroblasts from human gastric cancer, with quantification of CD34^*+*^, PI16^*+*^, COL1A1^*+*^, and α-SMA^*+*^ cell fractions at day 0 and day 14 post-induction of differentiation. Data represent mean ± SD; *n* = 5 independent experiments. Statistical significance was assessed using an unpaired two-tailed Student’s *t* test. Significant exact *P* values were: top panel: CD34, Day 0 vs. Day 14, *P* = 3.03 × 10^−8^; PI16, Day 0 vs. Day 14, *P* = 2.86 × 10^−7^; bottom panel: CD34, Day 0 vs. Day 14, *P* = 2.97 × 10^−7^; PI16, Day 0 vs. Day 14, *P* = 2.62 × 10^−7^; α-SMA, Day 0 vs. Day 14, *P* = 4.56 × 10^−8^. *P < 0.05. Scale bars: white, 50 μm; yellow, 100 μm. Source data are available online for this figure.

Unsupervised clustering identified nine major cell types based on canonical markers: T cells (Cd3d, Cd3e), B cells (Cd79a/b, Cd19), dendritic cells (Cd86, Mzb1, Fgr), neutrophils (Cxcr2, S100a8, Fpr1), macrophages (Fcgr1, Adgre1, Itgam), fibroblasts (Sparc, Col3a1, Col1a1), endothelial cells (Pecam1, Vwf, Cldn5), melanoma cells (Tyr, Pax3, Mlana), and a distinct cluster expressing stem/progenitor-associated genes (*Cd34*, *Pcdh10*, *Stmn2*), which we defined as stem/progenitor-like (S/P) cells (Fig. 1A; Appendix Fig. S2A,B). Despite GFP− sorting, a small proportion of tumor cells—particularly melanoma—were retained. Fibroblasts were most abundant on day 20 in both GC and melanoma samples (Appendix Fig. S2C–E).

A total of 240 fibroblasts were captured and resolved into four subclusters (Fig. 1B). High expression of Col1a1, Vim, Col3a1, and Thy1 confirmed fibroblast identity. Subclusters 2 and 3 displayed classical myCAF features, including elevated Acta2 (encoding α-SMA) and Des (encoding desmin), whereas subcluster 4 exhibited strong Pi16 and Cd34 expression but minimal Acta2 (Fig. 1C; Appendix Fig. S2F). Hierarchical clustering based on Euclidean distances of principal component (PC) centroids revealed a close transcriptional relationship between Cd34^+^ S/P cells and fibroblasts (Appendix Fig. S2G), suggesting a lineage connection. Notably, S/P cells demonstrated elevated proliferative potential (Appendix Fig. S2H).

Given the limited fibroblast yield from our murine models, we validated the presence of *CD34*⁺ fibroblasts using two public single-cell RNA-seq datasets: GSE134388 (human melanoma) and GSE167297 (human GC). After uniform processing, we identified 793 fibroblasts from the melanoma dataset. Unsupervised UMAP clustering resolved eight major cell compartments (Fig. 1D), with corresponding marker profiles shown in Appendix Fig. S3A. Sub-clustering of CAFs revealed a distinct *CD34⁺PI16⁺ACTA2⁻* population (Fig. 1E,F). Applying the same analytical pipeline to the GC dataset yielded 725 fibroblasts and uncovered a transcriptionally similar *CD34⁺PI16⁺ACTA2⁻* population (Fig. 1G,H).

Across both murine and human tumor datasets, we consistently identified a discrete *CD34*⁺ fibroblast compartment. Immunostaining confirmed the presence of CD34^+^PI16^+^ fibroblasts in mouse melanoma (Fig. 1I) and human GC tissues (Fig. 1J). To exclude endothelial (*CD31*^*+*^) and pericyte (*RGS5*^+^) contamination, we further validated a *CD34*^*+*^*COL1A1*^*+*^*CD31*^*-*^*RGS5*^*-*^ population in human GC (Appendix Fig. S3B,C).

Interestingly, the CAF cluster showed the highest transcriptional similarity to S/P cells (Appendix Fig. S2G). Both compartments uniquely expressed *Cd34*, suggesting a potential lineage relationship—consistent with prior evidence that *Pi16*^+^ fibroblasts function as multipotent progenitors(Buechler et al, 2021; Pu et al, 2022). Furthermore, CD34 expression was markedly elevated in tumor tissue relative to adjacent normal mucosa in both human GC (Fig. 1K) and murine melanoma (Fig. 1L).

To assess lineage potential, we isolated *CD34*^+^ CAFs from human GC resections and cultured them for 14 days in differentiation medium supplemented with tumor-conditioned medium. Within 2 weeks, cells strongly upregulated α-SMA while downregulating *CD34* and *PI16*, whereas *COL1A1* expression remained stable (Fig. 1M).

Collectively, these findings identify *CD34*^+^ CAFs as a resident pro-fibroblastic pool within tumors and suggest that they serve as precursors to α-SMA^+^ myofibroblasts during cancer progression.

### *Cd34* lineage cells give rise to myCAFs

**Figure 2:**
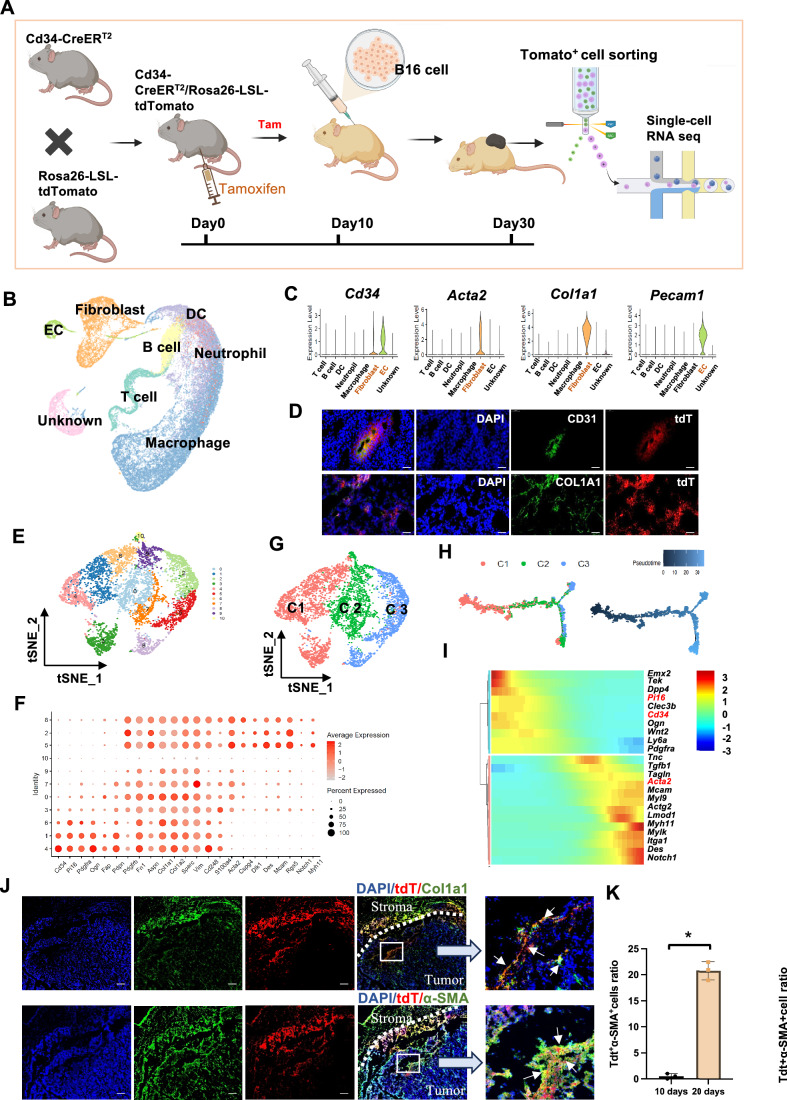
Lineage tracing demonstrates *Cd34*^*+*^ cells as a source of CAFs. (**A**) Schematic showing the generation of *Cd34*-CreERT2/Rosa26-LSL-tdTomato mice and bone marrow transplantation using *Cd34*-CreERT2;Rosa26-LSL-tdTomato mice as donors (top) or recipients (bottom). (**B**) UMAP plot of color-coded major cell types among Tomato^+^ cells derived from *Cd34*^+^ cells in the tumor microenvironment (TME) of *Cd34*-CreERT2;Rosa26-LSL-*tdTomato* mice (*n* = 33,424 cells). (**C**) Violin plots showing expression of *Cd34*, *Acta2*, *Col1a1*, and *Pecam1* across cell types. (**D**) IF staining for EC marker CD31 and fibroblast marker COL1A1 in melanoma tissues of *Cd34-CreERT2/Rosa26-LSL-tdTomato* mice. Scale bars: white, 50 μm. (**E**) t-SNE plots of color-coded fibroblast clusters from melanoma samples of *Cd34*-CreER^T2^/Rosa26-LSL-tdTomato mice. (**F**) Expression plots of selected representative genes in integrated fibroblast data (color key indicates log-normalized expression). (**G**) t-SNE plot showing fibroblast clusters CAF0 (*Cd34*^+^*Acta2*^*-*^) and CAF1 (*Cd34*^*-*^*Acta2*^+^). (**H**) Pseudotime analysis showing fibroblast subtype differentiation trajectories by cluster (left) and pseudotime (right). (**I**) Heatmap of differentially expressed genes ordered by pseudotime. (**J**) IF staining for tdTomato/COL1A1 and tdTomato/α-SMA in *Cd34*-CreERT2;Rosa26-LSL-tdTomato melanomas 20 days post subcutaneous tumor implantation. Dotted borders outline the tumor's invasive front/stromal regions. White arrows indicate representative tdTomato^+^α-SMA^+^ cells. Scale bars: white, 50 μm. (**K**) Quantification of tdTomato^+^α-SMA^+^ cells at 10 and 20 days post-implantation. Data represent mean ± SD of 6 views; *n* = 3 independent experiments. Statistical significance was assessed using an unpaired two-tailed Student’s *t* test; *P* = 4.26 × 10^−5^; *P < 0.05. Source data are available online for this figure.

To elucidate the origin of myCAFs within tumors, we employed a lineage-tracing mouse model. By crossbreeding Rosa26-LSL-Tdtomato and *Cd34*-(CreER^T2^) mice, we generated inducible lineage-tracing Cd34-*CreER; Rosa26-tdTomato* mice (Fig. 2A) (Jiang et al, 2021). The tandem dimer Tomato (tdTomato) fluorescent reporter specifically labels Cd34^*+*^ cells following tamoxifen induction and permanently marks their progeny, enabling longitudinal tracking as they differentiate into *Cd34*^−^ cell types (Song et al, 2023). Using fluorescence 3D tomography, we visualized tissue populations that had previously expressed *Cd34* after tamoxifen induction, identified by persistent tdTomato fluorescence (Appendix Fig. S4A).

*Cd34*-CreER; Rosa26-tdTomato mice received tamoxifen by gavage followed by subcutaneous melanoma implantation (Fig. 2A). To monitor the progression and differentiation of *Cd34*^*+*^ lineages within tumors, we isolated the tdTomato^*+*^ fraction (Appendix Fig. S4B) and subjected it to scRNA-seq (Appendix Fig. S4C). This approach enabled the construction of a comprehensive single-cell atlas encompassing 33,424 cells derived from three biological replicates.

Eight dominant cell types were identified and visualized in the *Cd34* lineage cell atlas (Fig. 2B), including T cells, B cells, neutrophils, dendritic cells, macrophages, fibroblasts, endothelial cells, and a small undetermined group likely representing false-positive tumor cells. The heatmap of enriched signature genes across these eight populations confirmed their identities (Appendix Fig. S4D). Among them, endothelial cells and fibroblasts were the only two retaining *Cd34* expression, whereas the others had differentiated into *Cd34*^*-*^ mature cell types (Fig. 2C,D). These findings suggest that *Cd34*^*+*^ cells directly contribute to the development of endothelial cells and fibroblasts.

From this dataset, 4594 fibroblasts were computationally isolated for detailed analysis. Unsupervised clustering at default resolution resolved these cells into 11 transcriptionally distinct subclusters (Fig. 2E). Based on their defining marker genes (Fig. 2F; Appendix Fig. S5A), we categorized them into three major fibroblast subpopulations (Fig. 2G). Within the melanoma dataset, neither apCAF nor iCAF signatures formed distinct clusters, indicating the absence of clearly defined counterparts (Appendix Fig. S5B). CAF1 (C1) was distinguished from CAF3 (C3) by elevated expression of *Cd34*, *Pi16*, and *Pdgfrα*, whereas C3 exhibited strong *Acta2* (α-SMA) expression. CAF2 (C2), positioned between these two, expressed *Pdpn* and *Pdgfrα* but showed only weak *Acta2* and *Cd34* expression (Appendix Fig. S5C).

The C1 signature (Cd34^high^Pi16^high^) indicates a population involved in progenitor-cell maintenance and expansion (Appendix Fig. S6). In contrast, C2 (*Tnc* ^high^*Cd34*^low^*Acta2*^low^) was enriched for genes encoding extracellular matrix (ECM) components associated with fibrotic remodeling, a hallmark of advanced tumors (Bonnans et al, 2014) and immune exclusion. C3 (*Acta2*^high^*Cd34*^low^) represented a contractile subset characterized by genes related to smooth muscle and actin cytoskeleton organization. Despite their transcriptional diversity, all three populations expressed fibroblast markers, confirming their CAF identity.

As these fibroblasts predominantly originated from *Cd34*-lineage cells undergoing phenotypic transitions, we performed pseudotime analysis to reconstruct their developmental continuum. The inferred trajectory positioned C1 at the earliest pseudotime (left), C3 at the latest stage (right), and C2 intermediately, consistent with progressive differentiation (Fig. 2H). A pseudotime-ordered heatmap revealed transcriptional shifts underlying this trajectory: *Cd34* and *Pi16* marked the early C1 state, while *Acta2* defined the mature C3 state (Fig. 2I). In the subcutaneous tumor tissue of CD34-labeled mice, immunofluorescence confirmed the presence of *Acta2+* cells at both the invasive front and within the stromal compartment (Fig. 2J). Quantification demonstrated that *tdT*^*+*^*α-SMA*^*+*^ cell numbers were significantly higher at 20 days post-implantation compared to 10 days (Fig. 2K).

### *Cd34*^*+*^*Pi16*^*+*^ lineage cells are essential to tumor growth as a source of myCAFs

We hypothesized that Cd34-lineage cells contribute to TME formation through the generation of structural and functional CAFs. To transcriptionally resolve *Cd34⁺* cells into progenitor and lineage-committed subsets, *Pi16* was employed as a discriminative marker. We generated *Cd34-Dre; Pi16-Cre* dual-recombinase lineage-tracing mice crossed with *Rosa26-tdTomato-DTR* reporter mice, yielding offspring capable of Cre/Dre-based lineage reporting and inducible cell ablation.

**Figure 3:**
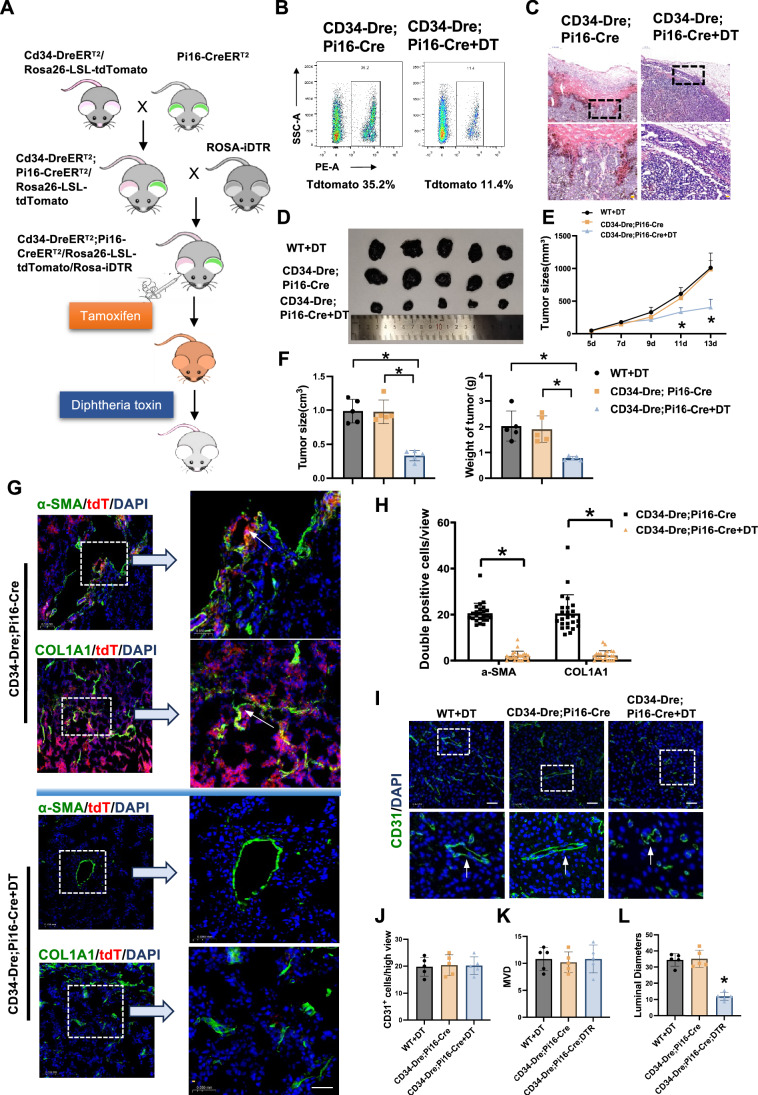
Depletion of *Cd34*^*+*^*Pi16*^*+*^ cells restricts tumor growth. (**A**) Schematic of *Cd34*-DreER^T2^/Rosa26; *Pi16*-CreER^T2^/Rosa26-LSL-tdTomato/ROSA-iDTR mouse generation. (**B**) Flow cytometry of peripheral blood from *Cd34*-DreER^T2^;*Pi16*-CreER^T2^/Rosa26-LSL-tdTomato/ROSA-iDTR mice with or without DT treatment. (**C**) H&E staining of tumor tissue from control and DT-treated mice 20 days after transplantation. Scale bars: white, 50 μm. (**D**) Photographs of excised tumors from WT and *Cd34*-Dre;*Pi16*-Cre mice with or without DT treatment. (**E**) Tumor volume over time. Data represent mean ± SD; *n* = 5. Statistical significance was assessed with one-way ANOVA followed by Bonferroni post hoc analysis at each time point; 11 d: WT + DT vs. CD34-Dre;Pi16-Cre+DT, *P* = 0.0005, and CD34-Dre;Pi16-Cre vs. CD34-Dre;Pi16-Cre+DT, *P* = 0.0044; 13 d:WT + DT vs. CD34-Dre;Pi16-Cre+DT, *P* = 0.0003, and CD34-Dre;Pi16-Cre vs. CD34-Dre;Pi16-Cre+DT, *P* = 0.0004;*P < 0.05. (**F**) Quantification of tumor volume (left) and tumor weight (right). Data represent mean ± SD; *n* = 5. Statistical significance was assessed using one-way ANOVA followed by Bonferroni post hoc analysis; Significant adjusted *P* values for tumor volume were: WT + DT vs. CD34-Dre;Pi16-Cre+DT, *P* = 4.47 × 10^−5^; CD34-Dre;Pi16-Cre vs. CD34-Dre;Pi16-Cre+DT, *P* = 5.27 × 10^−5^; Significant adjusted *P* values for tumor weight were: WT + DT vs. CD34-Dre;Pi16-Cre+DT, *P* = 0.0027; CD34-Dre;Pi16-Cre vs. CD34-Dre;Pi16-Cre+DT, *P* = 0.0055; *P < 0.05. (**G**, **H**) IF staining for α-SMA and COL1A1 in melanoma tissues from the two mouse groups (**G**), with quantification (**H**). White arrows indicate representative tdTomato⁺α-SMA⁺ or tdTomato⁺COL1A1⁺ double-positive cells. Scale bars: 50 μm. Data represent mean ± SD of six views; *n* represents the number of individual data points shown in the plot. Statistical significance was assessed using an unpaired two-tailed Student’s *t* test.α-SMA: CD34-Dre;Pi16-Cre vs. CD34-Dre;Pi16-Cre+DT, *P* = 4.97 × 10^−23^; COL1A1: CD34-Dre;Pi16-Cre vs. CD34-Dre;Pi16-Cre+DT, *P* = 5.49 × 10^−14^; *P < 0.05. (**I**, **J**) IF staining for CD31 in melanoma tissues from three mouse groups (**I**) and quantification of CD31^+^ cells (**J**). White arrows indicate representative CD31⁺ vascular structures. Scale bars: white, 50 μm. Data represent mean ± SD of six views; *n* = 5. Statistical significance was assessed using one-way ANOVA followed by Bonferroni post hoc analysis. (**K**) Microvessel density in melanoma tissues of the three groups. Data represent mean ± SD of six views; *n* = 5. Statistical significance was assessed using one-way ANOVA followed by Bonferroni post hoc analysis. (**L**) Luminal Diameters in melanoma tissues of the three groups. Data represent mean ± SD of six views; *n* = 5. Statistical significance was assessed using ordinary one-way ANOVA followed by Bonferroni’s post hoc analysis against the control group (WT + DT); WT + DT vs. CD34-Dre;Pi16-Cre+DT, *P* = 3.33 × 10^−6^; *P < 0.05. Source data are available online for this figure.

To test this hypothesis, we established a conditional *Cd34*^*+*^*Pi16*^*+*^ lineage-depletion model (*Cd34*-Dre; *Pi16*-Cre). Because C57BL/6 mice exhibit innate resistance to diphtheria toxin (DT), we crossed Cre-inducible diphtheria toxin receptor (iDTR) mice with *Cd34*-DreER^T2^;*Pi16*-CreER^T2^/Rosa26-tdTomato-DTR mice to generate *Cd34-Dre; Pi16-Cre-DTR* mice. This system enabled selective ablation of *Cd34*^+^*Pi16*^+^ lineage cells upon DT administration (Fig. 3A).

To synchronize lineage tracing and ablation, *Cd34-Dre;Pi16-Cre-DTR* and control *Cd34-Dre;Pi16-Cre* mice were first treated with tamoxifen to induce tdTomato expression, followed by a single intraperitoneal DT injection to eliminate *tdTomato*^+^*Cd34*^+^*Pi16*^+^ cells. This approach established a controlled temporal window for assessing the contribution of *Cd34*^+^*Pi16*^+^ lineages to melanoma progression. Flow cytometry confirmed ≥70% depletion of tdTomato^+^ cells within 48 h (Fig. 3B). Subcutaneous B16 melanoma cells were implanted in three groups: (1) *Cd34-Dre;Pi16-Cre-DTR* mice (ablation group), (2) *Cd34-Dre;Pi16-Cre* littermates (no-DTR control), and (3) wild-type C57BL/6J mice (additional control).

Histological analysis revealed substantially reduced tumor invasion in ablated mice (Fig. 3C), with tumors significantly smaller and lighter than those from controls (Fig. 3D,E). Tumor growth in *Cd34-Dre;Pi16-Cre* mice was comparable to wild-type controls, indicating that the genetic background alone did not affect tumor progression. Tumor size differences persisted throughout the observation period (Fig. 3F). Complete blood counts remained stable following DT treatment (Appendix Fig. S7), confirming selective targeting of the *Cd34*^+^*Pi16*^+^ compartment without systemic toxicity.

Using α-SMA and collagen immunofluorescence (IF) staining, we further characterized fibroblast development within tumors. Upon tamoxifen induction, *Cd34*^*+*^*Pi16*^*+*^ cells activated tdTomato, and their progeny retained permanent red fluorescence. Quantification of *α-*SMA^+^ tdTomato^*+*^ and COL1A1^*+*^tdTomato^*+*^ cells before and after DT treatment revealed significant reductions in both populations following ablation (Fig. 3G,H). To evaluate whether depletion of the *Cd34*^*+*^*Pi16*^*+*^ lineage affected tumor angiogenesis, we performed CD31 IF staining and quantified endothelial-cell abundance and microvessel density (Fig. 3I). Although total CD31^*+*^ cell counts (Fig. 3J) and overall vascular density (Fig. 3K) remained unchanged, vessels in *Cd34-Dre;Pi16-Cre-DTR* mice were smaller, exhibited narrower lumens, and displayed mild hyperbranching. These findings indicate that *Cd34*^*+*^*Pi16*^*+*^ cells are crucial for maintaining vascular integrity within the TME rather than promoting vessel proliferation.

### Foxs1 drives the differentiation of *Cd34*^*+*^*Pi16*^+^ CAFs into *Acta2*^+^CAFs

**Figure 4:**
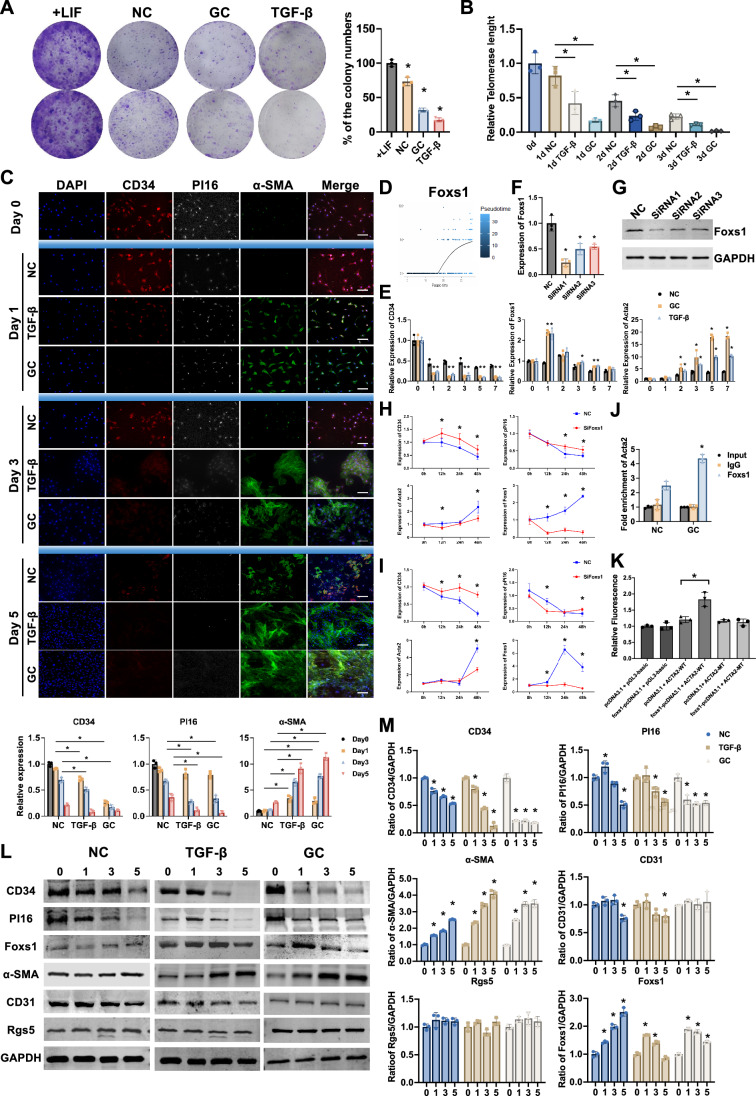
Foxs1 mediates differentiation of *Cd34*^*+*^*α-SMA*^*-*^ CAFs into *Cd34*^*-*^*α-SMA*^*+*^ CAFs. (**A**) Colony-formation assay of *Cd34*^*+*^*Pi16*^*+*^ CAFs exposed to stem cell medium containing LIF, differentiation medium, gastric cancer cell-conditioned medium, or TGF-β, compared to the LIF group. Data represent mean ± SD; *n* = 3 independent experiments. Statistical significance was assessed using an unpaired two-tailed Student’s *t* test against the +LIF group; +LIF vs. NC, *P* = 0.0043; +LIF vs. GC, *P* = 3.55 × 10^−5^; +LIF vs. TGF-β, *P* = 1.69 × 10^−5^; *P < 0.05. (**B**) Telomere length in CD34^+^ CAFs under negative control, gastric cancer cell-conditioned, or TGF-β-containing media at days 0, 1, 2, and 3. Data represent mean ± SD; *n* = 3 independent experiments. Statistical significance was assessed within each time point using an unpaired two-tailed Student’s *t* test against the NC group; 1 d: NC vs. TGF-β, *P* = 0.0343, and NC vs. GC, *P* = 0.0014; 2 d: NC vs. TGF-β, *P* = 0.0270, and NC vs. GC, *P* = 0.0018; 3 d: NC vs. TGF-β, *P* = 0.0069, and NC vs. GC, *P* = 0.0008; *P < 0.05. (**C**) IF staining for CD34, PI16, and α-SMA showing differentiation of sorted *Cd34*^+^*Pi16*^+^ CAFs cultured in control, gastric cancer cell-conditioned, or TGF-β media at days 0, 1, 3, and 5. Ratios of CD34^+^, PI16^+^, and α-SMA^+^ cells are compared to the negative control. Scale bars: 50 μm. Data represent mean ± SD of 6 views; *n* = 3 independent experiments. Statistical significance was assessed within each time point using an unpaired two-tailed Student’s *t* test against the NC group. CD34: Day 1: NC vs. TGF-β, *P* = 0.0007, and NC vs. GC, *P* = 1.07 × 10⁻⁵; Day 3: NC vs. TGF-β, *P* = 0.0103, and NC vs. GC, *P* = 0.0002.PI16: Day 3: NC vs. TGF-β, *P* = 1.72 × 10^−4^, and NC vs. GC, *P* = 0.0020; Day 5: NC vs. TGF-β, *P* = 0.0053, and NC vs. GC, *P* = 0.0026. α-SMA: Day 1: NC vs. TGF-β, *P* = 0.0020, and NC vs. GC, *P* = 0.0056; Day 3: NC vs. TGF-β, *P* = 1.22 × 10^−4^, and NC vs. GC, *P* = 1.16 × 10^−5^; Day 5: NC vs. TGF-β, *P* = 0.0003, and NC vs. GC, *P* = 1.93 × 10^−5^.*P < 0.05. (**D**) Pseudotime trajectory of genes involved in the transition process; dark blue indicates *Cd34*^+^*α-SMA*^*-*^ CAFs, light blue *Cd34⁻α-SMA*^+^ CAFs. (**E**) qPCR analysis of *Cd34*^,^ *Foxs1*, and *Acta2* in *Cd34*^*+*^*Pi16*^*+*^ CAFs cultured in control, gastric cancer cell-conditioned, or TGF-β media at days 0, 1, 2, 3, 5, and 7. Data represent mean ± SD; *n* = 3 independent experiments. Statistical significance was assessed using ordinary two-way ANOVA followed by Dunnett’s multiple comparisons test against the NC group at each time point. Significant adjusted *P* values were: *CD34*: Day 1: NC vs. GC, *P* = 6.03 × 10^−6^; NC vs. TGF-β, *P* = 0.0002. Day 2: NC vs. GC, *P* = 3.58 × 10^−9^; NC vs. TGF-β, *P* = 2.23 × 10^−7^. Day 3: NC vs. GC, *P* = 7.53 × 10^−9^; NC vs. TGF-β, *P* = 8.92 × 10^−8^. Day 5: NC vs. GC, *P* = 5.54 × 10^−5^; NC vs. TGF-β, *P* = 5.02 × 10^−5^. Day 7: NC vs. GC, *P* = 2.50 × 10^−6^; NC vs. TGF-β, *P* = 2.44 × 10^−6^. *Foxs1*: Day 1: NC vs. GC, *P* = 7.03 × 10^−18^; NC vs. TGF-β, *P* = 2.35 × 10^−17^. Day 3: NC vs. TGF-β, *P* = 0.0087. Day 5: NC vs. GC, *PP* = 0.0050. *Acta2*: Day 2: NC vs. GC, *P* = 0.0003; NC vs. TGF-β, *P* = 0.0352. Day 3: NC vs. GC, *P* = 6.24 × 10^−10^; NC vs. TGF-β, *P* = 9.25 × 10^−5^. Day 5: NC vs. GC, *P* = 6.93 × 10^−21^; NC vs. TGF-β, *P* = 1.60 × 10^−10^. Day 7: NC vs. GC, *P* = 5.45 × ^10–21^; NC vs. TGF-β, *P* = 8.33 × 10^−11^. *P < 0.05. (**F**, **G**) Effects of *Foxs1* siRNAs at mRNA (**F**) and protein (**G**) levels. (**F**) Data are presented as mean ± SD; *n* = 3 independent experiments. Statistical significance was assessed using predefined unpaired two-tailed Student’s *t* tests with Bonferroni correction against the NC group. Significant adjusted *P* values were: NC vs. siRNA1, *P* = 0.0042; NC vs. siRNA2, *P* = 0.0288; NC vs. siRNA3, *P* = 0.0231. *P < 0.05. (**H**, **I**) After *Foxs1* knockdown, *Cd34*^+^*α-SMA*^*-*^ CAFs were cultured in differentiation (**H**) or gastric cancer cell-conditioned (**I**) media. Expression of CD34, PI16, α-SMA, and *Foxs1* was measured by qPCR at 0, 12, 24, and 48 h. Data represent mean ± SD; *n* = 6. Statistical significance was assessed at each time point using *t* test comparing siFoxs1 with NC. Significant exact *P* values were: (**H**) *CD34*: 12 h, *P* = 0.0046; 24 h, *P* = 0.0029; 48 h, *P* = 0.0087. *PI16*: 24 h, *P* = 0.0020; 48 h, *P* = 0.0023. *Acta2*: 12 h, *P* = 0.0012; 48 h, *P* = 0.0016. *Foxs1*: 12 h, *P* = 4.27 × 10^−6^; 24 h, *P* = 4.87 × 10^−7^; 48 h, *P* = 7.59 × 10^−12^. (**I**) *CD34*: 12 h, *P* = 0.0141; 24 h, *P* = 0.0002; 48 h, *P* = 3.47 × 10^−7^. *PI16:* 12 h, *P* = 3.77 × 10^−5^; 48 h, *P* = 0.0063. *Acta2*: 48 h, *P* = 4.47 × 10^−7^. *Foxs1*: 12 h, *P* = 0.0003; 24 h, *P* = 6.46 × 10^−9^; 48 h, *P* = 2.81 × 10^−7^. *P < 0.05. (**J**) Chromatin immunoprecipitation (ChIP) assays of *Foxs1* binding in differentiation and gastric cancer cell-conditioned media. Data represent mean ± SD; *n* = 3 independent experiments. Statistical significance was assessed using an unpaired two-tailed Student’s *t* test; NC-Foxs1 vs. GC-Foxs1, *P* = 0.0015. *P < 0.05. (**K**) Dual-luciferase reporter assay assessing *Foxs1* binding activity at the *Acta2* promoter. Data represent mean ± SD; *n *= 3. Statistical significance was assessed using unpaired two-tailed Student’s *t* tests with Bonferroni correction for multiple comparisons; pcDNA3.1 + ACTA2-WT vs. Foxs1-pcDNA3.1 + ACTA2-WT, *P* = 0.0324; *P < 0.05. (**L**, **M**) Western blot time-course (**L**) of CD34, PI16, FOXs1, α-SMA, CD31, and RGS5 in sorted *Cd34*^+^*Pi16*^+^ CAFs cultured in control, gastric cancer cell-conditioned, or TGF-β media at days 0, 1, 3, and 5, with densitometric quantification (**M**) normalized to GAPDH and day 0. Data represent mean ± SD; *n* = 3 independent experiments. Data represent mean ± SD; *n* = 3 independent experiments. Statistical significance was assessed using ordinary two-way ANOVA followed by Dunnett’s multiple comparisons test against the corresponding 0 d group within each treatment condition. Significant adjusted *P* values were: CD34: NC, 0 d vs. 1 d, *P* = 4.81 × 10^−8^; 0 d vs. 3 d, *P* = 2.09 × 10^−11^; 0 d vs. 5 d, *P* = 1.22 × 10^−15^. TGF-β, 0 d vs. 1 d, *P* = 1.19 × 10^−6^; 0 d vs. 3 d, *P* < 1 × 10^−15^; 0 d vs. 5 d, *P* < 1 × 10^−19^. GC, 0 d vs. 1 d, *P* < 1 × 10^−18^; 0 d vs. 3 d, *P* < 1 × 10^−18^; 0 d vs. 5 d, *P* < 1 × 10^−19^. PI16: NC, 0 d vs. 1 d, *P* = 0.0058; 0 d vs. 5 d, *P* = 2.39 × 10^−8^. TGF-β, 0 d vs. 3 d, *P* = 0.00069; 0 d vs. 5 d, *P* = 2.48 × 10^−7^. GC, 0 d vs. 1 d, *P* = 1.08 × 10^−6^; 0 d vs. 3 d, *P* = 4.53 × 10^−8^; 0 d vs. 5 d, *P* = 5.10 × 10^−7^. α-SMA: NC, 0 d vs. 1 d, *P* = 6.63 × 10^−6^; 0 d vs. 3 d, *P* = 6.65 × 10^−9^; 0 d vs. 5 d, *P* = 1.55 × 10^−15^. TGF-β, 0 d vs. 1 d, *P* = 9.99 × 10^−15^; 0 d vs. 3 d, *P* < 1 × 10^−18^; 0 d vs. 5 d, *P* < 1 × 10^−21^. GC, 0 d vs. 1 d, *P* = 1.11 × 10^−16^; 0 d vs. 3 d, *P* < 1 × 10^−19^; 0 d vs. 5 d, *P* < 1 × 10^−19^. CD31: NC, 0 d vs. 5 d, *P* = 0.0087. TGF-β, 0 d vs. 5 d, *P* = 0.0282. Foxs1: NC, 0 d vs. 1 d, *P* = 6.09 × 10^−7^; 0 d vs. 3 d, *P* < 1 × 10^−14^; 0 d vs. 5 d, *P* < 1 × 10^−18^. TGF-β, 0 d vs. 1 d, *P* = 4.70 × 10^−11^; 0 d vs. 3 d, *P* = 1.27 × 10^−6^. GC, 0 d vs. 1 d, *P* = 1.51 × 10^−13^; 0 d vs. 3 d, *P* = 1.43 × 10^−13^; 0 d vs. 5 d, *P* = 2.87 × 10^−7^. *P < 0.05. Source data are available online for this figure.

As previously described, *Cd34*^*+*^*Pi16*^+^ fibroblasts within the tumor microenvironment can differentiate into α-SMA⁺ cancer-associated fibroblasts (CAFs). To further assess how this transition influences cellular stemness, we conducted colony-formation assays and telomere-length analyses. Compared with spontaneously differentiating cells, *Cd34*^*+*^*Pi16*^+^ fibroblasts exposed to tumor-cell-conditioned medium or TGF-β displayed markedly reduced clonogenic potential (Fig. 4A) and shortened telomeres (Fig. 4B). Stemness also progressively declined in the spontaneous differentiation group relative to cells maintained with LIF to prevent differentiation. Immunofluorescence (IF) staining on days 0, 1, 3, and 5 revealed that both tumor-conditioned medium and TGF-β downregulated CD34 and Pi16 while upregulating α-SMA, with the gastric cancer (GC) cell medium eliciting a stronger effect, likely due to additional pro-differentiation factors present in the medium (Fig. 4C).

To identify regulatory factors driving this differentiation trajectory, we performed pseudotime analysis and identified 9 transcription factors exhibiting concordant expression patterns. These candidates were subsequently validated by qPCR in CD34^+^ CAFs undergoing differentiation induction in vitro. Notably, Foxs1 upregulation displayed synchronized kinetics with Acta2, both temporally and quantitatively (Fig. 4D; Appendix Fig. S8), implicating *Foxs1* as a key regulatory node controlling this transition. Previous studies have shown that *Foxs1* directly *trans*-activates α-SMA expression (Bates et al, 2024). We therefore profiled the temporal mRNA expression of *Cd34*, *Acta-2*, and *Foxs1* over a 7-day differentiation course. Under control conditions, *Foxs1* mRNA increased on day 2, whereas cells treated with tumor-conditioned medium or TGF-β exhibited earlier induction by day 1. The rise in α-SMA expression consistently lagged behind *Foxs1* by ~24 h (Fig. 4E).

To test the causal requirement of *Foxs1*, three independent siRNAs were designed, and the most effective construct (si*Foxs1*-1) was selected for subsequent experiments (Fig. 4F,G). Upon *Foxs1* knockdown, cells cultured in either basal differentiation medium or tumor-conditioned medium exhibited partial restoration of CD34 and Pi16 expression and attenuated α-SMA induction, as shown by qRT-PCR (Fig. 4H,I). These results demonstrate that *Foxs1* is essential for efficient *Cd34*^+^ → α-SMA^+^ fibroblast conversion. Chromatin immunoprecipitation (ChIP) and dual-luciferase assays confirmed that *Foxs1* directly binds the α-SMA promoter and enhances its transcriptional activity (Fig. 4J,K). Together, these findings identify *Foxs1* as a decisive regulator in the transition from *CD34*^+^CAFs to *Acta2*^+^ CAFs.

We next tracked the expression of six key genes—*Cd34, Pi16*, Foxs1*, Acta2, Cd31, and Rgs5*—in *Cd34*^*+*^ fibroblasts at days 0, 1, 3, and 5 under three differentiation-inducing conditions. Both mRNA and protein levels followed consistent trends: CD34 and Pi16 decreased steadily, while FOXS1 and α-SMA increased (Fig. 4L,M). GC medium and TGF-β both accelerated this shift, though GC medium exerted the stronger effect. In summary, during *Cd34*^*+*^*Pi16*^*+*^ fibroblast differentiation, upregulated *Foxs1* activates α-SMA transcription, driving the emergence of α-SMA^*+*^ CAFs. Tumor-conditioned medium accelerates this process more effectively than TGF-β alone, likely due to its broader repertoire of pro-differentiation signals.

**Figure EV1:**
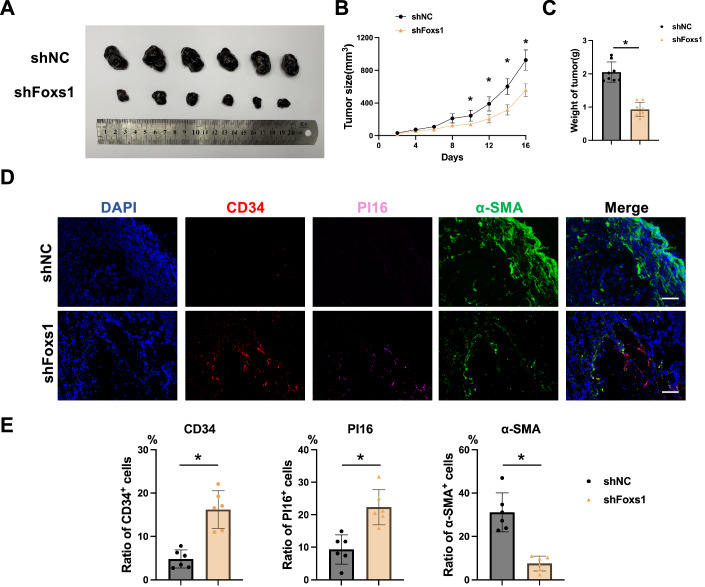
Foxs1 knockdown in fibroblasts inhibits tumor growth. (**A**) Photographs of excised tumors from shNC and shFoxs1 mice. (**B**) Tumor volume over time. Data represent mean ± SD; *n* = 8. Statistical significance was assessed at each time point using an unpaired two-tailed Student’s *t* test. Significant exact *P* values were: Day 10: shNC vs. shFoxs1, *P* = 0.0008; Day 12: shNC vs. shFoxs1, *P* = 1.49 × 10^−4^; Day 14: shNC vs. shFoxs1, *P* = 5.66 × 10^−6^; Day 16: shNC vs. shFoxs1, *P* = 5.13 × 10^−6^. *P < 0.05. (**C**) Quantification of tumor weight. Data represent mean ± SD; *n* = 8. Statistical significance was assessed using an unpaired two-tailed Student’s *t* test; shNC vs. shFoxs1, *P* = 6.82 × 10^−7^; *P < 0.05. (**D**, **E**) IF staining for CD34, PI16, and α-SMA/COL1A1 in tumor tissues from the two mouse groups (**D**), with quantification (**E**). Scale Bars:100 μm. Data are mean ± SD; *n* = 5. Statistical significance was assessed using an unpaired two-tailed Student’s *t* test; Significant exact *P* values were: CD34, shNC vs. shFoxs1, *P* = 0.0002; PI16, shNC vs. shFoxs1, *P* = 0.0011; α-SMA, shNC vs. shFoxs1, *P* = 0.0001; *P < 0.05.

To determine whether the transition of CD34^+^Pi16^+^ fibroblasts to myCAFs promotes tumor growth via foxs1 induction in vivo, we co-implanted tumor cells with CD34^+^ fibroblasts (3:1) expressing either foxs1-shRNA or control shRNA into recipient mice. Tumor growth was markedly suppressed in the shfoxs1 group, as evidenced by significant decreases in both tumor volume and weight (Fig. EV1A–C). Immunofluorescence staining showed that CD34 and Pi16 expression levels were substantially higher in the shfoxs1 group than in the control group, while α-SMA expression was concomitantly downregulated (Fig. EV1D,E). Collectively, these findings demonstrate that knockdown of foxs1 in CD34^+^ fibroblasts impedes their conversion to α-SMA^+^ CAFs and consequently attenuates tumor growth.

### Inhibitory molecules targeting the *Cd34*^+^ to *Acta2*^+^ CAF transition suppress tumor growth

Having established that the *Cd34*^*+*^ → α-SMA^+^ CAF transition promotes tumor progression, we next sought potential interventions capable of reversing the associated gene-expression changes. Using the CMap/LINCS L1000 and SignatureSearch databases, we implemented a machine-learning framework to identify small molecules that could counteract the gene expression signature driving this transition (Zhong et al, 2024). The model assigned weights reflecting each gene’s contribution to the state change; thus, drugs capable of reversing these transcriptional patterns were considered potential inhibitors of the pro-tumor CAF transition.

**Figure 5:**
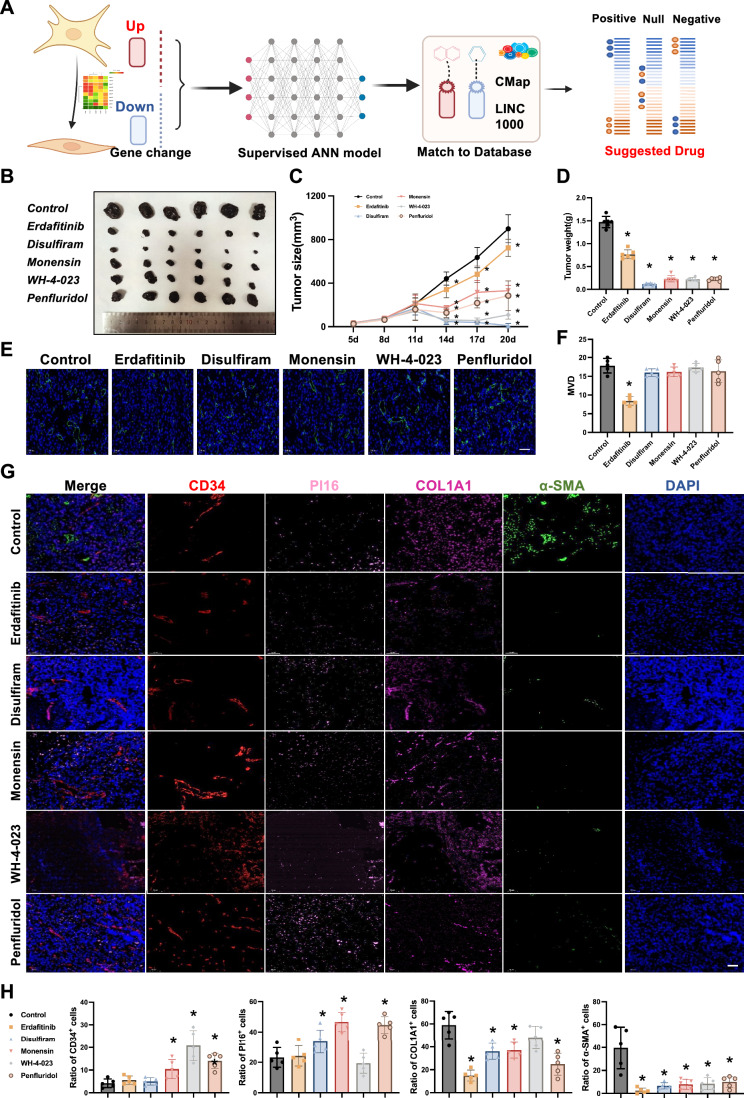
Drug screening identifies compounds that target CAF state transitions and suppress tumor growth. (**A**) Schematic of the drug identification pipeline based on transcriptomic signature matching between drug perturbations and CAF state transition profiles. (**B**) Tumor images from mice receiving different drug treatments. (**C**) Tumor growth curves under drug treatments. Data represent mean ± SD; *n* = 5. Statistical significance was assessed at each time point using ordinary one-way ANOVA followed by Bonferroni’s multiple comparisons test against the control group. Significant adjusted *P* values were: Day 14: Control vs. Erdafitinib, *P* = 0.009; Control vs. Disulfiram, *P* = 3.76 × 10^−12^; Control vs. Monensin, *P* = 1.08 × 10^−8^; Control vs. WH-4-023, *P* = 8.93 × 10^−12^; Control vs. Penfluridol, *P* = 4.38 × 10^−10^. Day 18: Control vs. Erdafitinib, *P* = 0.0091; Control vs. Disulfiram, *P* = 4.26 × 10^−12^; Control vs. Monensin, *P* = 7.50 × 10^−7^; Control vs. WH-4-023, *P* = 7.97 × 10^−12^; Control vs. Penfluridol, *P* = 6.18 × 10^−9^. Day 20: Control vs. Erdafitinib, *P* = 0.0208; Control vs. Disulfiram, *P* = 1.15 × 10^−13^; Control vs. Monensin, *P* = 1.37 × 10^−9^; Control vs. WH-4-023, *P* = 1.51 × 10^−12^; Control vs. Penfluridol, *P* = 2.96 × 10^−10^. *P < 0.05. (**D**) Tumor weight across groups. Data represent mean ± SD; *n* = 6. Statistical significance was assessed using one-way ANOVA followed by Bonferroni post hoc analysis; Control vs. Erdafitinib, *P* = 6.32 × 10^−16^; Control vs. Disulfiram, *P* = 5.74 × 10^−24^; Control vs. Monensin, *P* = 8.14 × 10^−23^; Control vs. WH-4-023, *P* = 6.20 × 10^−23^; Control vs. Penfluridol, *P* = 5.97 × 10^−23^; *P < 0.05. (**E**) IF staining for CD31 in melanoma tissues from drug-treated groups. (**F**) Quantification of microvessel density in melanoma tissues. Scale bars: white, 50 μm. Data represent mean ± SD of six views; *n* = 5. Statistical significance was assessed using ordinary one-way ANOVA followed by Bonferroni’s multiple comparisons test against the control group. Significant adjusted *P* value was: Control vs. Erdafitinib, *P* = 5.19 × 10^−8^; *P < 0.05. (**G**, **H**) IF staining for CD34, PI16, COL1A1, and α-SMA in tumor tissues (**G**) and corresponding quantification (**H**). Scale bars: white, 50 μm. Data represent mean ± SD of 6 views; *n* = 5. Statistical significance was assessed using an unpaired two-tailed Student’s *t* test against the control group. Significant exact *P* values were: CD34: Control vs. Monensin, *P* = 0.0164; Control vs. WH-4-023, *P* = 0.0007; Control vs. Penfluridol, *P* = 0.0005. PI16: Control vs. Disulfiram, *P* = 0.0456; Control vs. Monensin, *P* = 0.0004; Control vs. Penfluridol, *P* = 0.0005. COL1A1: Control vs. Erdafitinib, *P* = 5.84 × 10^−5^; Control vs. Disulfiram, *P* = 0.0058; Control vs. Monensin, *P* = 0.0072; Control vs. Penfluridol, *P* = 0.0010. α-SMA: Control vs. Erdafitinib, *P* = 0.0019; Control vs. Disulfiram, *P* = 0.0039; Control vs. Monensin, *P* = 0.0055; Control vs. WH-4-023, *P* = 0.0065; Control vs. Penfluridol, *P* = 0.0084. *P < 0.05. Source data are available online for this figure.

Because the Cd34^*+*^ → Acta2^+^ CAF shift is pro-tumorigenic, we screened the CMap and LINCS L1000 perturbagen libraries to identify candidate inhibitors (Fig. 5A). From the top 20 candidates in each database (Appendix Fig. S9A,B), we selected 40 compounds for validation and ultimately identified four drugs—disulfiram, monensin, WH-4-023, and penfluridol—that significantly suppressed tumor growth in both gastric cancer (Appendix Fig. S10A,B) and melanoma models (Fig. 5B–D; Appendix Fig. S10C). Erdafitinib, a tyrosine kinase inhibitor of FGFR1–4, was used as a positive control (Loriot et al, 2019). Among the candidates, disulfiram displayed the strongest tumor-suppressive efficacy.

To determine whether these antitumor effects were mediated through inhibition of angiogenesis, we performed CD31 staining and microvessel density (MVD) quantification on tumor sections. None of the four compounds altered intratumoral vessel density, whereas Erdafitinib significantly reduced angiogenesis (Fig. 5E,F).

IF staining of tumors from drug-treated mice confirmed that monensin, WH-4-023, and penfluridol maintained high CD34 expression, while disulfiram, monensin, and penfluridol preserved PI16 expression. All four drugs maintained CD34 expression while inhibiting α-SMA expression in vivo (Fig. 5G,H), aligning with in vitro findings that these treatments inhibit myCAF development.

**Figure EV2:**
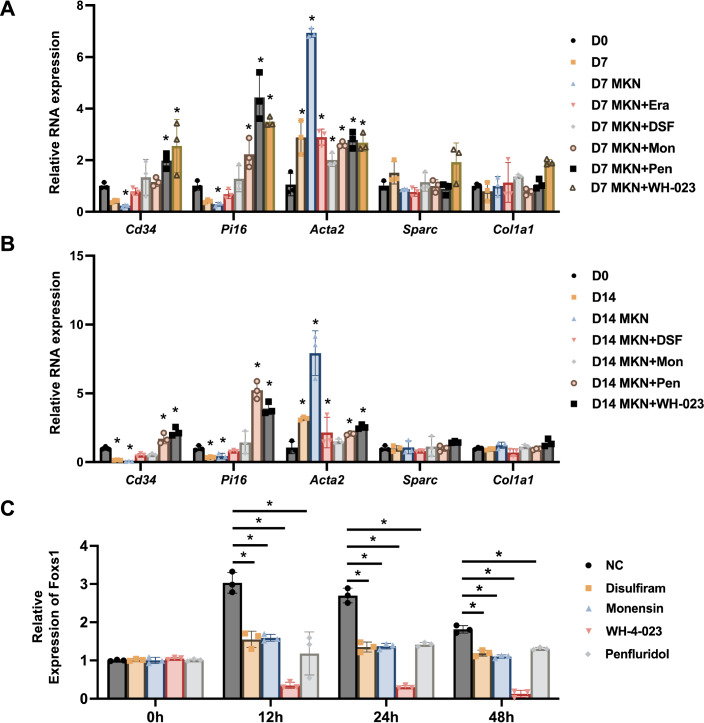
Drug candidates inhibit Foxs1 expression. (**A**, **B**) qPCR analysis of *Cd34*, *Pi16*, *Acta2*, *Sparc*, and *Col1a1* expression in *Cd34⁺PI16⁺* CAFs cultured under the indicated conditions for 7 days (**A**) or 14 days (**B**). Data represent mean ± SD; *n* = 3 independent experiments. Statistical significance was assessed using two-way ANOVA followed by Šídák’s multiple comparisons test comparing each condition with the D0 group within each gene. Significant Šídák-adjusted *P* values were: (**A**) *Cd34*: D7 MKN, *P* = 0.0011; D7 MKN+Pen, *P* = 0.0199; D7 MKN + WH-023, *P* = 0.0023. *Pi16*: D7 MKN, *P* = 0.0024; D7 MKN+Mon, *P* = 8.97 × 10^−5^; D7 MKN+Pen, *P* = 1.75 × 10^−9^; D7 MKN + WH-023, *P* = 7.84 × 10^−6^. *Acta2*: D7, *P* = 4.45 × 10^−6^; D7 MKN, *P* = 1.52 × 10^−18^; D7 MKN+Era, *P* = 4.65 × 10^−6^; D7 MKN + DSF, *P* = 0.015; D7 MKN+Mon, *P* = 3.25 × 10^−6^; D7 MKN+Pen, *P* = 6.35 × 10^−5^; D7 MKN + WH-023, *P* = 0.0014. (**B**) *Cd34*: D14, *P* = 6.43 × 10^−5^; D14 MKN, *P* = 0.0005; D14 MKN+Pen, *P* = 0.0434; D14 MKN + WH-023, *P* = 7.25 × 10^−5^. *Pi16*: D14, *P* = 0.0016; D14 MKN, *P* = 0.0213; D14 MKN+Pen, *P* = 7.41 × 10^−13^; D14 MKN + WH-023, *P* = 6.29 × 10^−11^. *Acta2*: D14, *P* = 3.53 × 10^−11^; D14 MKN, *P* = 0.0032; D14 MKN + DSF, *P* = 0.0081; D14 MKN+Pen, *P* = 0.003; D14 MKN + WH-023, *P* = 3.27 × 10^−6^. *P < 0.05. (**C**) qPCR profiling of Foxs1 in drug candidate-treated Cd34^+^Pi16^+^ cells for 0,12, 24, 48 h. Data are mean ± SD; *n* = 3 independent experiments. Statistical significance was assessed at each time point using an unpaired two-tailed Student’s *t* test against the NC group. Significant exact *P* values were: 12 h: NC vs. Disulfiram, *P* = 0.0018; NC vs. Monensin, *P* = 0.0010; NC vs. WH-4-023, *P* = 7.98 × 10^−5^; NC vs. Penfluridol, *P* = 0.0069. 24 h: NC vs. Disulfiram, *P* = 0.0006; NC vs. Monensin, *P* = 0.0004; NC vs. WH-4-023, *P* = 2.91 × 10^−5^; NC vs. Penfluridol, *P* = 0.0004. 48 h: NC vs. Disulfiram, *P* = 0.001; NC vs. Monensin, *P* = 0.0003; NC vs. WH-4-023, *P* = 2.80 × 10^−5^; NC vs. Penfluridol, *P* = 0.0011. *P < 0.05.

**Figure 6:**
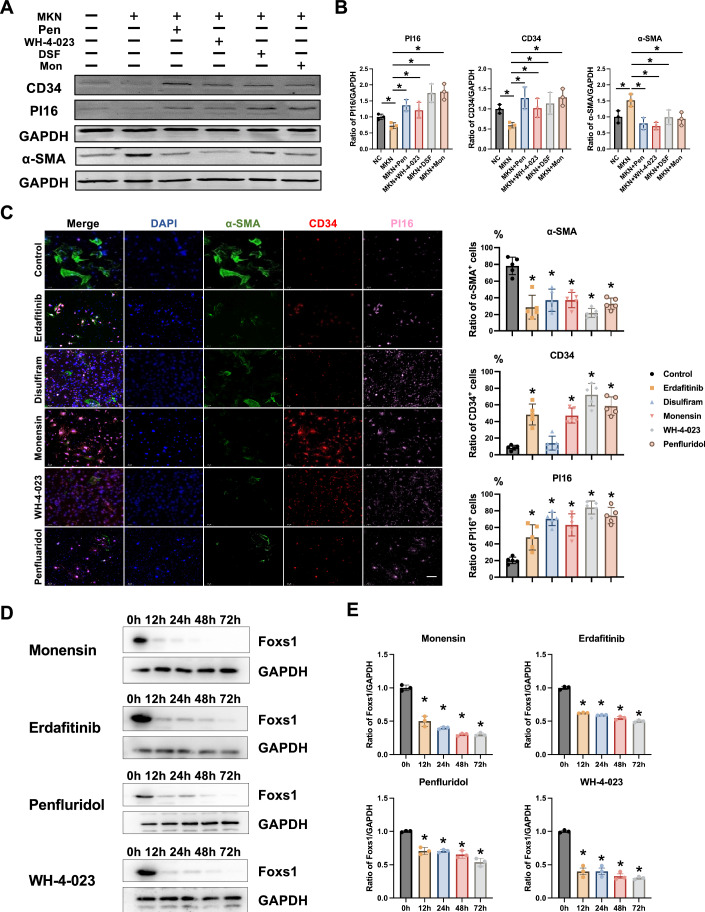
Drug candidates block the transition from *Cd34*^*+*^*α-SMA*^-^ to *Cd34*^*-*^*α-SMA*^+^ CAFs. (**A**, **B**) Protein expression of Cd34, Pi16, and α-SMA, of drug candidates treated in Cd34 + Pi16+ cells after being treated with MKN culture medium for 7 days (**A**), and each relative ratio compared with GAPDH (**B**). Data represent mean ± SD; *n* = 3 independent experiments. Statistical significance was assessed using an unpaired two-tailed Student’s *t* test against the MKN group. Significant exact *P* values were: PI16: NC vs. MKN, *P* = 0.0206; MKN vs. MKN+Pen, *P* = 0.0054; MKN vs. MKN + WH-4-023, *P* = 0.0338; MKN vs. MKN + DSF, *P* = 0.0041; MKN vs. MKN+Mon, *P* = 0.0022. CD34: NC vs. MKN, *P* = 0.0056; MKN vs. MKN+Pen, *P* = 0.0135; MKN vs. MKN + WH-4-023, *P* = 0.0401; MKN vs. MKN + DSF, *P* = 0.0278; MKN vs. MKN+Mon, *P* = 0.0056. α-SMA: NC vs. MKN, *P* = 0.0284; MKN vs. MKN+Pen, *P* = 0.0089; MKN vs. MKN + WH-4-023, *P* = 0.0032; MKN vs. MKN + DSF, *P* = 0.0352; MKN vs. MKN+Mon, *P* = 0.0232. *P < 0.05. (**C**) Representative immunostaining for CD34, PI16, and α-SMA in FACS-isolated CD34⁺ fibroblasts from human gastric cancer, with quantitative comparison of CD34⁺, PI16⁺, and α-SMA⁺ cell fractions across the different drug-treated groups. Scale bars: white, 50 μm. Data represent mean ± SD; *n* = 5 independent experiments. Statistical significance was assessed using ordinary one-way ANOVA followed by Bonferroni’s multiple comparisons test against the control group. Significant adjusted *P* values were: α-SMA: Control vs. Erdafitinib, *P* = 4.53 × 10^−7^; Control vs. Disulfiram, *P* = 9.25 × 10^−6^; Control vs. Monensin, *P* = 9.97 × 10^−6^; Control vs. WH-4-023, *P* = 4.60 × 10^−8^; Control vs. Penfluridol, *P* = 1.86 × 10^−6^. CD34: Control vs. Erdafitinib, *P* = 1.04 × 10^−5^; Control vs. Monensin, *P* = 1.64 × 10^−5^; Control vs. WH-4-023, *P* = 2.93 × 10^−9^; Control vs. Penfluridol, *P* = 2.51 × 10^−7^. PI16: Control vs. Erdafitinib, *P* = 0.0015; Control vs. Disulfiram, *P* = 3.99 × 10^−7^; Control vs. Monensin, *P* = 5.13 × 10^−6^; Control vs. WH-4-023, *P* = 4.63 × 10^−9^; Control vs. Penfluridol, *P* = 9.67 × 10^−8^. *P < 0.05. (**D**, **E**) Protein expression of Foxs1 in CAFs after treatment with MKN-conditioned medium (**D**), with quantification normalized to GAPDH (**E**). Data represent mean ± SD; *n* = 3. Statistical significance was assessed using an unpaired two-tailed Student’s *t* test against the 0 h group. Significant adjusted *P* values were: Monensin: 0 h vs. 12 h, *P* = 0.0006; 0 h vs. 24 h, *P* = 2.39 × 10^−5^; 0 h vs. 48 h, *P* = 1^.5^0 × 10^−5^; 0 h vs. 72 h, *P* = 1.64 × 10^−5^. Erdafitinib: 0 h vs. 12 h, *P* = 1.38 × 10^−5^; 0 h vs. 24 h, *P* = 9.39 × 10^−6^; 0 h vs. 48 h, *P* = 2.35 × 10^−5^; 0 h vs. 72 h, *P* = 8.78 × 10^−6^. Penfluridol: 0 h vs. 12 h, *P* = 0.0007; 0 h vs. 24 h, *P* = 3.37 × 10^−5^; 0 h vs. 48 h, *P* = 0.0004; 0 h vs. 72 h, *P* = 0.0002. WH-4-023: 0 h vs. 12 h, *P* = 0.0003; 0 h vs. 24 h, *P* = 0.0003; 0 h vs. 48 h, *P* = 4.69 × 10^−5^; 0 h vs. 72 h, *P* = 2.09 × 10^−6^. *P < 0.05. Source data are available online for this figure.

To test whether these compounds directly block CAF differentiation, we isolated *Cd34*^*+*^ CAFs from human gastric tumors, induced their conversion, and treated them with each candidate. Across all treatments, α-SMA mRNA and protein levels remained low (Figs. EV2A,B and 6A,B), *Cd34*^*+*^*Pi16*^*+*^ stem-like populations were preserved (Fig. 6C), and *Foxs1* expression was time-dependently repressed—most strongly by WH-4-023 (Figs. EV2C and 6D,E).

**Figure EV3:**
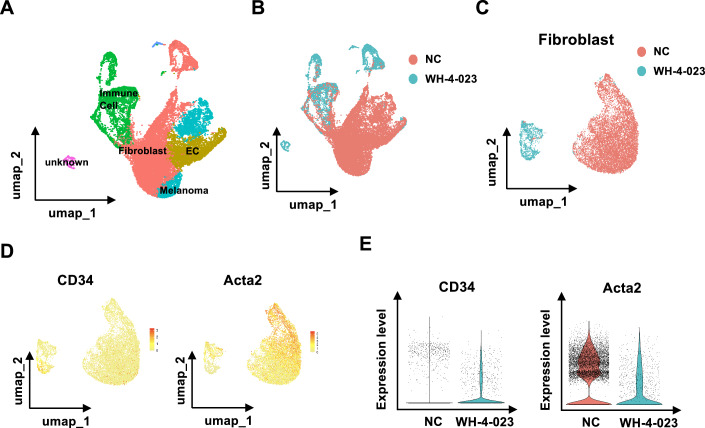
WH-4-023 inhibits the transition of CD34^+^ fibroblasts into Acta2+CAFs. (**A**) UMAP plot of the color-coded major cell types of Tomato^+^ cells encompassing *Cd34*^+^ cell-derived TME of *Cd34*-CreERT2; Rosa26-LSL-tdTomato mice. (**B**) UMAP plot of Tomato^+^ cells by samples. (**C**) UMAP plots of total fibroblasts of Cd34-CreERT2/Rosa26-LSL-tdTomato mice melanoma samples. (**D**) UMAP plots of CD34 and Acta2 of fibroblast in UMAP plots. (**E**) and Violin plots of CD34 and Acta2 expression by samples. Statistical significance was assessed using two-tailed Student’s *t* test. *n* = 6 (10,752 cells in total).NC: 9460 cells; WH-4-023: 1292 cells.

To further validate the effects of candidate drugs on CAF phenotypic transition in tumors, we selected WH-4-023 as a representative compound and performed single-cell RNA sequencing on subcutaneous melanomas from CD34 lineage-tracing mice following 1 week of treatment to examine phenotypic changes in CD34-derived cells upon drug administration. CD34-derived lineages were primarily classified into three major clusters: immune cells, fibroblasts, and endothelial cells (Fig. EV3A,B). Focusing on the fibroblast population (Fig. EV3C), we observed a marked phenotypic shift following treatment, characterized by a significant increase in the proportion of CD34+ fibroblasts and a concomitant decrease in Acta2^+^ CAFs. These results demonstrate that WH-4-023 effectively suppresses the conversion of CD34^+^ fibroblasts to Acta2^+^ CAFs in melanoma (Fig. EV3D,E). These results demonstrate that pharmacological inhibition of *Foxs1* represents a druggable mechanism to halt the *Cd34*^*+*^ → α-SMA^*+*^ CAF transition, maintaining fibroblasts in a less aggressive, tumor-suppressive state.

### In situ sequencing reveals initiation of *Cd34*-lineage CAFs near blood vessels

We next employed in situ sequencing (ISS) (Yu et al, 2021), a high-resolution spatial transcriptomics technique, to examine the spatial organization of CAF subsets within tumor tissues (Appendix Fig. S11A). Using a 16-gene panel, we mapped the spatial enrichment of signature genes within mouse melanoma tissue sections.

Hematoxylin–eosin (H&E) staining of frozen sections revealed distinct histological zones (Appendix Fig. S11B). Within central regions, we identified vascular structures exhibiting strong *Pi16* signals, while *Cd34* expression was more broadly distributed. *Pi16* specificity marks *Cd34*^*+*^*Pi16*^*+*^ CAFs, explaining the more localized Pi16 signal relative to the widespread *Cd34* expression. In the region highlighted by the red box (Appendix Fig. S11C,D), we observed colocalization of *Cd34*, *Pi16*, *Cdh5*, and *Acta2* near vascular structures. Nearest-distance analyses between *Cdh5* and *Pi16* further quantified their spatial proximity (Appendix Fig. S11E,F).

These spatial data suggest that Cd34^+^Pi16^+^ CAFs likely originate near blood vessels (Cdh5^*+*^), potentially from vascular adventitial progenitor cells residing in the vessel wall. However, whether these fibroblasts are locally derived or recruited from distant progenitor pools such as the bone marrow remains unclear. Resolving this question could fundamentally reshape our understanding of CAF ontogeny and the temporal coordination between CAF emergence and tumor vasculature development.

## Discussion

CAFs shape the structural framework of solid tumors, modulate the localization and activity of other cell types, and represent a fundamental component of the TME. The functional diversity of CAFs has led to the recognition that they comprise multiple subpopulations originating from distinct cellular sources (Biffi and Tuveson, 2021; Sahai et al, 2020). In this study, we identified and validated the presence of a Cd34^+^ CAF lineage in both mouse and human tumors, consistent with recent reports describing a stem-like phenotype in steady-state tissues (Buechler et al, 2021; Foster et al, 2022).

*The* Cd34^*+*^ lineage plays crucial roles in various physiological and pathological contexts, including tissue development, repair (Gaddam et al, 2019; Rodda et al, 2018), and angiogenesis (Liao et al, 2020). It is tightly associated with vasculature formation (Baron, 2001; Lavazza et al, 2010). Our findings expand this paradigm by revealing that Cd34^+^ fibroblasts also serve as a key origin of CAFs. We demonstrated that *Cd34*^+^ CAFs can differentiate into α-SMA^+^ CAFs through the upregulation of *Foxs1*, a transcription factor that directly binds the α-SMA promoter and is indispensable for its expression during differentiation. Notably, while *Cd34*^*+*^ CAFs are readily detected in mouse tumor models—likely due to their earlier developmental stage—they are rarely observed in mature human tumors, suggesting that this lineage may be transient or rapidly lost as tumors progress.

A particularly compelling aspect of this study is the discovery of a potential antitumor strategy based on inhibiting protumor CAF differentiation. By extending the CMap signature-matching approach to single-cell transcriptomic data, we prioritized repurposed drug candidates that suppress the transition from Cd34^+^ to Acta2^+^ phenotypes. These compounds effectively restrained tumor growth in both melanoma and gastric cancer models. Among them, disulfiram—an FDA-approved drug for alcohol dependence—showed the strongest efficacy. Monensin, a widely used antibiotic feed additive; penfluridol, an antipsychotic agent; and WH-4-023, a JAK inhibitor developed for autoimmune diseases, also exhibited notable tumor-suppressive activity. Although all four drugs have reported antitumor properties, their effects on CAF biology have not been described previously. Our study is the first to demonstrate that they can inhibit lineage progression from *Cd34*^+^ to α-SMA^+^ CAFs, introducing a new therapeutic avenue targeting myCAF formation. While these results highlight the promise of CAF-directed therapies, our current tumor suppression models remain simplified. Further work in more complex tumor microenvironments and eventual clinical studies will be required to translate these findings into viable cancer treatments.

ISS provides highly multiplexed RNA detection while preserving spatial context (Wang et al, 2018; Yu et al, 2021). We applied ISS to explore the spatial relationship between CAF subtypes and tumor histology, revealing a close spatial association between Cd34^+^ CAFs and vascular structures. Gene set enrichment analysis further showed that Cd34^+^ CAF markers were enriched in pathways related to vascular development and were localized around Cdh5^+^ vasculature. These findings suggest that Cd34^+^ CAFs may play a role in maintaining vascular stability and integrity during tumor invasion. We hypothesize that preserving their undifferentiated state could contribute to vascular normalization within tumors, although further experimental validation is needed.

In summary, this study fills a critical gap in understanding CAF ontogeny by identifying the *Cd34*^*+*^ lineage as a key source of α-SMA^*+*^ CAFs and by uncovering *Foxs1* as a pivotal regulator of this transition. Moreover, our drug-repurposing approach demonstrates the feasibility of identifying compounds that reverse deleterious cellular transitions in pathological tissues. Despite these advances, CAFs remain a heterogeneous population, and comprehensive characterization of their subtypes will be essential for developing precise and effective CAF-targeted cancer therapies.

## Methods

**Table:** Reagents and tools table

Reagent/resource	Reference or source	Identifier or catalog number
**Experimental models**		
B16-F10 cells	ATCC	CRL-6475
MKN-45	National Collection of Authenticated Cell Culture	SCSP-5473
C57BL/6 J	Shanghai Model Organisms	N/A
615	Shanghai Model Organisms	N/A
**Recombinant DNA**		
**Antibodies**		
Anti-collagen I	Abcam	ab138492
Anti-VE-cadherin	Abcam	ab205336
Anti-CD31	Abcam	ab24590
Anti-α-smooth muscle actin	Abcam	ab7817
Anti-C-kit	Abcam	ab283653
Anti-CD34	Abcam	ab81298
Anti-Pi16	R&D System	AF4929
Goat anti-rabbitHRP	Sigma	A0545
Goat anti-mouseHRP	Sigma	A0168
**Oligonucleotides and other sequence-based reagents**		
PCR primers	This study	
**Chemicals, enzymes, and other reagents**		
DMEM high glucose	ThermoFisher	41965062
Fetal bovine serum(FBS)	ThermoFisher	10270106
Phosphate-buffered saline (PBS)	ThermoFisher	10099141
Erdafitinib	MedChemExpress	HY-18708
Disulfiram	MedChemExpress	HY-B0240
Monensin	MedChemExpress	HY-N0150
WH-4-023	MedChemExpress	HY-12299
Penfluridol	MedChemExpress	HY-B1077
Chick embryo extract	Absin	abs80002
B27	Absin	Abs9120
Retinoic acid	Sigma	R2625
2-mercaptoethanol	Merck	M3148
P/S	Gibco^TM^	15140122
bFGF	ThermoFisher	13256-029
Leukemia inhibitory factor	Millipore	ESG1107
TGF-β	ThermoFisher	100-21-
6-well plate	Merck	CLS3506
Crystal violet	Merck	C0775
**Software**		
GraphPad Prism 9.0c	https://www.graphpad.com	
ImageJ	https://imagej.nih.gov/ij/index.html	
**Other**		

### Animals

C57BL/6J and 615 mice were obtained from Shanghai Model Organisms. Cd34-CreERT2, Cd34-DreERT2, Pi16-CreERT2, Rosa26-LSL-tdTomato, Rosa26-LSL-tdTomato-DTR, and Rosa-iDTR mice were maintained on a C57BL/6J background unless otherwise stated. Six-week-old male and female mice were used, with an approximately balanced sex ratio between groups when possible. Mice were housed under specific pathogen-free conditions in individually ventilated cages, with a 12 h light/dark cycle, controlled temperature and humidity, and free access to standard chow and water. All animal procedures were approved by the Ethics Committee of Xin Hua Hospital Affiliated to Shanghai Jiao Tong University School of Medicine under approval number XHEC-F-2020-026. All animal experiments were performed in compliance with institutional guidelines and relevant ethical regulations. The study design followed the principles of Replacement, Reduction, and Refinement.

### Mouse tumor models

Animals were divided into 3 groups: (1) control mice with diphtheria toxin injection, (2) *Cd34*-DreER^T2^;*Pi16*-CreER^T2^/Rosa26-LSL-tdTomato/Rosa-iDTR mice and (3) *Cd34*-DreER^T2^;*Pi16*-CreER^T2^/Rosa26-LSL-tdTomato/Rosa-iDTR mice with diphtheria toxin injection. Mice assigned to *Cd*34-Dre;*Pi16*-Cre and *Cd34*-Dre;*Pi16-*Cre with DT group received Tamoxifen at a dose of 0.15 mg/g/day by intragastric administration for five times, followed by 15 ng/g diphtheria toxin injection for four times. B16 cells were subcutaneously injected into the C57 mice. The mice were regularly examined until they were sacrificed. Tumor size was measured using a digital caliper, and tumor volume was calculated using the following formula: volume = 0.5 × width² × length. To avoid data variation incurred by sex differences, mice (male: female =  1:1) selected for the study were randomly allocated to different experimental groups. Tamoxifen (Sigma T5648) was dissolved in corn oil at a final concentration of 20 mg/ml, and 6-week-old mice were orally administered tamoxifen at 0.15 mg/g once every 3 days for a total of four times. Then, flow cytometry detection of Tdtomato expression in the peripheral blood of the mice was performed. Mouse tumor cell lines B16-F10-EGFP and mouse forestomach carcinoma (MFC-GFP) were obtained from the Chinese Academy of Sciences Cell Bank of Type Culture Collection and cultured according to their guidelines. Low passage cells were resuspended at 1 × 10^5^ cells/ml in a 1:1 mixture of PBS and Matrigel (Corning #356231) for B16F10 or MFC cells. One hundred microliters of cell stock were injected subcutaneously into the shaved right flank of C57BL/6 J mice (B16 F10) or 615 mice (MFC). For antitumor efficacy studies, at least 5 or more mice bearing ∼1000 mm^3^ tumors were weight-matched. The size of all the tumors is no more than 1000 mm^3^.

### CD34^+^PI16^+^ lineage tracing mouse model

Animals were divided into three groups: (1) control mice with diphtheria toxin injection, (2) Cd34-DreER^T2^;Pi16-CreER^T2^/Rosa26-LSL-tdTomato/Rosa-iDTR mice, and (3) Cd34-DreER^T2^; Pi16-CreER^T2^/Rosa26-LSL-tdTomato/Rosa-iDTR mice with diphtheria toxin injection. Mice assigned to CD34-Dre;PI16-Cre and CD34-Dre;PI16-Cre with DT group received Tamoxifen at a dose of 0.15 mg/g/day by intragastric administration for five times, followed by 15 ng/g diphtheria toxin injection for 4 times. B16 cells were subcutaneously injected into the C57 mice. The mice were regularly examined until they were sacrificed. Tumor size was measured using a digital caliper, and tumor volume was calculated using the following formula: volume = 0.5 × width² × length. To avoid data variation incurred by sex differences, mice (male: female = 1:1) selected for the study were randomly allocated to different experimental groups. Tamoxifen (Sigma T5648) was dissolved in corn oil at a final concentration of 20 mg/ml, and 6-week-old mice were orally administered tamoxifen at 0.15 mg/g once every 3 days for a total of four times. Then, flow cytometry detection of Tdtomato expression in the peripheral blood of the mice was performed. Mouse tumor cell lines B16-F10-EGFP and mouse forestomach carcinoma (MFC-GFP) were obtained from the Chinese Academy of Sciences Cell Bank of Type Culture Collection and cultured according to their guidelines. Low passage cells were resuspended at 1 × 10^5^ cells/ml in a 1:1 mixture of PBS and Matrigel (Corning #356231) for B16F10 or MFC cells. One hundred microliters of cell stock were injected subcutaneously into the shaved right flank of C57BL/6J mice (B16 F10) or 615 mice (MFC). For antitumor efficacy studies, at least five or more mice bearing ∼1000 mm^3^ tumors were weight-matched. The size of all the tumors is no more than 1000 mm^3^.

For the therapeutic model, 7–10 days after subcutaneous transplantation, drug candidates were injected directly into the tumor in situ every other day. The concentrations were as follows: Erdafitinib (positive control, 20 mg/kg), Disulfiram (20 mg/kg), Monensin (10 mg/kg), WH-4-023 (1 mg/kg), and penfluridol (10 mg/kg).

### Cell lines

B16-F10 cells were obtained from ATCC (CRL-6475). MKN-45 human gastric cancer cells were obtained from the National Collection of Authenticated Cell Culture (SCSP-5473). MFC-GFP cells were obtained from the Chinese Academy of Sciences Cell Bank of Type Culture Collection. Cells were cultured according to the suppliers’ recommendations. B16-F10-EGFP and MFC-GFP cells were used for mouse tumor implantation, and MKN-45-conditioned medium was used for in vitro differentiation assays where indicated. Cells were maintained in a humidified incubator at 37 °C with 5% CO₂. Cells were used at low passage and were routinely checked for morphology. Cell lines were authenticated by the suppliers and were tested for mycoplasma contamination before use; only mycoplasma-negative cells were used in the experiments.

### Human participants and tissue samples

Fresh gastric cancer tissues and paired adjacent non-tumor tissues were obtained from patients who underwent surgical resection at Xin Hua Hospital Affiliated to Shanghai Jiao Tong University School of Medicine. None of the patients had received chemotherapy, radiotherapy, or other antitumor treatment before tumor resection. Adjacent non-tumor tissues were collected at least 5 cm away from the tumor margin. Written informed consent was obtained from all participants before sample collection.

The study involving human participants was approved by the Ethics Committee of Xin Hua Hospital Affiliated to Shanghai Jiao Tong University School of Medicine under approval number XHEC-D-2020-193. All experiments involving human samples were conducted in accordance with the principles of the World Medical Association Declaration of Helsinki and the Department of Health and Human Services Belmont Report, as well as relevant institutional and national ethical regulations.

### Sample collection and cell sorting

Ten patients who were pathologically diagnosed with gastric cancer were enrolled in this study. None of the patients had been treated with chemotherapy, radiation, or any other antitumor medicines prior to tumor resection. Paired, freshly excised tumors and adjacent normal tissues were obtained immediately after surgical removal. The adjacent normal tissues were at least 5 cm from the tumor tissues. Clinical samples were collected from Xinhua Hospital, Shanghai Jiaotong University School of Medicine. Prior to participation, written informed consent was obtained from all subjects. All studies were performed in accordance with the Declaration of Helsinki. The study was approved by the Research Ethics Board of Xinhua Hospitals, Shanghai Jiao Tong University School of Medicine.

Mice were sacrificed with cervical dislocation. Tumor tissue was carefully peeled off from the skin and quickly cut into small pieces. All procedures involving animals in the study contained 8 mice (C57BL/6J, 615 and *Cd34*-CreER^T2^; Rosa26-LSL-tdTomato mice) per group, and the protocol was approved by the Institutional Committee of Shanghai Jiao Tong University School of Medicine for Animal Research. To obtain single cells, the pooled tumor was washed with PBS 3× and then subjected to enzyme digestion with 10 mL of 2 mg/mL collagenase I (Invitrogen; 17018–029) and 2 mg/mL dispase II (Sigma; D4693) in Hank balanced salt solution containing calcium and magnesium for 30 min. Digested cells were filtered with a 40 μm filter (Corning) and then centrifuged at 300×*g* for 5 min. Cells resuspended in PBS were stained with a LIVE/DEAD Fixable Near-IR (APC/Cy7 channel) Dead Cell Stain Kit (Invitrogen; L34975, 1:1000) for 15 min. GFP^−^/Cy7^−^ cells or Tdtomato^*+*^/ Cy7^−^ cells were sorted with a FACS Aria II Cell Sorter (BD Biosciences), and the postsort purity was routinely >95% for the sorted populations.

### Isolation and culture of primary fibroblasts

Fresh gastric cancer tissues were cut into pieces and washed with phosphate-buffered saline (PBS; ThermoFisher, USA) before being dissociated. The single-cell suspension was the same as previously described. Dissociated cells were pelleted by centrifugation at 1200 rpm for 5 min and resuspended in PBS. These cells were purified with a *Cd34* MicroBead Kit (Miltenyi Biotec, Bergisch Gladbach, Germany) and cultured in S/P cell culture medium. For the differentiation assay, *Cd34*^*+*^ fibroblasts were cultured in differentiation medium plus tumor cell culture medium (3:1). Drug candidates were added to the differentiation medium directly, and the medium was changed every other day. The concentrations were as follows: Erdafitinib (positive control, 0.01 µM), Disulfiram (0.5 nM), Monensin (0.01 µM), WH-4-023 (0.5 µM), and penfluridol (10 µM).

The differentiation medium consisted of the following: DMEM + 2% chick embryo extract, 1% FBS (10099141, Thermo Fisher Scientific, Waltham, MA, USA), 1% N2, 2% B27, 100 nM retinoic acid, 50 nM 2-mercaptoethanol (2-ME: M6250, Sigma-Aldrich, St. Louis, MO, USA), 1% P/S, and 20 ng/ml bFGF.

The S/P cell culture medium consisted of the following: DMEM + 2% chick embryo extract, 1% FBS, 1% N_2_, 2% B27, 100 nM retinoic acid, 50 nM 2-mercaptoethanol, 1% P/S, 20 ng/ml bFGF, and 0.01% leukemia inhibitory factor (10 ng/ml) (LIF: ESG1107, EMD Millipore, Burlington, MA, USA).

All cells were cultured in a humidified atmosphere containing 5% CO_2_ at 37 °C. The primary fibroblasts were used before passage 5.

### Colony-formation assays

For colony-formation assays, 1 × 10^5^ cells were cultured into a 12-well plate, treated with normal, 10 ng/ml TGF-β, or 30% GC culture differentiation medium for 5 days in triplicate. Cells with different treated conditions were digested, and 1000 cells were seeded into a six-well plate in triplicate. After about 2 weeks of culture, the survival colonies were fixed with methanol and stained with 0.1% crystal violet.

### Telomere length assay

In total, 1 × 10^5^ cells were cultured in a 12-well plate, treated with normal, 10 ng/ml TGF-β, or 30% GC culture differentiation medium for 1, 2, or 3 days. Genomic DNA was isolated from cells using the Genomic DNA Mini Extraction Kit (Beyotime, D0063). Then, detection was performed using the Human Telomere Length Dye Assay Kit (Beyotime, D8025S).

### RNA-Seq library preparation for 10X genomics single-cell 5’ sequencing

FACS-sorted GFP − /Cy7− cells or Tdtomato^*+*^/Cy7− cells were resuspended at a concentration of 1 × 10^6^ cells/ml in FACS buffer, and the viability was higher than 85%. Single-cell library preparation was carried out according to the manual of the Single-cell Reagent Kits. Briefly, cell suspensions were first loaded on the Single Cell A Chip for gel bead-in-emulsion (GEM) generation and barcoding, aiming for a recovery of 10,000 cells per sample. Next, reverse transcription, RT cleanup cDNA amplification, and quantification were performed to build a 3’ gene library. Finally, the libraries were sequenced on a NovaSeq 6000 system with a NovaSeq 6000 S4 Reagent Kit (300 cycles).

### Preprocessing of scRNA-seq data

The 10x Genomics Cell Ranger (3.0.1 version) pipeline was used to demultiplex raw files into FASTQ files, extract barcodes and UMI, filter, and map reads to the GRCh38 reference genome, and finally generate a digital expression matrix with UMI counts of genes versus cells per sample. This output was then imported into the Seurat (v3) R toolkit for quality control and downstream analysis. All functions were run under default parameters unless otherwise specified. Low-quality cells (< 400 genes/cell and >10% mitochondrial genes) were excluded. As a result, 10,1684 cells with a median of 1757 detected genes per cell were included in downstream analyses. To remove the batch effect, the datasets collected from different samples were integrated using Seurat v3 with default parameters. The Cellranger aggr command was used to combine sequencing data from multiple libraries with the mapped sequencing depth.

### Visualization and clustering of single-cell transcriptomics

To facilitate investigation and visualization of gene expression as well as their modular signals, the cells were projected onto an embedding space (UMAP) based on their transcriptional profiles. First, the UMI count data of six merged samples were normalized and scaled using the “NormalizeData” and “ScaleData” functions with default parameter settings in the Seurat R toolkit v3.2.3 (http://satijalab.org/seurat). A total of 2000 highly variable genes were selected to conduct a PCA dimension reduction to 50 components, which was implemented with the FindVariableFeatures function and RunPCA function. Finally, the resulting 2-D layout of single cells is based on the top 20 PCs via the uniform manifold approximation and projection (UMAP) dimensional reduction technique or t-distributed stochastic neighbor embedding (t-SNE) wrapped in the ‘RunUMAP’ and ‘RunTSNE’ functions of the Seurat package, respectively. Unsupervised clustering was performed by the function FindClusters of Seurat.

### Marker genes, cell cycle score, enrichment analysis, and reanalysis of subpopulations

Clusters or subpopulations were visualized using tSNE or UMAP embedding by different colors. Markers for a specific cluster against all other cells were defined by the function FindAllMarkers. To visualize the gene specificity, marker expressions were input to make a violin plot, feature plot, dot plot, and heatmap using the Seurat function with VlnPlot, FeaturePlot, DotPlot, and DoHeatmap, respectively. Cancer hallmark gene sets, Gene Ontology (GO), and Kyoto Encyclopedia of Genes and Genomes (KEGG) pathway analyses were performed using the marker genes of certain subpopulations identified by the FindMarkers or FindAllMarkers function with an average log(fold change) >0.25 using the enricher function of ClusterProfiler (an R package) and then visualized with dot plot of the ggplot2 package (another R package). The CellCycleScoring function of Seurat was used to determine the cell cycle stage based on the transcriptional profile.

Based on the clustering of the immune and nonimmune populations, the designated populations (columns) were extracted from the original matrix using the subset function, and a new Seurat object was generated. We were also able to run the same analytical pipeline on this new Seurat object.

### Cell type annotation

SuperCT (Xie et al, 2019) was utilized for initial annotation. See GitHub link as ‘https://github.com/weilin-genomics/rSuperCT’. We meticulously examined the SuperCT annotations across each unsupervised cluster. For any given unsupervised cluster, if SuperCT identifies multiple cell types, we selected the type with the highest number of calls. Therefore, the cell types were annotated by clusters rather than by single cells. The canonical markers of each cell type were validated on the unsupervised clusters.

### Trajectory analysis

Pseudotime trajectory analysis was performed using the R package monocle2 (version 2.9) with mostly default settings. The top 1000 variable genes were used to define the progress of cellular transition in Monocle 2. Then, we performed reduced dimensionality and cell ordering. “plot_cell_trajectory” and “plot_pseudotime_heatmap” functions were used to generate the trajectory plot and pseudotime heatmap.

### Similarity metrics of single-cell transcriptomes

The similarities across the multiple cell populations were calculated as follows. The top ten PCs based on the sparse digitalized expression matrix were calculated and extracted. For each cell population, we calculated the centroids of their top ten PCs. Then, we used the centroids of these subpopulations to calculate their distance/similarity. The distance/similarity diagonal matrix was used to make a clustered heatmap whose colors were based on the value of distance/similarity.

#### Algorithm implementation

The method employs a multi-step computational pipeline that leverages principal component analysis (PCA) and hierarchical clustering to quantify inter-cluster relationships:

1. Dimensionality Reduction: The algorithm extracts the first 20 principal components from the PCA reduction of the Seurat object, capturing the major sources of transcriptional variation while reducing computational noise.
2. Cluster Centroid Calculation: For each cell type cluster, we compute the mean expression profile in the PCA space by averaging the principal component scores across all cells within each cluster, generating representative centroids that summarize the transcriptional characteristics of each cell population.
3. Similarity Matrix Construction: Pairwise similarities between cluster centroids are calculated using cosine similarity, which measures the cosine of the angle between two vectors in the high-dimensional PCA space. This metric is particularly suitable for transcriptional data as it captures the direction of gene expression patterns rather than absolute expression levels.
4. Distance-based Clustering: The cosine similarity matrix is converted to a distance matrix (distance = 1 − similarity) and subjected to hierarchical clustering using the McQuitty method, which generates a dendrogram representing the hierarchical relationships between cell type clusters.
5. Visualization: The resulting distance matrix is visualized as a heatmap with hierarchical clustering dendrograms, where darker colors indicate higher similarity (lower distance) between clusters.

### Ranking drugs using the SuperFeat model parameters and SignatureSearch algorithm

SuperFeat is an artificial neural network (ANN) framework designed to train feature scoring models and evaluate the cellular states of specific cell lineages. See the GitHub link as ‘https://github.com/weilin-genomics/rSuperFeat’. It quantifies the extent of cellular state shifts that result in either beneficial or adverse effects. The framework accepts a digitized gene expression matrix for both training and scoring. For an in-depth understanding of SuperFeat, please refer to the paper by Zhong et al (Zhong et al, 2024). For specific cellular state transitions, such as the Cd34+ to Acta2+ shift, we’ve trained a SuperFeat model equipped with gene weights. The top weighted genes can be subsequently employed in the search for drug signatures.

We employed the qSig function from the R package signatureSearch (Duan et al, 2020) to execute connectivity ranking. This ranking was based on the perturbagen-vs.-cell gene expression signatures across the CMap or LINCS L1000 databases. By utilizing the printTopWeights function from the rSuperFeat package, we extracted the top 150 genes with both positive and negative weights. These genes were then fed into the signature search. The subsequent drug rankings were derived from either the positive or negative connectivity scores. Such scores indicate the potential of these drugs to either instigate or reverse specific cellular state or feature changes, respectively.

### Overview of the alteration of the pathway genes by drugs

Appendix Fig. S10D shows the values of genes in a pathway of interest. The curve was generated based on the kernel density estimation of the z score values of the total genes in the LINCS data repository (saved in ExperimentHub h5 format), which was calculated using the density function of R. The red lines show the specific z score values of the gene members in the designated pathway (gene set). The distribution of the red lines facilitates the display of the overall upregulation (> 0) or downregulation (< 0) of this pathway by the perturbagen.

### ISS assay

In situ sequencing was achieved by using an improved in situ sequencing (IISS) that exploits a novel probing and barcoding approach (Tang et al, 2023) for highly multiplexed in situ gene expression profiling compared with earlier ISS methods (Ke et al, 2013). IISS introduces paired-end sequencing and a 2-base combinatorial encoding strategy for barcode interrogation, which can improve not only signal intensity but also specificity for in situ sequencing. The label sequencing interrogation probe was conjugated to Cy3, Texas Red, Cy5, or Alexa Fluor 750 (Life Technologies). The detailed experimental procedures have been described previously (Ke et al, 2013; Tang et al, 2023). Briefly, OCT-embedded fresh frozen tissues with a thickness of 10 μm were first fixed in 4% (w/v) paraformaldehyde (Sigma) for 5 min and washed twice with diethyl pyrocarbonate (DEPC)-treated PBS, followed by dehydration in an ethanol series. After three washes with Wash Buffer (SEERNA Bioscience), sections were permeabilized with 0.1 M HCl at 37 °C for 5 min. The sections were then incubated in order with the following solutions: target probes tagged with a specific barcode for each RNA molecule in hybridization mix at 37 °C for 4 h, ligation mix at 37 °C for 30 min, splint primers in the circularization mix at 37 °C for 30 min, and amplification mix for rolling circle amplification (RCA) at 30 °C overnight. For sequencing, the sections were incubated in the ligation buffer of anchor primers, label sequencing interrogation probes, and ligase at 30 °C for 45 min, followed by mounting with SlowFade Gold Antifade Mountant (Thermo Fisher Scientific) medium containing 0.5 μg/mL DAPI (Sigma). Then, the images were acquired by using a Leica DM6B microscope, after which the probes for sequencing were stripped off with Stripping Buffer (SEERNA Bioscience). Then, the slides were washed three times with Wash Buffer for 5 min to prepare the sections for the next sequencing cycle. The same procedures were applied for each cycle until all cycles were achieved (four cycles at most) by repeating the sequence of ligation, imaging, and stripping off.

### Analysis of RNA ISS data

#### Image registration

To target more genes than the number of distinct fluorophores, we used multiple cycles of imaging. However, experimental conditions between imaging cycles, such as the movement of the microscope slide on the stage, can cause the field of view to shift. Correcting this error by registering images to a common reference image is important for accurate localization of RNA targets. The scale invariant feature transform (SIFT) algorithm was used to extract the matching feature points between the common reference image and the image to be registered, and the change matrix between the two images was calculated according to the relative position relationship between each set of matching feature points. Then, the image was corrected by an affine transformation to achieve image registration.

#### Finding signal points

The watershed algorithm based on the local extremum is used to identify and locate the signals in each cycle of images, and the quartile method is used to filter the detected signal points. The recognition logic of the signal point is that the gray value of its central area is much higher than that of its surrounding area. To measure the quality of signal points and remove false signal points, the difference between the average gray value of 4 * 4 pixel areas in the center of the signal point and the surrounding 10 * 10 pixels is defined as the base score (BS). Each point was used to calculate a BS value in four fluorescent channels. The channel with the highest BS value (BSmax, here the BS value of the other three channels were named BS2, BS3, BS4) was defined as the true base channel. The sequencing quality was further defined by the following formula:

$$Q=-10{log }_{10}^{\left(1-\frac{{BS}max }{{BS}\max +{BS}2+{BS}3+{BS}4}\right)}$$

The definition of the Q value is similar to that of sequencing quality in NGS sequencing.

#### Decoding signal points

Signals from different cycles at the same position were connected to form a barcode sequence. To address the points obtained by each cycle that may not overlap completely, the surrounding 6*6 pixel area of the points was searched to find the nearest point as an effective signal point. When no effective signal was detected in a certain position of a certain cycle, it was defined as N. Any barcode with a single base quality less than 5 and an average quality less than 10 was filtered out. Finally, a gene-by-space matrix was generated and used in downstream analyses.

#### Nearest-distance analysis of gene pairs on spatial transcriptomics

Appendix Fig. 11C was plotted based on the following algorithm. The Euclidean distances between the total detected molecules of gene Y (*Y* axis gene) and the nearest detected molecule of gene X (gene for each bar on *X* axis) were calculated and summarized with descriptive statistics. The mean value and the standard error of each gene pair were calculated to make bar plots. The bar plots can show the proximities of the gene pairs and thus reveal their possible colocalizations. The abovementioned algorithm was implemented by the cKDTree function of the scipy package in Python.

#### Signal density over the spatial transcriptomics map

The coordinates of the in situ signal of the barcoded transcripts were acquired from Bascalling.csv, which contains the coordinates of the detected genes. The 2D density maps were generated based on 200 × 200 bins using the geom_bin2d function of the ggplot2 package with the input of x/y coordinates of the genes. The density maps were superimposed on the H&E staining image, which allowed us to characterize the histological structure of the signal-enriched area (in the red box).

### PCR

Total RNA was extracted using an RNeasy Mini Kit (QIAGEN) according to the manufacturer’s instructions. The concentration and quality of the total RNA were assessed with a Nanodrop spectrophotometer (Thermo Fisher Scientific). cDNA synthesis was performed using Prime-Script RT master mix (TaKaRa). Primers were obtained from Shanghai Sangon. qRT-PCR analysis was performed in triplicate on a 7900 HT Real-Time PCR System (Applied Biosystems) using SYBR Premix Ex Taq (TaKaRa). The primer sequences used for PCR/qPCR are listed in Table 1.

**Table 1:** Primer sequences used for qRT-PCR.

Gene name	Upstream primers	Downstream primers
Cd34	CCTACCAATGAGTCTGTTGAGG	GAAGTAGTAGGCAGTATGCCAG
Pi16	CCAGTGCCCTCTTGGCTAC	ACCTCGGTCACCCTTGGA
α-SMA	ATAGGCACGTTGTGAGTCACACCA	CGACACTGCTGACAGAGGCACCA
Sparc	GTCCCACACTGAGCTGGC	AAGCACAGAGTCTGGGTGAGTG
Col1a1	ACATGTTCAGCTTTGTGGACC	TAGGCCATTGTGTATGCAGC
RGS5	GGGTTGCCTGTGAGAATTACA	TGAAGTGGTCAATGTTCACCTCT
Foxs1	TGTCTCTCAACGAGTGTTTCG	TGTGGTCTATCTTGACAGGGC
Ets1	GATAGTTGTGATCGCCTCACC	GTCCTCTGAGTCGAAGCTGTC
Hmgb2	CGGGGCAAAATGTCCTCGTA	CGGAAGAGTCCGGGTGTTT
Hopx	ATTCCACCACGCTGTGCCTCAT	AGTCTGTGACGGATCTGCACTC
Id1	TATACCGACGGGGAAACGGA	CTTCAGCGACACAAGATGCG
Meox2	TCAGAAGTCAACAGCAAACCCAG	TTCACCAGTTCCTTTTCCCGAG
Nr2f1	GCCTCAAAGCCATCGTGCTG	CCTCACGTACTCCTCCAGTG
Nr2f2	ACCGGGTGGTCGCTTTTATG	GGCCTTGAGGCAGCTATACTC
TBX2	CTGACAAGCACGGCTTCA	GACAAGCACGGCTTCACC
GAPDH	CCAGCCTCGTCCCGTAGACA	CTGGTCCTCAGTGTAGCCCAAGATG

Primer sequences used for qRT-PCR analysis in this study are listed. Primers were synthesized by Shanghai Sangon and used for SYBR Green-based qRT-PCR assays.

### Luciferase reporter assay

A dual-luciferase assay was performed as per standard protocols (Promega, Madison, USA). The luciferase gene was cloned downstream of the wild-type or mutant region of the Acta2, and co-transfected into HEK293 with the pcDNA3.1-foxs1 and control vector. After 48 h transfection, the cells were lysed and the firefly luciferase activity was measured using the Dual-Luciferase® Reporter Assay System (Promega) normalized to Renilla luciferase as the internal reference. All experiments were performed in duplicate, with data pooled from three independent experiments.

### Immunofluorescence staining

For frozen sections, samples were further fixed in 4% PFA for 2 to 3 h, followed by dehydration in 30% sucrose overnight. Samples were then soaked in optimum cutting temperature compound (OCT, Tissue-Tek) for 30 min at room temperature and embedded and frozen in OCT at −80 °C. For paraffin sectioning, samples were further fixed in 4% PFA for 24 h, followed by gradient dehydration using different concentrations of ethanol (50% to 100%) and xylol for transparency, and finally immersed and embedded in paraffin. Sections of 3 μm were collected. Frozen section slides were immersed in PBS to dissolve the OCT, and paraffin section slides were immersed in xylol and gradient ethanol to dissolve the paraffin. Slides of the subcutaneous melanoma from *Cd34*-CreER^T2^; Rosa26-LSL-tdTomato or gastric cancer tissue from patients were treated with 0.2% Triton X-100 in PBS for 15 min, which was followed by blocking with 5% donkey serum at room temperature. Slides were then incubated with primary antibody solutions overnight at 4 °C. After three washes with PBS, secondary antibodies were applied for 1 h at 37 °C, followed by three washes with PBS. The nuclei were then stained with DAPI. All slides were mounted with fluorescence-protecting mounting medium. Images were acquired on a Leica SP5 confocal microscope and analyzed by ImageJ software. The following antibodies were used: anti-collagen I (Abcam, ab138492, 1:200), anti-VE-cadherin (Abcam, ab205336, 1:200), anti-CD31 (Abcam, ab24590, 1:100), anti-α-smooth muscle actin (Abcam, ab7817, 1:200), anti-c-kit (Abcam, ab283653, 1:200), anti-CD34 (Abcam, ab81298, 1:200), and anti-PI16 (R&D Systems, AF4929, 1:100). The nuclei were counterstained with DAPI (4’,6-diamidino-2-phenylindole). Stained tissue was mounted on slides, and images were taken with a Leica SP5 confocal microscope.

### Chromatin immunoprecipitations (ChIP)-qPCR

For each ChIP assay, 5 × 10^6^ cells were harvested and followed the manufacturer’s protocol of Chromatin Immunoprecipitation (ChIP) Assay Kit (Beyotime, P2078). In brief, cells were incubated with 1% fresh paraformaldehyde at 37 °C for 10 min to crosslink the histone/transcription factor complexes with DNA, followed by 0.1% glycine incubation at room temperature for 5 min. The nuclei pellets were sonicated using a sonicator. After centrifugation, the chromatin was immunoprecipitated with FOXS1 antibody (1:50 dilution, Novus, NBP2-84032) overnight with gentle rotation. The protein/DNA complexes were immunoprecipitated by ChIP-grade protein G magnetic beads with rotation for 2 h at 4 °C, followed by one wash in low-salt buffer, one wash in high-salt buffer, one wash in LiCl Immune Complex Wash Buffer, and two washes in TE buffer. The protein-DNA complexes were eluted for 5 min twice and reversed with NaCl at 65 °C for 4 h. The DNA was purified and then amplified by quantitative real-time PCR with the following primer stargeted to the human ACTA2 promoter ( + 747/ + 754), forward primer: 5’-TCTGATTACTGAAAAAGACAACCAC-3’ and reverse primer: 5’-TCACTTCACGAGCTGGCAAT-3’; human ACTA2 promoter ( + 518/ + 525), forward primer: 5’-AAGCACTGGATGGAACTCTGC-3’ and reverse primer: 5’-CTCTTCCGTGAGAGAAAGCCC-3’; human ACTA2 promoter ( + 886/ + 893), forward primer: 5’-CAACACAGAGATCCGTGCCT-3’ and reverse primer: 5’-TCATAGGCATCGTGTAAAGCA-3’; human ACTA2 promoter ( + 1584/ + 1591), forward primer: 5’-TGACAGGGTGACTGGTGATCT-3’ and reverse primer: 5’-ACATTCCGCATGTTGCTGGC-3’; human ACTA2 promoter (-1093/-1100), forward primer: 5’-ACCTGAGCGCATGCATAGTT-3’ and reverse primer: 5’-TGAGCAACACAAAGAAACCACA-3’; human ACTA2 promoter ( + 1756/ + 1763), forward primer: 5’-ACATACTTCAGTTCCTGGTTTCA-3’ and reverse primer: 5’-AAGATGCTTCGAGTCACCCA-3’. The ChIP-qPCR data were reported as a percentage of input.

### Differentiation

In all, 1 × 10^6^ or 1 × 10^5^ cells were cultured into a 10-cm dish or a 12-well plate, treated with normal, 10 ng/ml TGF-β, or 30% GC culture differentiation medium for 1, 3, 5 days. Cells in 12-well plates or 10-cm dishes were collected to extract RNA or protein, respectively.

### Western blot

Cells were collected and lysed in RIPA buffer with a Protease inhibitor cocktail (Invitrogen) and eluted with SDS loading buffer. The proteins were resolved in SDS-PAGE electrophoresis and transferred to PVDF membrane (IPFL00010; Merck Millipore). The membranes were then incubated with primary antibodies and Anti-rabbit IgG (H + L; DyLight 800 Conjugate; 5151S; Cell Signaling Technology). GAPDH and β-actin were employed as endogenous controls, and densitometry analysis was conducted using ImageJ software (NIH). The following antibodies were used: anti-α-smooth muscle actin (Abcam, ab7817, 1:1000), anti-c-kit (Abcam, ab283653, 1:200), anti-CD34 (Abcam, ab81298, 1:1000f), and anti-PI16 (R&D Systems, AF4929, 1:1000). HRP-conjugated goat anti-rabbit IgG (Sigma, A0545, 1:5000) and HRP-conjugated goat anti-mouse IgG (Sigma, A0168, 1:5000) were used as secondary antibodies.

### General statistical analyses

Data are expressed as mean ± SD. Statistical analyses were performed using GraphPad Prism 8. The number of biological replicates, independent experiments, mice, or quantified fields of view is indicated in the corresponding figure legends. No statistical method was used to predetermine sample size. Sample sizes were selected based on standard practices in similar experimental systems and previous experience to ensure biological relevance and reproducibility.

For animal experiments, mice of the appropriate genotype, age, sex, and health status were included and randomly assigned to experimental groups where applicable. Mice were excluded only if tumor implantation failed, drug or diphtheria toxin treatment was not completed for reasons unrelated to the experimental design, or samples were technically unsuitable for downstream analysis. For human sample studies, participants were included if they had pathologically confirmed gastric cancer and available tumor and adjacent non-tumor tissues. Participants were excluded if they had received preoperative antitumor treatment, had insufficient tissue quality or quantity for downstream analysis, or withdrew consent. For cell-based assays, samples were excluded only in cases of contamination, failed transfection, failed staining, or other predefined technical failure. Investigators were blinded to group allocation during image acquisition and quantification where feasible. For analyses requiring manual quantification, fields of view were selected using the same criteria across groups. No data points were omitted unless a predefined technical failure occurred and was documented.

For comparisons between two groups, statistical significance was assessed using unpaired two-tailed Student’s *t* tests unless otherwise stated. For comparisons among multiple groups, ordinary one-way ANOVA followed by Bonferroni’s multiple comparisons test was used where appropriate. For experiments involving two independent variables, such as treatment and time, ordinary two-way ANOVA was used. For multiple row-wise comparisons, multiple unpaired two-tailed Student’s *t* tests followed by false discovery rate correction were used where indicated. For multiple-group comparisons with small sample sizes or when parametric assumptions were not appropriate, the Kruskal–Wallis test followed by Bonferroni-corrected post hoc analysis was used. The specific statistical test used for each experiment is stated in the corresponding figure legend. *P < 0.05 was considered statistically significant. Only significant comparisons are indicated unless otherwise stated.

## Supplementary information

Appendix

Peer Review File

Source data Fig. 1

Source data Fig. 2

Source data Fig. 3

Source data Fig. 4

Source data Fig. 5

Source data Fig. 6

Expanded View Figures

## Data Availability

The sequencing data have been uploaded to the China National GeneBank DataBase (https://db.cngb.org/) under accession number CNP0009130. The source data of this paper are collected in the following database record: biostudies:S-SCDT-10_1038-S44321-026-00476-8.
