# Supplementary material for: Foxs1-mediated transformation of CD34+ fibroblast to myCAFs promotes tumor growth
Source: EMBO Mol Med. 2026 Jul 4;18(8):3200–25. doi: 10.1038/s44321-026-00476-8 (PMC13469093; doi:10.1038/s44321-026-00476-8)
Supplement: Supplementary file 1 [file 44321_2026_476_MOESM1_ESM.pdf]

Appendix

Table of Contents

**Appendix Figure S1**	P1
**Appendix Figure S2**	P2
**Appendix Figure S3**	P3
**Appendix Figure S4**	P4
**Appendix Figure S5**	P5
**Appendix Figure S6**	P6
**Appendix Figure S7**	P7
**Appendix Figure S8**	P8
**Appendix Figure S9**	P9
**Appendix Figure S10**	P10
**Appendix Figure S11**	P11

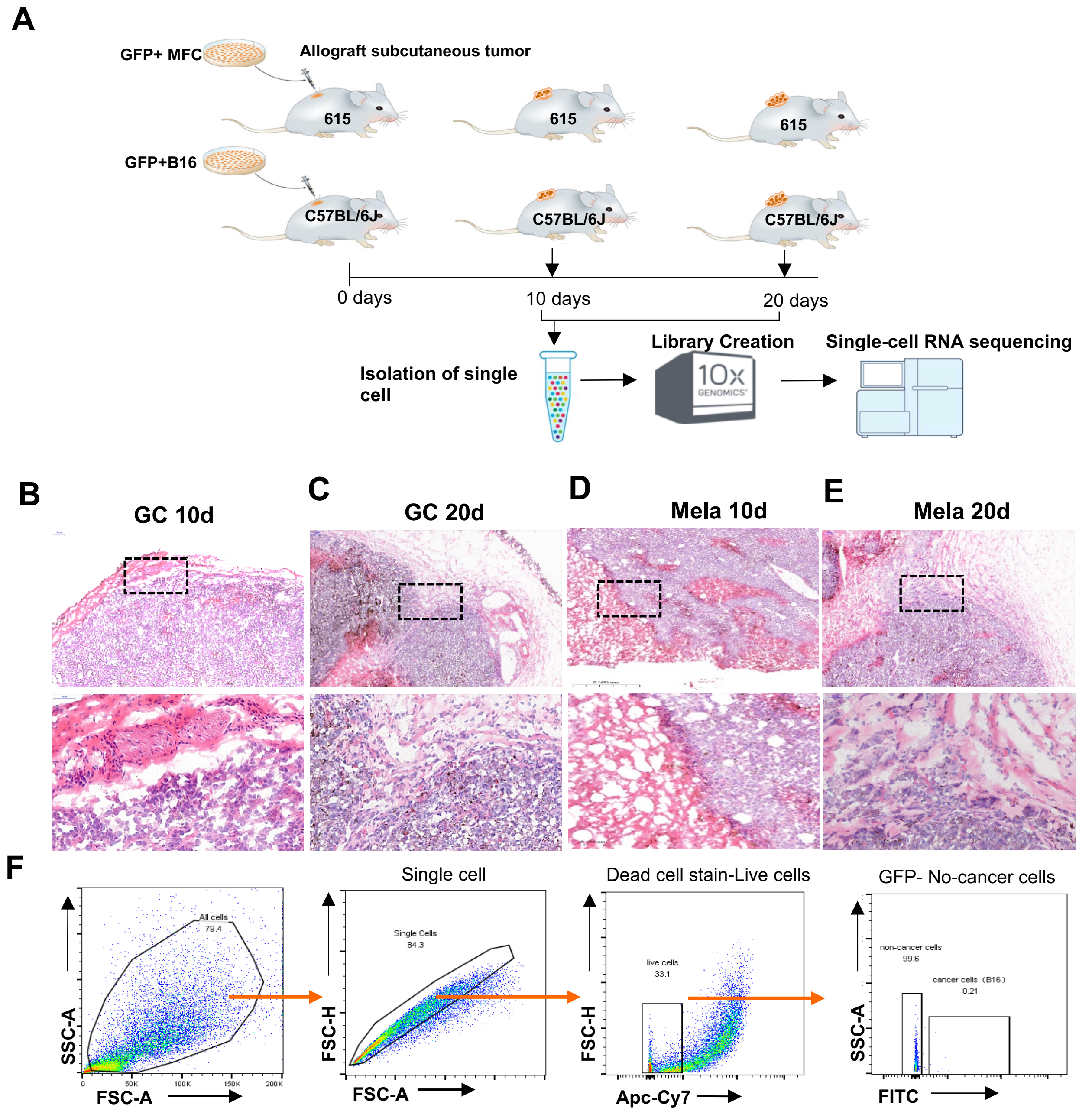

G

Parameter	GC10d	GC20d	Mela10d	Mela20d
Estimated Number of Cells	18,078	20,958	7,940	17,467
Fraction Reads in Cells	83.9%	90.6%	79.6%	88.6%
Mean Reads per Cell	16,646	17,029	43,903	14,687
Fraction of Reads kept after aggregation	100.0%	100.0%	100.0%	100.0%
Median Genes per Cell	1,432	1,275	1,125	931
Valid Barcodes	96.1%	96.6%	97.2%	97.2%
Sequencing Saturation	33.5%	36.5%	69.1%	34.0%
Q30 Bases in Barcode	95.9%	95.5%	96.0%	97.1%
Q30 Bases in RNA Read	93.9%	92.0%	92.8%	94.9%
Q30 Bases in UMI	95.5%	95.1%	94.0%	97.3%
Reads Mapped Confidently to Genome	88.6%	85.3%	90.0%	75.0%
Reads Mapped Confidently to Transcriptome	53.4%	52.6%	53.6%	53.5%
Reads Mapped Confidently to Exonic Regions	57.7%	56.2%	56.3%	56.1%
Reads Mapped Confidently to Intronic Regions	26.5%	23.9%	14.0%	14.8%
Reads Mapped Confidently to Intergenic Regions	4.4%	5.2%	19.7%	4.1%

Appendix Figure S1. Related to Figure 1. (A) Schematic depicting subcutaneous tumorigenesis and tissue harvesting for scRNA-seq. **(B-E),** Tumor tissue in MFC after 10days (B) , 20days (C) and B16 after 10days (D) and 20days (E) transplantation were harvested for analyses. Hematoxylineosin (HE) staining of tissues were shown. Scale bars 50 μm (black), 20μm (white). **(F)** Representative gating strategy for sorting tumor microenvironment single cells (FITC-Dead Cell Stain) for scRNA-seq. **(G)** Overall quality of scRNA-seq data of GC10d, GC20d, Mela10d and Mela 20d.

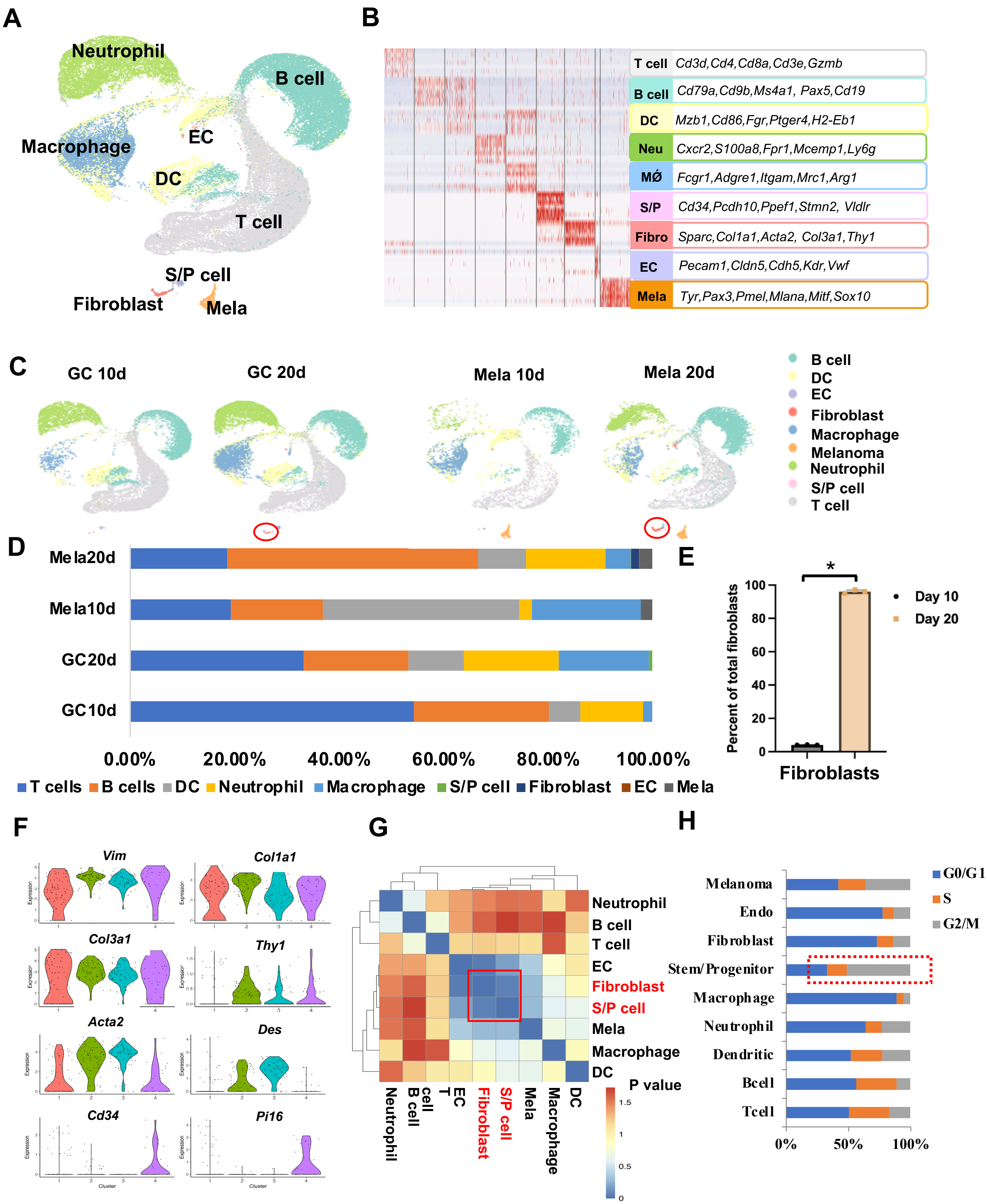

Appendix Figure S2. Related to Figure 1. (A) UMAP plots of the major cell types in melanoma microenvironment color-coded by cell types; **(B)** Heatmap of the selected marker gene expressions of each cell types; **(C)** UMAP plots of the cell types on 10 days (GC10d) or 20 (GC20d) days and B16 on 10 days (GC10d) or 20 days (GC20d); **(D)** Bar chart showing the proportion of major cell types in within 10-days and 20 days in subcutaneous tumorigenesis of MFC(GC) and B16 (Mela); **(E)** Bar graph showing the fibroblast in 10-days and 20 days subcutaneous tumor.Data represent mean ± SD; n = 3. Statistical significance was assessed using an unpaired two-tailed Student's t-test; Day 10 vs. Day 20, $P = 9.58 \times 10^{-9}$; *$P < 0.05$. **(F)** Violin plots of the normalized expression levels of selected representative genes of Pan-CAF (Col1a1, Vim, Col3a1 and Thy1), myCAF(Acta2, Des) and Cd34+CAF (Cd34, Pi16) in each fibroblast subcluster. **(G)** Clustering and heatmap of the similarities across total 9 dominant cell types. **(H).** Graph showing the proportion of cells in different cell cycle stage

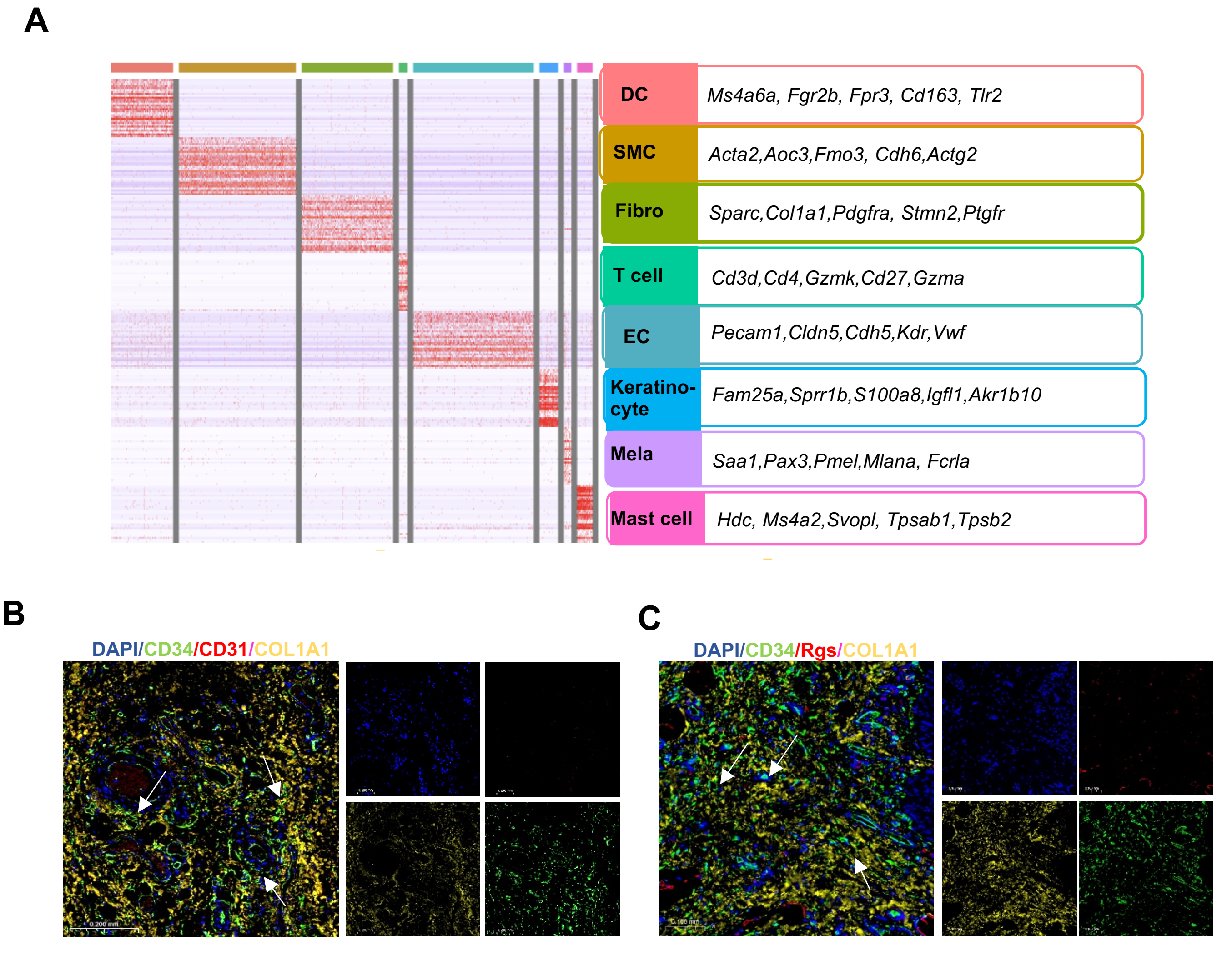

Appendix Figure S3. Related to Figure 1. **(A)** Heatmap of the selected marker gene expressions of each cell types **(B)** Images of Cd34, CD31 and Col1a1 in human gastric cancer samples. **(C)** Images of Cd34, Rgs5 and Col1a1 in human gastric cancer samples. Scale bars, white: 50μm, yellow:100 μm. Results show mean ± SD of 5 views. *P<0.05, unpaired Student's t test.

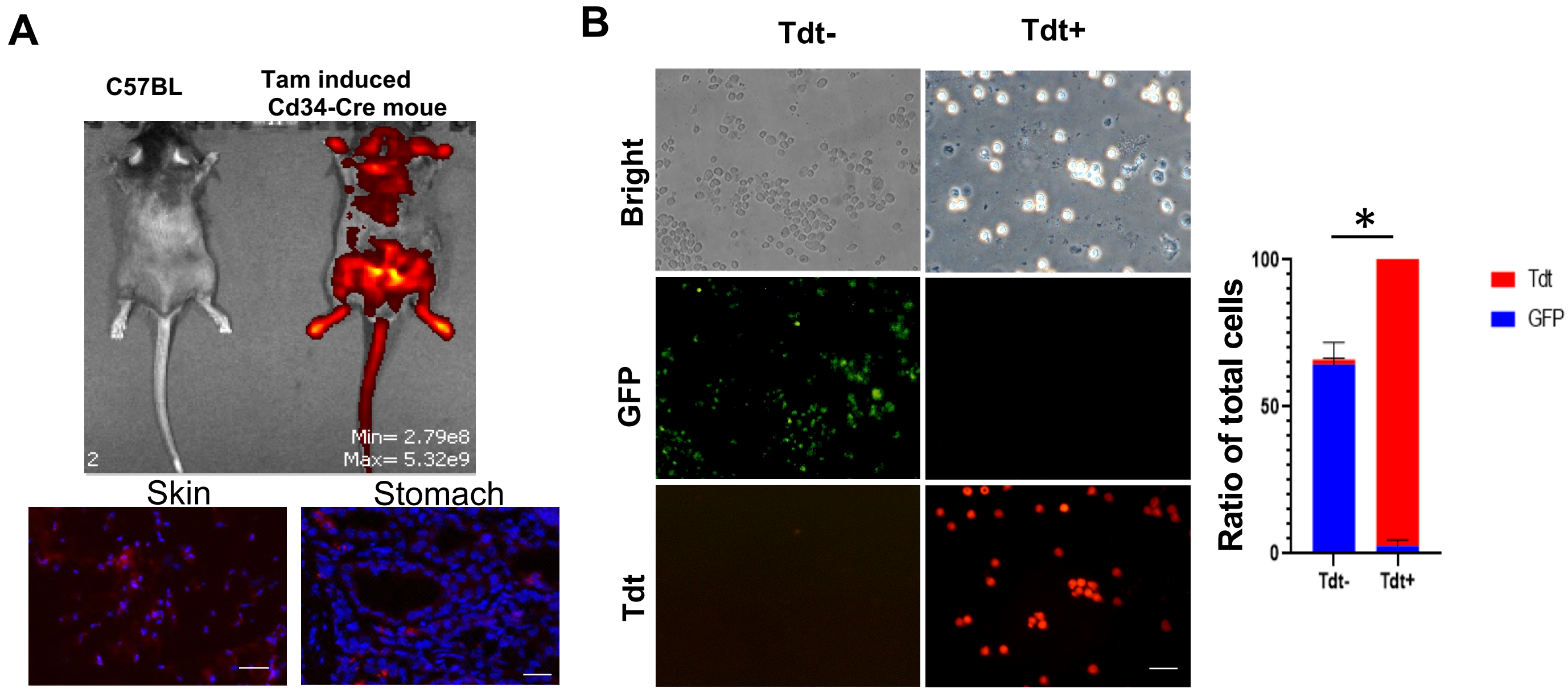

C

Parameter	Mela-Cd34	Mela-D	Mela-R
Estimated Number of Cells	16,304	9,140	11,797
Fraction Reads in Cells	96.5%	94.1%	95.0%
Mean Reads per Cell	16,719	34,364	30,210
Fraction of Reads kept after aggregation	100.0%	100.0%	100.0%
Median Genes per Cell	1,757	2,293	1,849
Valid Barcodes	96.8%	97.6%	97.5%
Sequencing Saturation	26.3%	42.8%	38.9%
Q30 Bases in Barcode	96.1%	94.7%	97.3%
Q30 Bases in RNA Read	94.7%	94.7%	95.5%
Q30 Bases in UMI	95.8%	97.2%	97.2%
Reads Mapped Confidently to Genome	90.6%	89.7%	90.5%
Reads Mapped Confidently to Transcriptome	73.0%	70.0%	72.3%
Reads Mapped Confidently to Exonic Regions	76.3%	73.3%	75.6%
Reads Mapped Confidently to Intronic Regions	10.2%	12.9%	11.9%
Reads Mapped Confidently to Intergenic Regions	4.1%	3.6%	3.1%

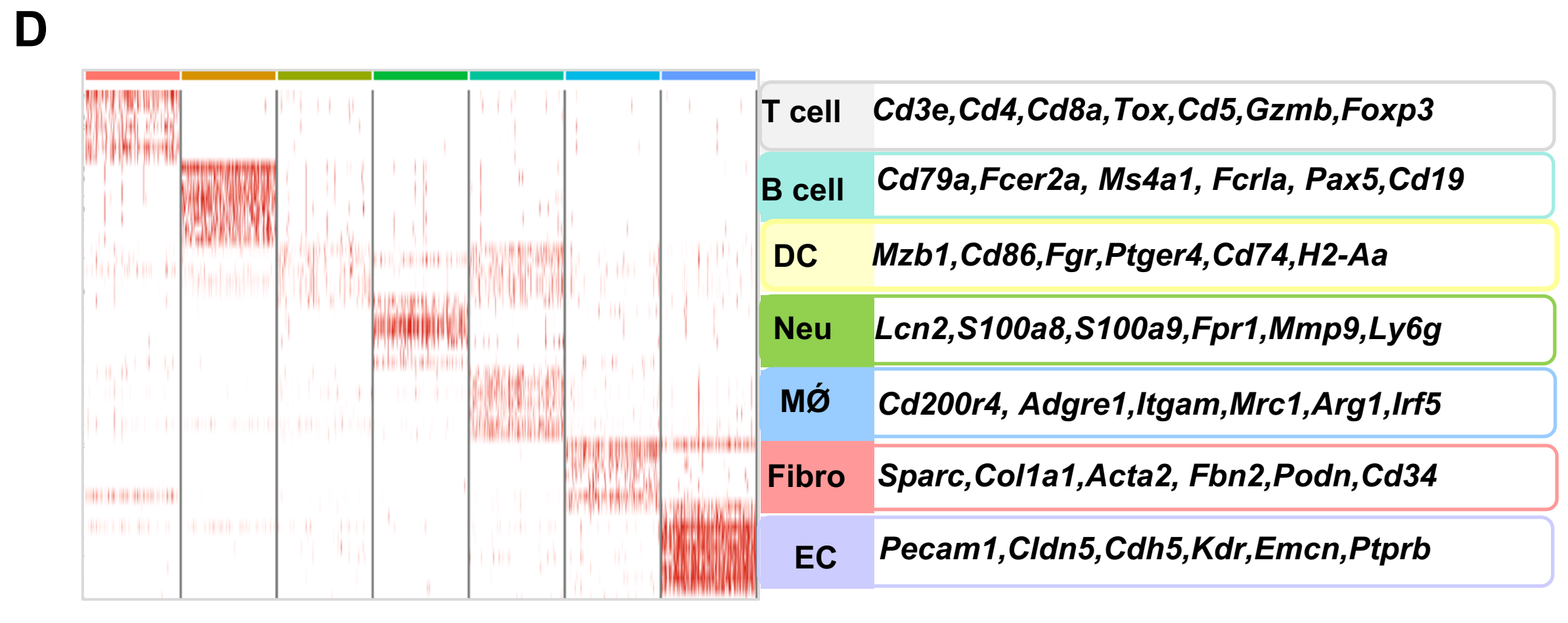

Appendix Figure S4. Related to Figure 2. (A) In vivo fluorescence Imaging of Cd34-CreER^{T2}; Rosa26-LSL-tdTomato mouse by Quantitative Fluorescence 3D Tomography System with 2 excitation laser channels(FMT 2000)(up). Tdtomato^{+} cell in skin and stomach tissue(down). Scale bars: 50 μm. **(B)** Immunostaining for flow cytometric analysis, sorted tdTomato cells and Bar chart**.** Data represent mean ± SD; n = 3 independent samples. Statistical significance was assessed using an unpaired two-tailed Student's t-test; $P = 1.88 \times 10^{-7}$; $^{*}P < 0.05$. **(C)** Overall quality of scRNA-seq data of different mouse models (Cd34-CreER^{T2}/ Rosa26-LSL-tdTomato mice (Mela-Cd34), as recipient (Mela-R) and as donor (Mela-D). **(D)** Heatmap of the top differential gene expression between each cell types.

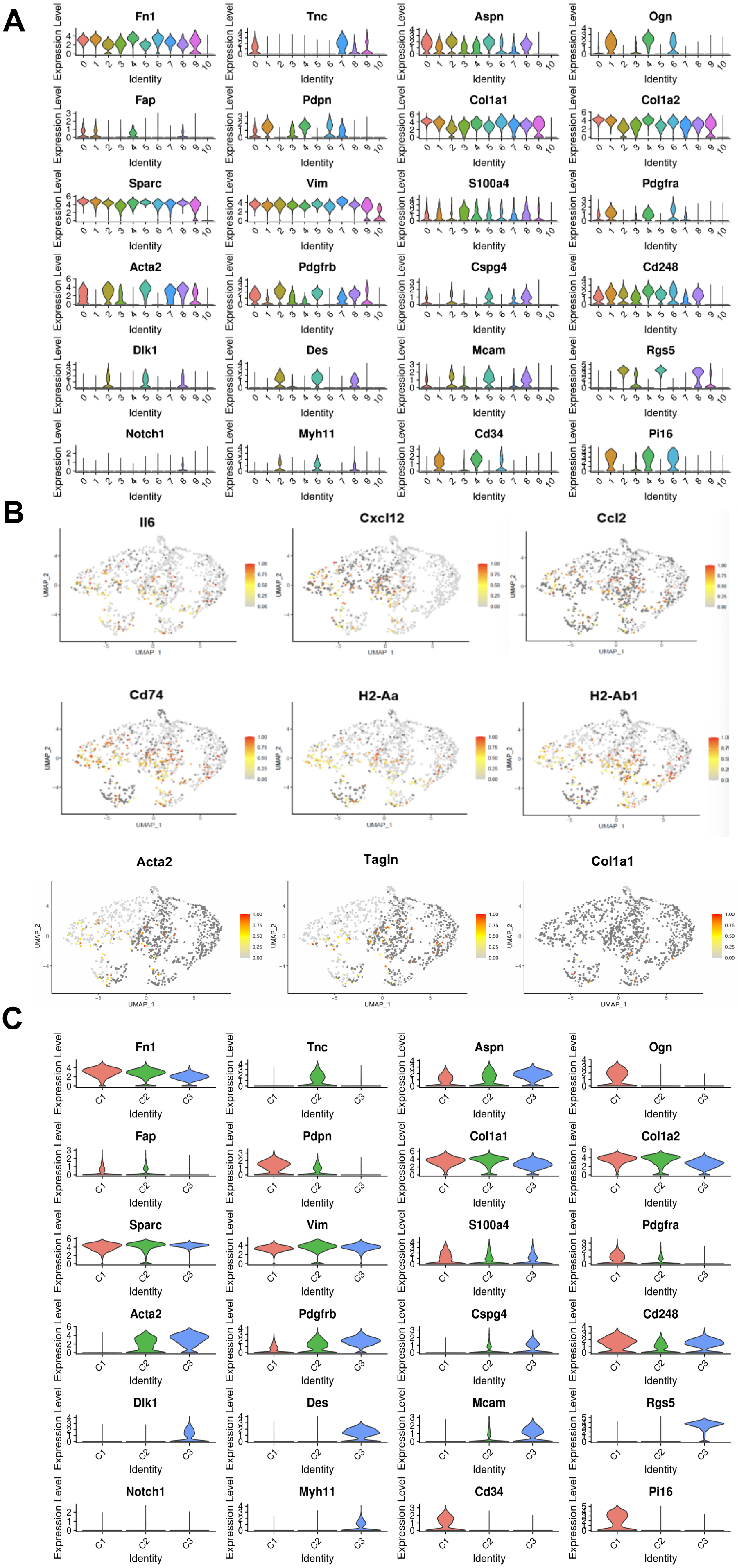

Appendix Figure S5. Related to Figure 2. (A) Comparison of expression levels of marker genes in each fibroblast subcluster. **(B)** Comparison of expression levels of iCAF, apCAF and myCAF marker genes in C1-3 fibroblast subcluster. **(C)** Comparison of expression levels of marker genes in C1-3 fibroblast subcluster.

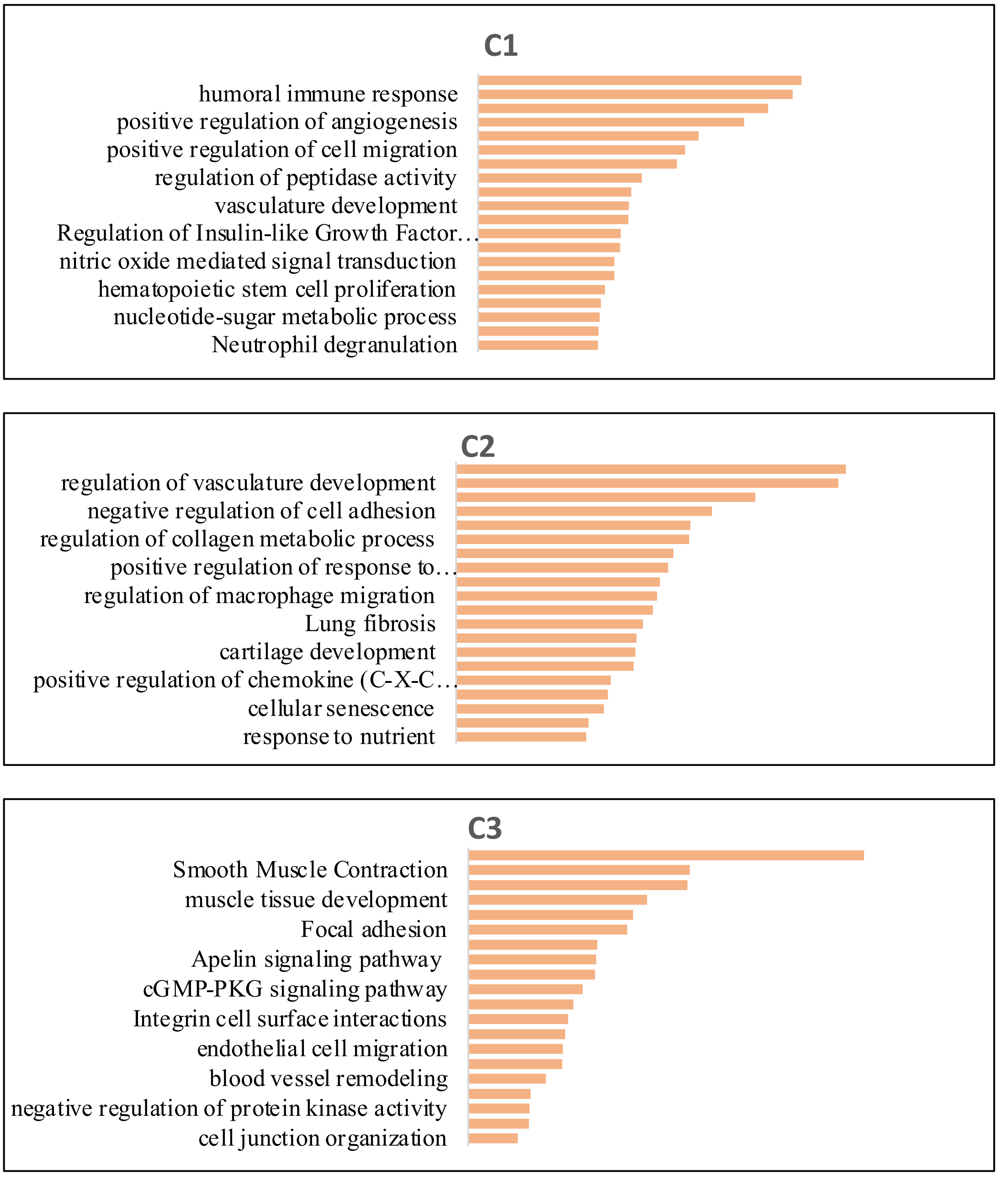

Appendix Figure S6. Related to Figure 2. Gene set enrichment analyses of genes in C1-3 subclusters howing biological processes.

Complete Blood Cell (CBC) Count

Indicator	NC	DT
RBC	10.3X10^6/mm^3±0.21E6/μL	10.1X10^6/mm^3±0.34E6/μL
Hb	13.2±0.51pg	12.9±0.67pg
WBC	7.62X10^3/mm^3±1.89E3/μL	7.71X10^3/mm^3±2.10E3/μL
PLT	743X10^3/mm^3±223E3/μL	729/mm^3±313E3/μL

Appendix Figure S7. Related to Figure 3. The complete blood count of mice before and after DT treatment.

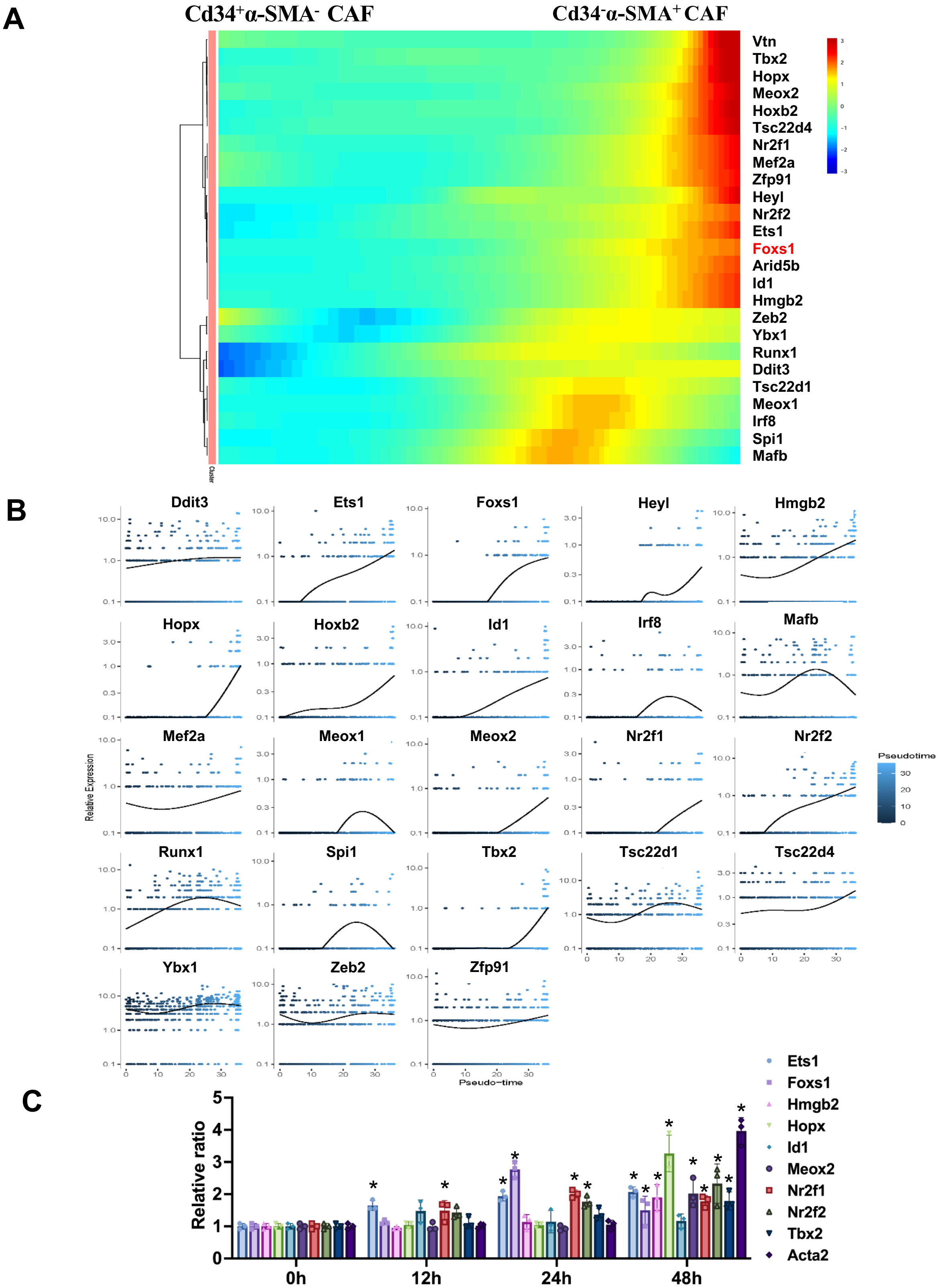

Appendix Figure S8. Related to Figure 4. (A) The heatmap illustrates the changes in transcription factors during the cellular transition process of Cd34^{+}α-SMA^{-} CAF into *Cd34*$^{-}$α-SMA^{+} CAFs by pseudo-temporal analysis. **(B)**Pseudo-temporal kinetics analysis using monocle algorithm was performed on the single-cell transcriptomics of CAF populations to assess the involvement of transcription factors in the cellular transition process. Cells characterized as Cd34^{+}α-SMA-CAF are represented in dark blue, whereas those identified as Cd34-α-SMA+CAFs are depicted in light blue. **(C)** CD34^{+} CAFs were cultured in vitro and induced with tumor-conditioned medium for 0, 12, 24, and 48 hours, followed by detection of various transcription factors and Acta2 expression. Data represent mean ± SD; n = 3 independent experiments. Statistical significance was assessed using ordinary two-way ANOVA followed by Dunnett's multiple comparisons test against the corresponding 0 h group. Significant adjusted P values were: Ets1: 0 h vs. 12 h, $P = 0.0050$; 0 h vs. 24 h, $P = 3.09 \times 10^{-5}$; 0 h vs. 48 h, $P = 2.38 \times 10^{-6}$. Foxs1: 0 h vs. 24 h, $P = 3.89 \times 10^{-13}$; 0 h vs. 48 h, $P = 0.0382$. Hmgb2: 0 h vs. 48 h, $P = 6.59 \times 10^{-5}$. Hopx: 0 h vs. 48 h, $P < 1 \times 10^{-17}$. Meox2: 0 h vs. 48 h, $P = 6.50 \times 10^{-6}$. Nr2f1: 0 h vs. 12 h, $P = 0.0433$; 0 h vs. 24 h, $P = 7.92 \times 10^{-6}$; 0 h vs. 48 h, $P = 0.00057$. Nr2f2: 0 h vs. 24 h, $P = 0.00076$; 0 h vs. 48 h, $P = 1.07 \times 10^{-8}$. Tbx2: 0 h vs. 48 h, $P = 0.00048$. Acta2: 0 h vs. 48 h, $P < 1 \times 10^{-23}$. *$P < 0.05$.

A

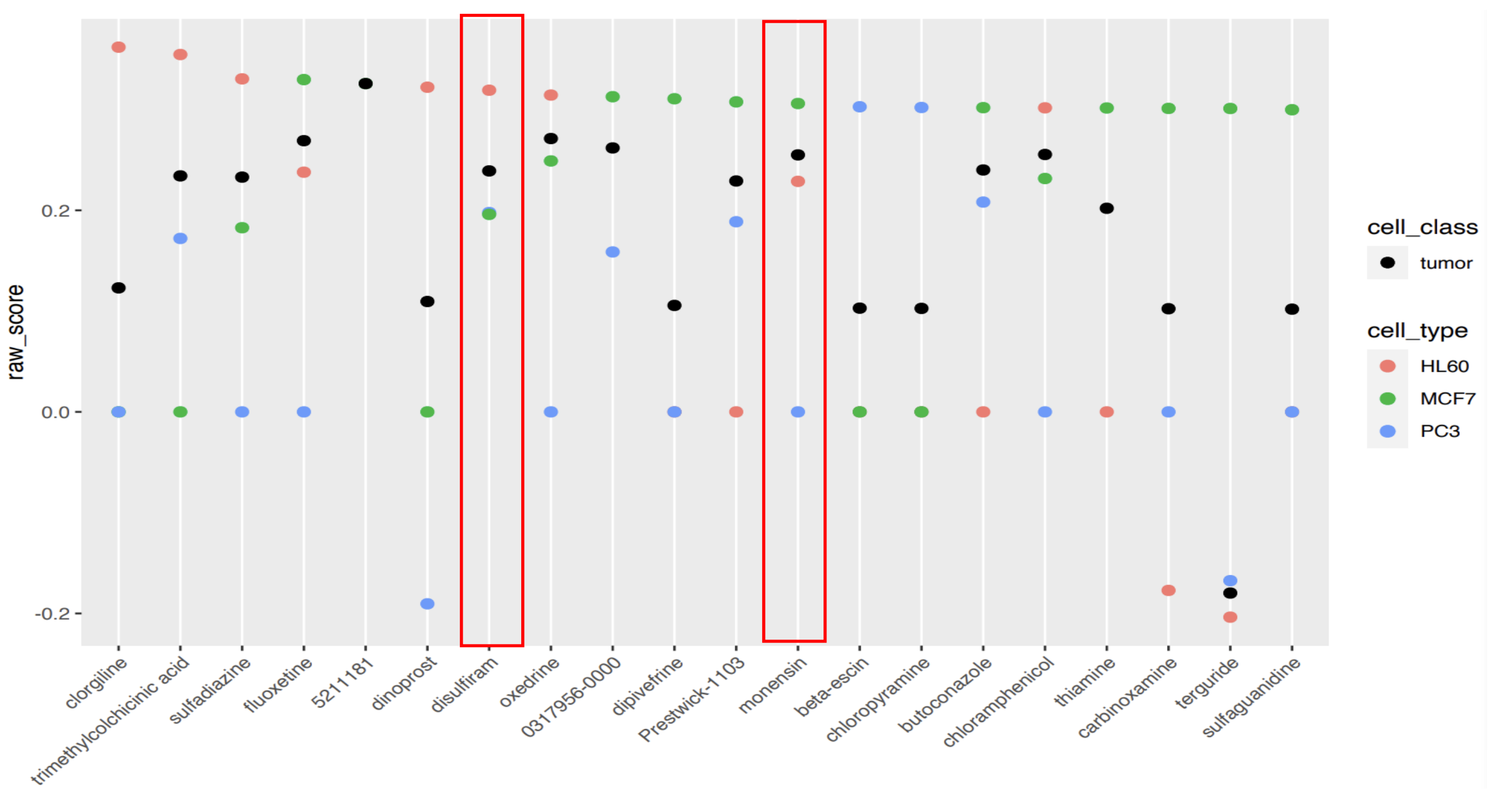

B

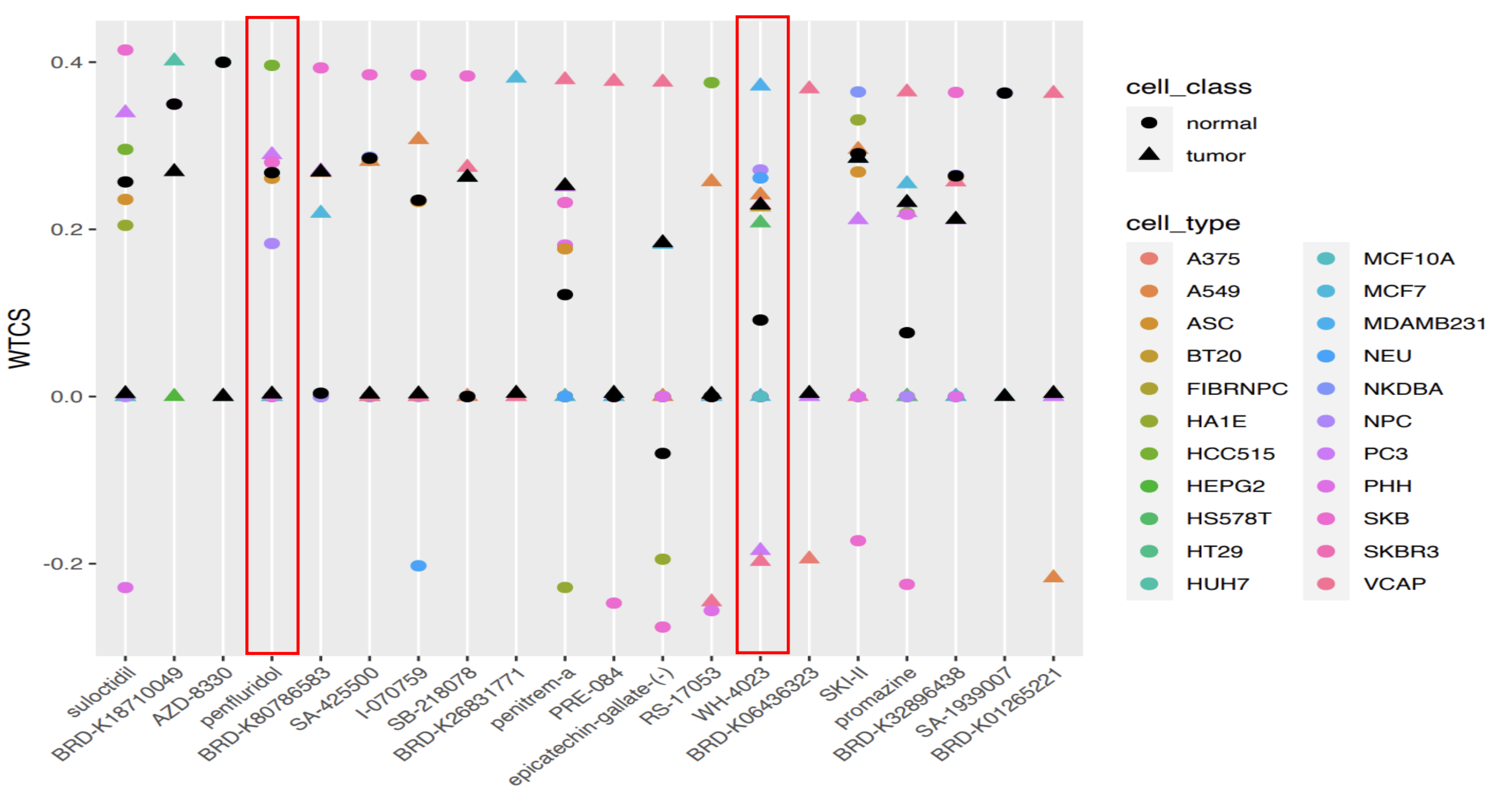

Appendix Figure S9. Related to Figure 5. (A) Top 20 drug candidates of CMap database with high signature-match scores in different cell lines. **(B)** Top 20 drug candidates of LINCS L1000 database with high signature-match scores in normal and tumor. Distinct colors represent different cell lines. The entire plot illustrates the degree of inhibition of this transition by various drugs across the different cell lines.

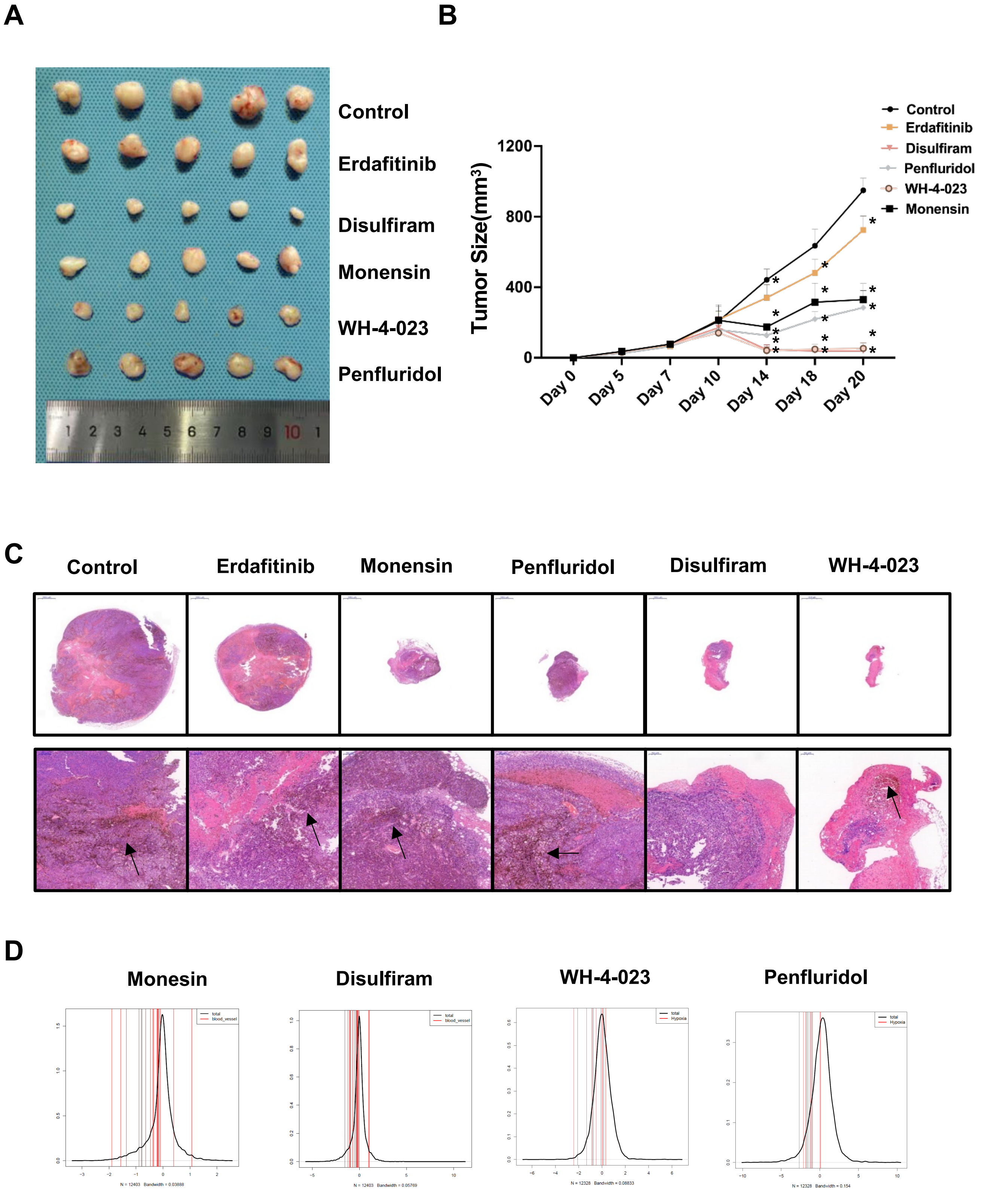

Appendix Figure S10. Related to Figure 5. (A) Photograph of subcutaneous gastric cancer after drug treatments. **(B)** Scatterplot for the changes of tumor volume(mm^3) in each group during gastric cancer development. Data represent mean ± SD; n = 5 mice per group. Statistical significance was assessed at each time point using ordinary one-way ANOVA followed by Bonferroni's multiple comparisons test against the control group.Day14: Control vs. Erdafitinib, $P = 0.0093$; Control vs. Disulfiram, $P = 4.14 \times 10^{-12}$; Control vs. Penfluridol, $P = 4.79 \times 10^{-10}$; Control vs. WH-4-023, $P = 3.15 \times 10^{-12}$; Control vs. Monensin, $P = 1.17 \times 10^{-8}$. Day18: Control vs. Erdafitinib, $P = 0.0093$; Control vs. Disulfiram, $P = 4.59 \times 10^{-12}$; Control vs. Penfluridol, $P = 6.61 \times 10^{-9}$; Control vs. WH-4-023, $P = 6.41 \times 10^{-12}$; Control vs. Monensin, $P = 7.95 \times 10^{-7}$. Day20: Control vs. Erdafitinib, $P = 0.0003$; Control vs. Disulfiram, $P = 1.73 \times 10^{-15}$; Control vs. Penfluridol, $P = 1.84 \times 10^{-12}$; Control vs. WH-4-023, $P = 2.46 \times 10^{-15}$; Control vs. Monensin, $P = 8.35 \times 10^{-12}$. * $P < 0.05$. **(C)** HE staining of tumor tissue of drug candidates treated mice. Comparison of expression levels of marker genes in each fibroblast subcluster. **(D)** The values of gene in a pathway of interest. Results show mean ± SD of 5 views. *$P<0.05$, unpaired Student's t test.

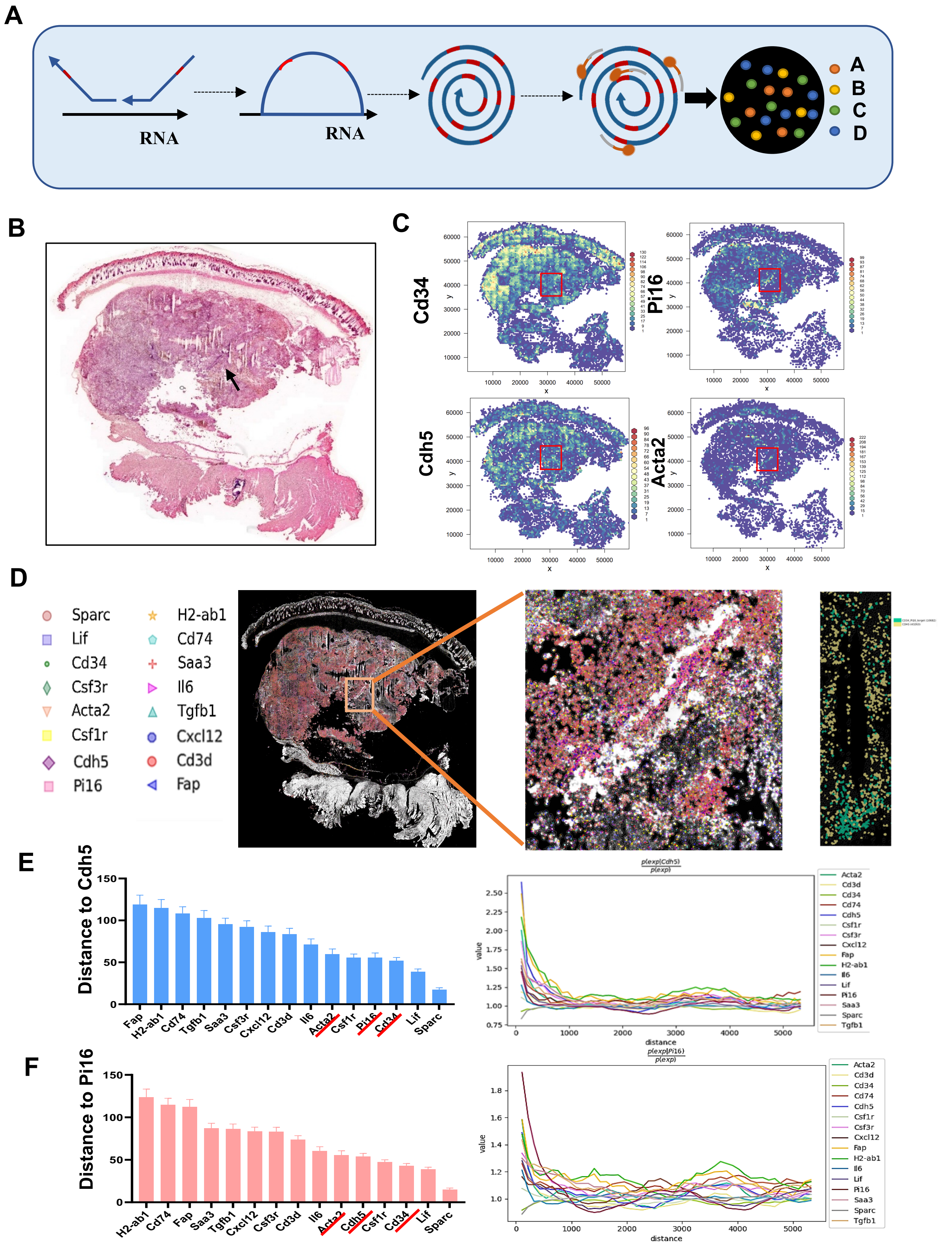

Appendix Figure S11. Spatial transcriptomic of mouse subcutaneous melanoma tissue using in situ sequencing. **(A)** Schematic diagram of in situ sequencing. **(B)** H&E-stained image of a fresh-frozen melanoma histological slide that was subjected to sequencing. **(C)** Overview of in situ mapping of *Cd34*, *Pi16*, *Cdh5* and *Acta2* transcripts. The red box highlights a signal-enriched area with blood vessel structure. **(D)** Localization of each of the 16 detected barcodes. Each symbol represents a barcode sequence that corresponds to a specific transcript. The red box is the same as in panel C. **(E)** Bar plots summarizing the statistics of the distances between the designated gene and the nearest *Cdh5*. **(F)** Bar plots summarizing the statistics of the distances between the designated gene and the nearest Pi16.
