# Supplementary material for: Foxs1-mediated transformation of CD34+ fibroblast to myCAFs promotes tumor growth
Source: EMBO Mol Med. 2026 Jul 4;18(8):3200–25. doi: 10.1038/s44321-026-00476-8 (PMC13469093; doi:10.1038/s44321-026-00476-8)
Supplement: Supplementary file 2 [file 44321_2026_476_MOESM2_ESM.pdf]

EMBO Molecular Medicine

Foxs1-mediated transformation of CD34+ fibroblast to myCAFs promotes tumor growth

Li Zhang, Junyao Yang, Ting Chen, Run Zhang, Jing Xu, Xinghe Zhao, Mei Yang, Zheyi Chen, Shasha Sun, Xiaocui Chen, Shuqiong Zhang, Liujun Jiang, Yingxia Zheng, Yanhua Hu, Qingbo Xu, Wei Lin, and Lisong Shen

Corresponding authors: Li Zhang (li.zhang.uk@gmail.com) , Lisong Shen (shenlisong@xinhuamed.com.cn), Wei Lin (21520@renji.com)

Review Timeline:

Submission Date:	7th Dec 24
Editorial Decision:	8th Jan 25
Appeal:	3rd Nov 25
Editorial Decision:	5th Dec 25
Revision Received:	4th Apr 26
Editorial Decision:	24th Apr 26
Revision Received:	27th May 26
Accepted:	15th Jun 26

Editor: Lise Roth

Transaction Report:

(Note: With the exception of the correction of typographical or spelling errors that could be a source of ambiguity, letters and reports are not edited. Depending on transfer agreements, referee reports obtained elsewhere may or may not be included in this compilation. Referee reports are anonymous unless the Referee chooses to sign their reports.)

8th Jan 2025

Decision on your manuscript EMM-2024-21040

Dear Prof. Zhang,

Thank you for the submission of your manuscript to EMBO Molecular Medicine. We have now received the feedback from the three referees who reviewed your manuscript.

As you will see from the reports below, while they acknowledge the potential interest of the findings, they also raise major concerns, including -but not limited to- unclear effect of decreased vascularization on tumor growth, limited number of isolated fibroblasts, poor writing and figure organization, unclear statistics, and are overall not convinced that the conclusions are fully supported by the data at this point.

Based on these concerns and given that at EMBO Press we encourage a single round of revisions in a limited time frame, I am afraid I see little choice but to return the manuscript to you at this point with the decision that we cannot offer to publish it.

I am very sorry to disappoint you in this occasion, and hope that the referees' comments are helpful in your continued work in this area.

Sincerely,

Lise Roth

Lise Roth
Senior Editor
EMBO Molecular Medicine

***** Reviewer's comments *****

Referee #1 (Comments on Novelty/Model System for Author):

See my second major concern below.

Referee #1 (Remarks for Author):

A potentially interesting paper that uncover CAF differentiation trajectories and identifies therapeutic interventions.

MAJOR CONCERNS
I would propose professional English editing be performed. For instance, the title should be made more concrete, and there are several grammar mistakes in the manuscript. Also, phrasing could be made more scientific ("as anticipation grows").

The CAF population seems to be very small in these tSNEs and 240 cells are not a lot to base conclusions on. Have the authors considered starting with human samples that likely contain a more relevant fraction of CAFs? As it stands now, the authors have done a good job at profiling the mouse model, *not* human cancer.

The interpretation of what S/P cells are and how they relate to CAFs confused me a bit. What are these S/P cells? Also relates to my next concern:

The lineage tracing coupled to scRNA-Seq is very elegant, but I am surprised to see CD34+ cells give rise to endothelium, CAFs, and immune cells. What does the tracing look like in the developing embryo and adult (healthy) mouse?

Figure 4 and interpretation; given that many cell types are CD34+ cells-derived and many also remain CD34-positivity, the DT treatment will ablate a very large number of cells. Therefore, the last sentence of the paragraph I do not understand; if macrophages derive from the CD34 lineage, they should have been ablated. As it currently appears, the method is either not specific, or not effective.

Figure 5E; how were these data obtained if anti-tumor efficacy (cf. Fig. 5C-D) was near-universal?

Stemness should be tested using functional assays for stemless rather than marker expression.

The work on Foxs1 seems unfinished. Rather than doing a reporter assay with a known promoter sequence, a genome wide pull-down would be a better experiment.

MINOR COMMENTS
The One Sentence Summary is hardly one sentence, it runs across 8 lines.

myCAFs are not universally accepted to be tumor-promoting.

"intuitive grouping" is not very scientific.

The drug is written MoneNsin.

I do not understand what is plotted in Fig. 5B.

Referee #2 (Remarks for Author):

Yang et al., indicated that CD34-positive progenitors differentiate into myCAFs via foxs1 upregulation during tumor progression. Inhibition of foxs1 expression by treatments with siRNA or chemical inhibitors suppressed the myCAF formation. Depletion of CD34-positive CAF progenitors attenuated growth of tumor cells, which were injected into recipient Cd34-DTR mice. Collectively, this work proposes that CD34-positive progenitors differentiate into myCAFs to contribute to tumor growth and progression. However, there are several issues to be resolved by the following.

Major critiques
1. How many myCAF proportions are derived from CD34-positive progenitors are not clearly demonstrated during the series of tumor progression. It would be important to quantify tdT and aSMA-double positive fibroblasts during different stages of tumors (e.g, 20, 40 days after injection) in Fig 2H. At same time, employing markers of inflammatory CAFs (iCAFs) and antigen-presenting CAFs (apCAFs), whether CD34-positive progenitors rarely contribute to iCAF and apCAF formation should be elucidated.
2. CD34-positive cells are generally known to serve as hematopoietic and endothelial progenitor cells. Depletion of CD34-positive cells is therefore anticipated to show loss of blood cells and vascular cells. Have the authors checked attenuation of these cells in cd34-DTR mice?
If blood cells and vascular cells are also suppressed, attenuated tumor growth in cd34-DTR mice might be due to loss of the blood cells and vascular cells.
3. Tumor growth should be shown in cd34-DTR without DT treatment, as the additional control for cd34-DTR treated with DT. Since there is no ROSA-DTR allele in Cd34-Cre mice.
4. Molecular mechanisms, by which foxs1 induces myCAF differentiation are not clearly demonstrated. Chromatin immunoprecipitation using anti-foxs1 antibody would be performed to determine whether foxs1 binding sites are located in the CD34 and aSMA gene regulatory sites to downregulate and upregulate their gene expressions, respectively.
5. Foxs1 protein expression has never been demonstrated in this paper. The Foxs1 protein expression should be confirmed.
6. Only one siRNA for foxs1 was used. To avoid the off-target effect, a few siRNAs need to be employed.
7. The author showed that culture medium conditioned by cancer cell lines induced foxs1 and aSMA expression in fibroblasts. Have the authors checked whether TGF-beta produced by cancer cell lines indeed contributes to the induction of foxs1 and aSMA expression?

Minor issues
1. There are a number of typos in the text. " ----- (Supplementary Figure6A) that ------" should be changed into " ----- (Supplementary Figure 6A) that ------", line 11, page 8.

Referee #3 (Comments on Novelty/Model System for Author):

It is not clear from the methods section or figure legends how many times each experiment was repeated.
Additionally, the ssRNAseq presented in FIg 1 includes very few fibroblasts

Referee #3 (Remarks for Author):

In their manuscript, Yang et al. propose that CD34+ mesenchymal cells could serve as potential progenitor cells for the

development of CAFs during tumor formation. They used multiple sequencing and spatial analysis methods as well as a wide set of in-vivo and in-vitro experiments to assess their theory.
Some of the findings in this paper showcase the fact that CD34 has a role in CAF transition, especially in the in-vitro studies. Nonetheless, the mouse model used includes all CD34 lineage cells and the images from the human tumors show mostly staining that seems like blood vessels. Hence, it is hard to exclude a role for pericytes and blood vessel development in the described findings, as an alternative explanation to the myCAF transition proposed.
Overall, although some of the findings show a clear role for CD34 and Foxs1 in tumor development and specifically in CAF phenotype, this study requires substantial additional experimental and analytical work before it can be considered for publication.
Major comments:
1) Figure 1: Why were the tumors injected subcutaneously and not orthotopically? In the case of Melanoma this makes sense, but for gastric cancer subQ is not the orthotopic location and CAFs could be very different there. Moreover, 4 mice/~240 fibroblasts is very little to establish any viable conclusions, why were only 240 fibroblasts left for analysis? Usually a yield of 10% of all cells is expected, especially when enriched for non-tumor cells. Given the fact that the authors later show results from human and CD34+ cells they should consider rearranging the presentation of the results.
Fig1D-E: The authors write "The high levels of Col1a1 and the mesenchymal marker Vim confirmed their fibroblast identity". This is not necessarily true, as Vim is known to be expressed in EMT cancer cells. The authors should use additional markers to substantiate the identity of these cell cluster.
Fig1F,G,I,J: CD34 staining seems to be primarily on blood vessels. This is a major issue as it may affect the interpretation of all results. The authors should validate these are not endothelial cells/pericytes, by adding markers such as MECA32/CD31+RGS5/MCAM.
Fig1H: There's very low correlation between the two groups, which does not point to a similarity as the authors suggest.
Fig1I-J: Para-GC/Mela means normal-adjacent?
Fig1J: What are the different channels in the staining (yellow/pink/red)
2. Following up on the pericyte issues, in Fig2 the authors write "Although the precise cellular identity of S3 (C3?) remains elusive, we lean towards the view that C3, which expresses pericyte markers, is a subtype of CAFs with a strong resemblance to myCAFs." Why? These seem like clear pericytes, what makes them myCAFs? Especially as C2 seems to have many more myCAF markers (ECM genes). Also, in Supp. Fig6D as the authors used in-situ sequencing to get a better resolution of colocalization of CD34 and other markers of interest it would be highly helpful for their message that they add single channels of each marker or at least some of the main ones to better convey the idea of CD34 CAFs/fibroblasts. The figure as it is now, is unreadable and the markers are hard to distinguish.
3. Supp 7, Fig3C-D - The authors write "To validate this phenotypic development, we isolated CD34+ fibroblasts from mouse tumor tissue and cultured them in differentiation medium with B16 culture medium for 14 days. Significant downregulation of CD34 and PI16 but upregulation of α-SMA were observed,..." (supp fig7) and the same assay was performed for gastric CAFs from human tumors. If the researchers used a differentiation medium to mimic the effects of cancer cells, it important to add a control condition where the medium is neutral and should not have an effect. In general, this is a problematic assay to test this specific theory as most fibroblasts grown in 2D for more than a few days will develop a myCAF like phenotype, this should be validated in other forms or in 3D culture.
4. Fig3A - The authors performed reanalysis of an existing scRNAseq data set that includes "230,000 fibroblasts across 17 tissues, 50 datasets, 11 disease states and 2 species", yet the analysis presented here includes only ~10,000 cells. Could the authors elaborate in the methods how was the dataset analyzed, which diseases/samples were included, how the cells were filtered etc.
5. Fig 4 - Generally, the idea behind the experiment presented is to show that depletion of CD34+ linage cells hinders formation of myCAFs later in tumor development and tumor growth in general. The major caveat of this approach is that the depletion of CD34+ cells depletes endothelial cells as well, which are highly important in tumor establishment and growth. The authors should add staining for CD31/MECA32 to show that the main cause of reduced tumor growth is not absence of vascularization. This will help strengthen their theory that the loss of myCAFs is the main cause for tumor reduction.
6. Fig5 - Overall the results are very promising, but again the authors should validate the cause of tumor reduction is not the absence of blood vessels formation, as they mentioned.
7. Fig6F-G - What is the difference between F and G, it is unclear from the figure legend. Furthermore, some of the graphs show significance but the effect is very minor, how was the significance tested? Also, some of the time points presented don't have error bars, does this mean there was only one sample for this time point?
8. Methods - In the fluorescence staining the authors did not mention how was the MxIF performed (which fluorescent reagents, on which channel) as the tumors express GFP it is important to make sure there is no clash with the staining. If the tumors are not GFP+ in this figure, how was the tumor/stroma areas defined.
9. Generally, it is not clear from the methods section or figure legends how many times each experiment was repeated.
Minor:
Linguistic editing is required throughout the manuscript.
Fig2B: What are the units in the y axis?
Supp Fig2B: y axis - Ratio is from 0 to 1, please change to percent.
Fig2G - In page 7 the authors write- "we intuitively regrouped them into 3 dominant subpopulations..." Intuitively is not an analytical method for clustering please write the method used for clustering the cells (Louvain or other..).

RE: EMM-2024-21040: Targeting Cd34+ fibroblast progenitors transition can dictate tumor fate

Dear Dr Lise Roth

Summary of the major changes and general response

We sincerely thank the editor for the time you have spent reviewing and considering our manuscript for publication,and also thank the reviewers for giving us constructive suggestions which would help us in depth to improve the quality of the paper. Here we submit a revised version of our manuscript with the title "Lineage Tracing and Drug Screening Identify a Foxs1-driven $Cd34^{+}$-to-$Acta2^{+}$ Transition of Cancer-associated Fibroblasts Promoting Tumor Growth", which has been modified according to the reviewers' and editor's suggestions. We do believe that the quality of the current article has been significantly improved.

The major revisions are summarized below:

1. We have now analyzed publicly available single-cell datasets derived from human melanoma lesions and from fibroblasts isolated from multiple normal human tissues. All datasets consistently resolved a cluster of $CD34^{+}Pi16^{+}$ fibroblasts that are transcriptionally distinct from endothelial cell and pericytes. Immunofluorescence staining of human melanoma sections further confirmed the presence of this population in situ. These new data are presented in revised Figure 1.
2. To further clarify the role of CD34+Pi16+ fibroblasts in tumor growth, we generated CD34/Pi16 dual-reporter mice and selectively ablated CD34+Pi16+ cells with diphtheria toxin. The efficiency of depletion was verified by flow cytometry. We then monitored melanoma growth and quantified the abundance of fibroblasts and angiogenesis within tumor tissues. The corresponding data are presented in Figure 3.
3. To define how stemness is altered and to identify the key molecular events during the differentiation of $CD34^{+}Pi16^{+}$ fibroblast progenitors into α-SMA^{+} myofibroblasts, we isolated $CD34^{+}Pi16^{+}$ cells from human tumor tissues and cultured them either in tumor-cell-conditioned medium or in medium supplemented with TGF-β to trigger differentiation. Clonogenic assays and telomere-length measurements were used to

monitor changes in stemness. For the candidate regulator Foxs1, we tracked its transcriptional and protein expression throughout the differentiation time-course. Chromatin immunoprecipitation (ChIP) and dual-luciferase reporter assays were employed to establish whether Foxs1 directly controls α-SMA expression; these findings were corroborated after Foxs1 knockdown. All related data are presented in Figure 4.

4. We re-evaluated the effects of four drugs on melanoma. To verify whether their inhibitory activity is attributable to suppression of angiogenesis, we examined CD31 staining and distribution; no significant difference in microvessel density was observed. All four agents down-regulated the expression of Foxs1 and α-SMA and reduced the number of α-SMA-positive fibroblasts. The corresponding data are presented in Figures 5–6.

Referee #1 (Comments on Novelty/Model System for Author):

See my second major concern below.

Referee #1 (Remarks for Author):

A potentially interesting paper that uncover CAF differentiation trajectories and identifies therapeutic interventions.

MAJOR CONCERNS

1. I would propose professional English editing be performed. For instance, the title should be made more concrete, and there are several grammar mistakes in the manuscript. Also, phrasing could be made more scientific ("as anticipation grows").

Response: Thank you for your critical comment. We have asked a professional English editor to edit the manuscript. We have changed the title accordingly: “Lineage tracing and drug screening identify a Foxs1-driven $Cd34^{+}$-to-$Acta2^{+}$ transition of cancer-associated fibroblasts promoting tumor growth”. In addition, we have rephrased some phrases.

2. The CAF population seems to be very small in these tSNEs and 240 cells are

not a lot to base conclusions on. Have the authors considered starting with human samples that likely contain a more relevant fraction of CAFs? As it stands now, the authors have done a good job at profiling the mouse model, *not* human cancer.

Response: We appreciate your thoughtful insights. To address the reviewers' concerns regarding the limited number of CAFs (previously ~240 cells), we incorporated publicly available single-cell RNA-seq datasets, including **GSE134388** for human melanoma and **GSE16729** for human gastric cancer. Following reanalysis using a standard scRNA-seq pipeline, we identified **1,234 fibroblast cells** in GSE134388, and **725** in GSE16729.

In both datasets, putative CAFs were re-clustered and the subtypes were annotated based on canonical markers, revealing consistent subtypes across datasets—namely, **CD34$^+$Pi16$^+$ CAFs** and **ACTA2$^+$ myCAFs**. These results substantially reinforce the robustness and reproducibility of our CAF-related findings, extending their relevance beyond the mouse model to include human tumor tissues. The relevant results are presented in Figure 1E, F(**GSE134388),** Figure 1G,H(**GSE16729)**.

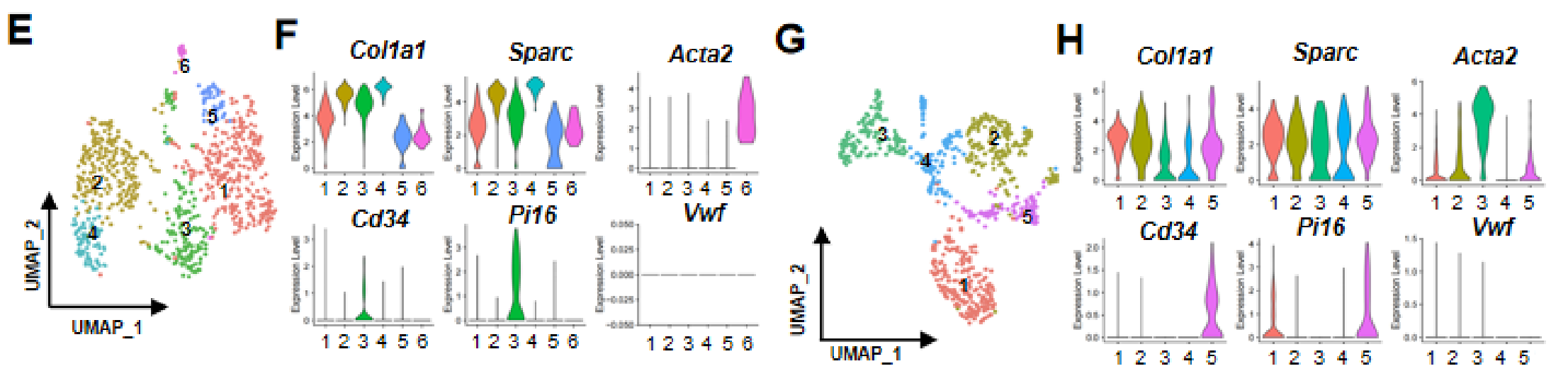

3. The interpretation of what S/P cells are and how they relate to CAFs confused me a bit. What are these S/P cells? Also relates to my next concern:

Response: Thank you for your professional comment. S/P cells is the abbreviation of stem/progenitor-like cells, which is a group of cells with high expression of stem cell genes (Cd34, Pcdh10 and Stmn2). We have already indicated it in the manuscript and highlighted in yellow.

To further elucidate how these cells with stem- or progenitor-like traits evolve within the tumor micro-environment, we developed a computational approach to

identify transcriptional similarity between cell type clusters in single-cell RNA sequencing (scRNA-seq) data, enabling the generation of biologically plausible lineage hypotheses based on transcriptomic profiles.The specific algorithmic steps have been supplemented in the methodology section "Similarity metrics of single-cell transcriptomes."

This approach enables the identification of transcriptionally similar cell populations that may share common developmental origins or functional characteristics. Clusters that appear closely related in the hierarchical structure suggest potential lineage relationships or shared regulatory programs. The method provides a quantitative framework for generating testable hypotheses about cellular differentiation trajectories and developmental hierarchies based on transcriptomic similarity patterns. The clustering-based similarity analysis is particularly valuable for:

1. Identifying putative progenitor-progeny relationships.
2. Discovering shared regulatory networks between cell types.
3. Validating clustering results and identifying potential misclassifications.
4. Generating hypotheses for lineage tracing experiments.
5. Understanding the transcriptional landscape of complex tissues.

This computational framework provides a robust foundation for exploring cellular relationships in single-cell transcriptome data and generating biologically meaningful hypotheses about cell type evolution and differentiation pathways.

Algorithm Implementation

The method employs a multi-step computational pipeline that leverages principal component analysis (PCA) and hierarchical clustering to quantify inter-cluster relationships:

1. Dimensionality Reduction: The algorithm extracts the first 20 principal components from the PCA reduction of the Seurat object, capturing the major sources of transcriptional variation while reducing computational noise.
2. Cluster Centroid Calculation: For each cell type cluster, we compute the mean expression profile in the PCA space by averaging the principal component scores

across all cells within each cluster, generating representative centroids that summarize the transcriptional characteristics of each cell population.

3. Similarity Matrix Construction: Pairwise similarities between cluster centroids are calculated using cosine similarity, which measures the cosine of the angle between two vectors in the high-dimensional PCA space. This metric is particularly suitable for transcriptional data as it captures the direction of gene expression patterns rather than absolute expression levels.

4. Distance-based Clustering: The cosine similarity matrix is converted to a distance matrix (distance = 1 - similarity) and subjected to hierarchical clustering using the McQuitty method, which generates a dendrogram representing the hierarchical relationships between cell type clusters.

5. Visualization: The resulting distance matrix is visualized as a heatmap with hierarchical clustering dendrograms, where darker colors indicate higher similarity (lower distance) between clusters.

4. The lineage tracing coupled to scRNA-Seq is very elegant, but I am surprised to see CD34+ cells give rise to endothelium, CAFs, and immune cells. What does the tracing look like in the developing embryo and adult (healthy) mouse ?

Response: We appreciate the reviewer's critical comment. Recently we have performed a series of experiment tracing $CD34^+$ cell during mouse embryonic development. We found that $CD34^+$ cells differentiate into endothelial cells at days 6.5, inflammatory cells at days 9.5 and fibroblasts at days 12.5 (see below)

Figure for reviewers removed

In adult, we have collected the single cell RNA sequencing data from us and others and found that $CD34^+$ cells can differentiate into endothelial cells, inflammatory cells and fibroblasts as well (see below Figure).

Figure for reviewers removed

These results will be presented and described in a separate paper.

5. Figure 4 and interpretation; given that many cell types are CD34+ cells-derived and many also remain CD34-positivity, the DT treatment will ablate a

very large number of cells. Therefore, the last sentence of the paragraph I do not understand; if macrophages derive from the CD34 lineage, they should have been ablated. As it currently appears, the method is either not specific, or not effective.

Response: We thank the reviewer for this insightful comment. The specificity of the ablation is indeed a crucial point, and it is maintained due to the combined effects of genetic design, temporal control, and cellular ontogeny. To specifically target the $Cd34^+$ fibroblast subset, we generated Cd34-Dre; Pi16-Cre system which targets the $Cd34^+Pi16^+$ fibroblast progenitor lineage, not all CD34-derived cells. This strategy markedly reduces off-target effects, limiting toxicity to the fibroblast-progenitor differentiation pathway. After selective ablation, tumor growth was restrained to varying degrees (Figure 3I–K).

Our response addresses several key aspects: First, as the reviewer rightly implies, Cre-mediated recombination in complex transgenic models is never 100% efficient. More importantly, the system is temporally controlled. Tamoxifen induction creates a finite "pulse" of labeling and DTR expression in the $Cd34^+Pi16^+$ population existing during that ~5-day window. Only these cells are susceptible to subsequent DT ablation. After tamoxifen clearance, newly emerging $CD34^+$ cells are not targeted, allowing for replenishment. Meanwhile，our single-cell data (Fig. 2C, D) clearly show that while the $CD34^+$ lineage gives rise to diverse cells, only endothelial cells and a subset of fibroblasts retain CD34 expression. Differentiated immune cells (T cells, B cells, macrophages) found in the tumor have downregulated CD34 and, critically, would have also downregulated the progenitor marker Pi16. Since DT ablation requires the expression of DTR driven by both Cd34-Dre and Pi16-Cre, these mature, $CD34^-$ immune cells are spared. Finally, tissue-resident macrophages and those recruited from the circulation can originate from various sources, not exclusively from the $Cd34^+Pi16^+$ progenitor pool targeted by our model. Therefore, even a highly efficient ablation of this specific lineage would not be expected to deplete the entire macrophage population, as observed.

6. Figure 5E; how were these data obtained if anti-tumor efficacy (cf. Fig. 5C-D) was near-universal?

Response: We completely agree with the reviewer. We apologize for not providing a clear explanation of the results. Because of sub-optimal lighting during the initial imaging, very small tumors were poorly visible. We have now repeated the experiment under improved illumination, obtained clearer results, and have replaced the original images accordingly (Figure3D in revised manuscript).

7.Stemness should be tested using functional assays for stemless rather than marker expression.

Response: We are grateful for your positive comments and your recognition. To further confirm the difference in stemness between $CD34^{+}Pi16^{+}$ fibroblasts and their differentiated $CD34^{-}Acta2^{+}$ progeny, we added clonogenic assays. These assays showed that the colony-forming capacity of $CD34^{+}Pi16^{+}$ cells progressively declined upon exposure to tumor-cell-conditioned medium or TGF-β, and was markedly reduced once the cells differentiated into $CD34^{-}Acta2^{+}$ fibroblasts (Figure 4A). In addition, we measured telomere length and found that both tumor-cell-conditioned medium and TGF-β significantly shortened telomeres in $CD34^{+}Pi16^{+}$ cells. Collectively, these data indicate that $CD34^{+}Pi16^{+}$ fibroblasts possess greater stem-like properties (Figure 4B).

8.The work on Foxs1 seems unfinished. Rather than doing a reporter assay with a known promoter sequence, a genome wide pull-down would be a better experiment.

Response: Thank you very much for your valuable suggestion. We have now performed additional ChIP assays with anti-foxs1, predicted the binding sites, and validated them by qPCR. The results demonstrate that Foxs1 binds directly to the promoter region of Acta2 and regulates its transcription. Details are presented in Figure 4J.

MINOR COMMENTS

The One Sentence Summary is hardly one sentence, it runs across 8 lines.

Response: Sorry for that. We have deleted One Sentence Summary.

myCAFs are not universally accepted to be tumor-promoting.

Response: Thank you for your advice. Indeed, myCAFs are not universally accepted as tumor-promoting. We corrected the inaccurate statement and changed it to: α-SMA^{+} CAFs likely correspond to myCAFs, play an integral role in the tumor growth.

"intuitive grouping" is not very scientific.

Response: Thank you for your insightful question. We apologize for the inaccuracy in our previous statement. We have revised it accordingly.

The drug is written MoneNsin.

Response: I'm sorry. I don't quite understand your question. Monensin is a polyether isolated from Streptomyces cinnamonensis that presents antibiotic properties. It is widely used in ruminant animal feeds.

I do not understand what is plotted in Fig. 5B.

Response: We performed a search against the perturbagen databases of CMap and LINCS L1000 to prioritize the drug candidates that potentially inhibit CAF transition. For such a search, we chose the top 20 candidates of each, and conducted validation experiments.

In this figure: Circles denote normal cell lines, while triangles indicate tumor cell lines. Distinct colors represent different cell lines. The entire plot illustrates the degree of inhibition of this transition by various drugs across the different cell lines. In the revised manuscript, these results have been moved to the supplementary materials (Supplementary Figure 8).

Referee #2 (Remarks for Author):

Yang et al., indicated that CD34-positive progenitors differentiate into myCAFs via foxs1 upregulation during tumor progression. Inhibition of foxs1 expression by treatments with siRNA or chemical inhibitors suppressed the myCAF formation. Depletion of CD34-positive CAF progenitors attenuated growth of tumor cells, which were injected into recipient Cd34-DTR mice. Collectively, this work proposes that CD34-positive progenitors differentiate into myCAFs to contribute to tumor growth and progression. However, there are several issues to be resolved by the following.

Major critiques

1. How many myCAF proportions are derived from CD34-positive progenitors are not clearly demonstrated during the series of tumor progression. It would be important to quantify tdT and aSMA-double positive fibroblasts during different stages of tumors (e.g, 20, 40 days after injection) in Fig 2H. At same time, employing markers of inflammatory CAFs (iCAFs) and antigen-presenting CAFs (apCAFs), whether CD34-positive progenitors rarely contribute to iCAF and apCAF formation should be elucidated .

Response: Thank you for your detailed comments. We have quantified the proportion of tdT+ aSMA+ double-positive fibroblasts, as shown in Figure 2J. Additionally, apCAFs and iCAFs did not form distinct clusters within the CD34-derived cells (see Supplementary Figure 5C). CD34-positive progenitors rarely contribute to iCAF and apCAF formation.

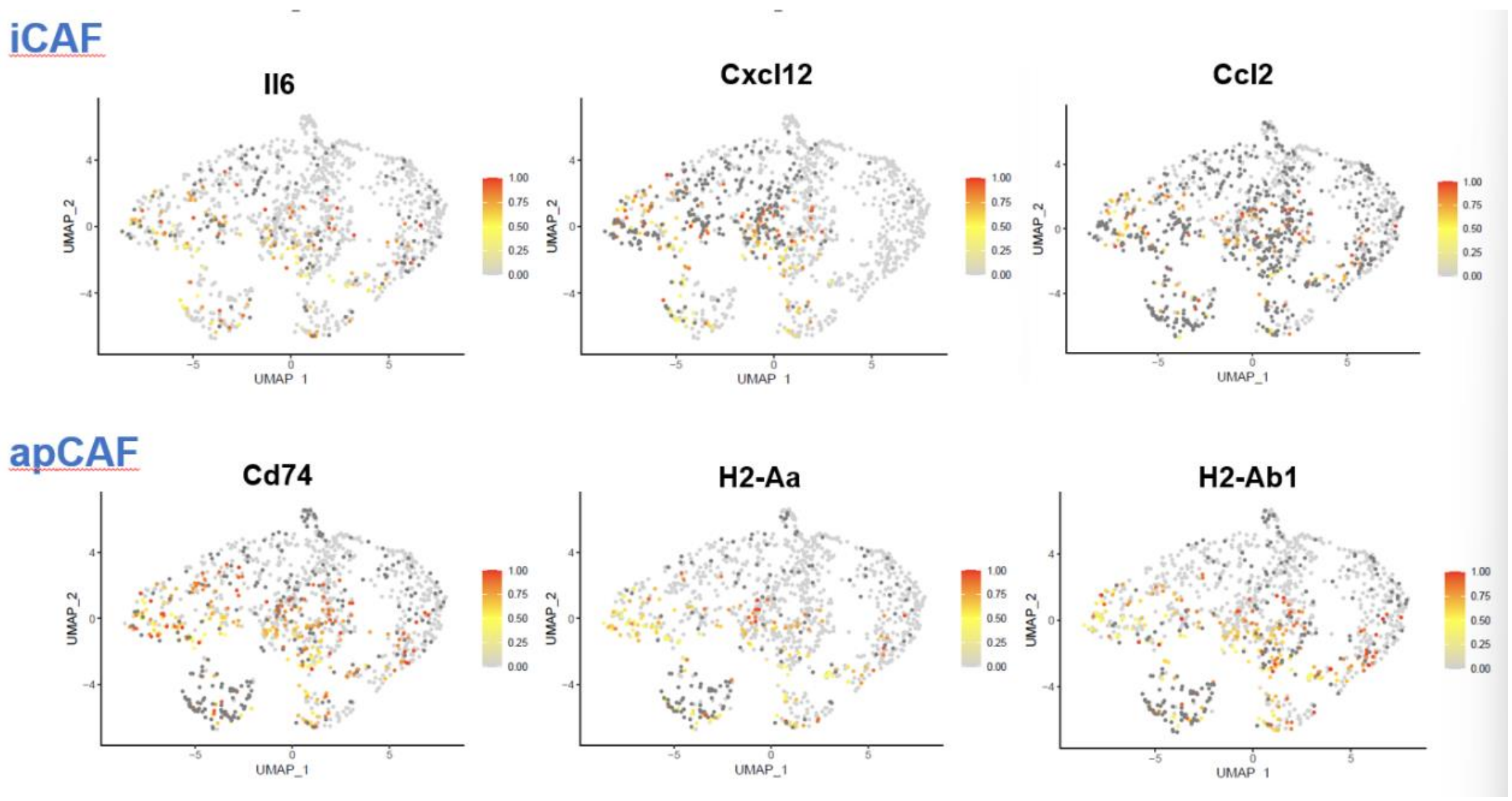

2. CD34-positive cells are generally known to serve as hematopoietic and endothelial progenitor cells. Depletion of CD34-positive cells is therefore anticipated to show loss of blood cells and vascular cells. Have the authors checked attenuation of these cells in cd34-DTR mice?

If blood cells and vascular cells are also suppressed, attenuated tumor growth in cd34-DTR mice might be due to loss of the blood cells and vascular cells.

Response: We completely agree with the reviewer. Indeed, cells of the CD34 lineage are quite heterogeneous. Depleting all CD34-lineage cells would affect a subset of hematopoietic and vascular cells, introducing considerable uncertainty in tumor growth. To specifically target the $Cd34^{+}$ fibroblast subset, we generated $CD34^{+}Pi16^{+}$ cell double-positive mice and administered diphtheria toxin (DT) to ablate only the $CD34^{+}Pi16^{+}$ population. This strategy markedly reduces off-target effects, limiting toxicity to the fibroblast-progenitor differentiation pathway. After selective ablation, tumor growth was restrained to varying degrees. Interestingly, although the overall number of intratumoral endothelial cells remained unchanged, the vessels that did form were predominantly small-caliber; vessel density increased, yet large, fully structured vessels were scarce (Figure 3I–K).

Besides, we examined the complete blood count of mice before and after DT treatment. After DT administration, $CD34^{+}Pi16^{+}$cells were ablated, whereas

erythrocyte, leukocyte and hemoglobin levels showed no significant differences (Supplementary Figure 7).

Indicator	NC	DT
**RBC**	$10.3X10^6/mm^3 \pm 0.21E6/\mu L$	$10.1X10^6/mm^3 \pm 0.34E6/\mu L$
**Hb**	13.2±0.51pg	12.9±0.67pg
**WBC**	$7.62X10^3/mm^3 \pm 1.89E3/\mu L$	$7.71X10^3/mm^3 \pm 2.10E^3/\mu L$
**PLT**	$743X10^3/mm^3 \pm 223E3/\mu L$	$729X10^3/mm^3 \pm 313E^3/\mu L$

3. Tumor growth should be shown in cd34-DTR without DT treatment, as the additional control for cd34-DTR treated with DT. Since there is no ROSA-DTR allele in Cd34-Cre mice.

Response: Thank you for your valuable suggestion. We have now added the requested group to Figure 3D-K.

4. Molecular mechanisms, by which foxs1 induces myCAF differentiation are not clearly demonstrated. Chromatin immunoprecipitation using anti-foxs1 antibody would be performed to determine whether foxs1 binding sites are located in the CD34 and aSMA gene regulatory sites to downregulate and upregulate their gene expressions, respectively.

Response: We appreciated your comments. Chromatin immunoprecipitation using anti-foxs1 antibody has been performed to determine whether foxs1 binding sites are located in the Acta2 mRNA regulatory sites. The results showed that foxs1 binding sites are located in the Acta2 mRNA regulatory sites, and upregulate its gene expressions. While foxs1 binding sites are located in the Acta2 mRNA regulatory sites. (Figure 4J)

5. Foxs1 protein expression has never been demonstrated in this paper. The Foxs1

protein expression should be confirmed .

Response: Thank you for your professional comments. We have examined the expression of the FOXS1 protein under various differentiation conditions as requested and found that it mirrors the mRNA expression results. Notably, one day after stimulation with gastric cancer medium, the protein level increased significantly. Details are shown in Figure 4G,L.

6. Only one siRNA for foxs1 was used. To avoid the off-target effect, a few siRNAs need to be employed.

Response: We designed three distinct siRNAs and confirmed their efficacy in functional assays; the results are presented in Figure 4F, G.

7. The author showed that culture medium conditioned by cancer cell lines induced foxs1 and aSMA expression in fibroblasts. Have the authors checked whether TGF-beta produced by cancer cell lines indeed contributes to the induction of foxs1 and aSMA expression?

Response: Thank you for your professional comments. We examined the effect of TGF-β on this differentiation process and found that it can indeed induce up-regulation of FoxS1 and α-SMA, although the magnitude of induction was smaller than that observed with tumor cell-conditioned medium. This indicates that additional factors present in the tumor-conditioned medium also contribute to the differentiation. The detailed results are shown in Figure 4.

Minor issues

1. There are a number of typos in the text. " ----- (Supplementary Figure6A) that ------ " should be changed into " ----- (Supplementary Figure 6A) that ------", line 11, page 8.

Response: I sincerely apologize—the revisions have now been completed exactly as requested.

Referee #3 (Comments on Novelty/Model System for Author):

It is not clear from the methods section or figure legends how many times each experiment was repeated.

Additionally, the ssRNAseq presented in FIg 1 includes very few fibroblasts.

Response: We appreciate your thoughtful insights. We have added details such as the number of experimental replicates in the Methods or figure legends.

To address the reviewers' concerns regarding the limited number of CAFs (previously ~240 cells), we incorporated publicly available single-cell RNA-seq datasets, including GSE134388 for human melanoma and GSE16729 for human gastric cancer. Following reanalysis using a standard scRNA-seq pipeline, we identified **1,234** fibroblast cells in GSE134388, and **725** in GSE16729.

In both datasets, putative CAFs were re-clustered and the subtypes were annotated based on canonical markers, revealing consistent subtypes across datasets—namely, CD34$^+$Pi16$^+$ CAFs and ACTA2$^+$ CAFs. These results substantially reinforce the robustness and reproducibility of our CAF-related findings, extending their relevance beyond the mouse model to include human tumor tissues. The relevant results are presented in Figure 1E, F(GSE134388), Figure 1G, H(GSE16729).

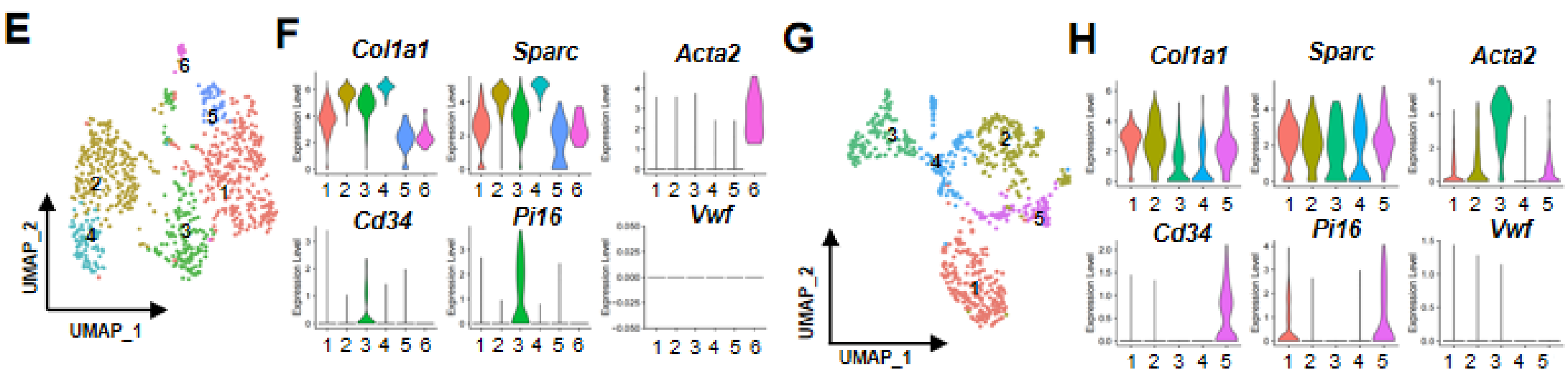

Referee #3 (Remarks for Author):

In their manuscript, Yang et al. propose that CD34+ mesenchymal cells could serve as potential progenitor cells for the development of CAFs during tumor formation. They used multiple sequencing and spatial analysis methods as well as a wide set of in-vivo and in-vitro experiments to assess their theory.

Some of the findings in this paper showcase the fact that CD34 has a role in CAF

transition, especially in the in-vitro studies. Nonetheless, the mouse model used includes all CD34 lineage cells and the images from the human tumors show mostly staining that seems like blood vessels. Hence, it is hard to exclude a role for pericytes and blood vessel development in the described findings, as an alternative explanation to the myCAF transition proposed.

Overall, although some of the findings show a clear role for CD34 and Foxs1 in tumor development and specifically in CAF phenotype, this study requires substantial additional experimental and analytical work before it can be considered for publication.

Major comments:

1) Figure 1: Why were the tumors injected subcutaneously and not orthotopically? In the case of Melanoma this makes sense, but for gastric cancer subQ is not the orthotopic location and CAFs could be very different there. Moreover, 4 mice/~240 fibroblasts is very little to establish any viable conclusions, why were only 240 fibroblasts left for analysis? Usually a yield of 10% of all cells is expected, especially when enriched for non-tumor cells. Given the fact that the authors later show results from human and CD34+ cells they should consider rearranging the presentation of the results.

Response: Thank you for your detailed comments. Indeed, the tumor micro-environment differs between subcutaneous and orthotopic gastric-cancer models. Our actual sequencing data show that the fibroblasts were derived primarily from 20-day melanomas. Subsequent work therefore focused on melanoma, using the gastric-cancer model mainly as a complementary platform for drug testing. Because no enrichment was performed initially, fibroblast numbers in subcutaneous mouse tumors were low; we later implemented CD34-based enrichment to increase their relative abundance. In addition, we incorporated human melanoma and gastric cancer data, shown in Figure1D–H.

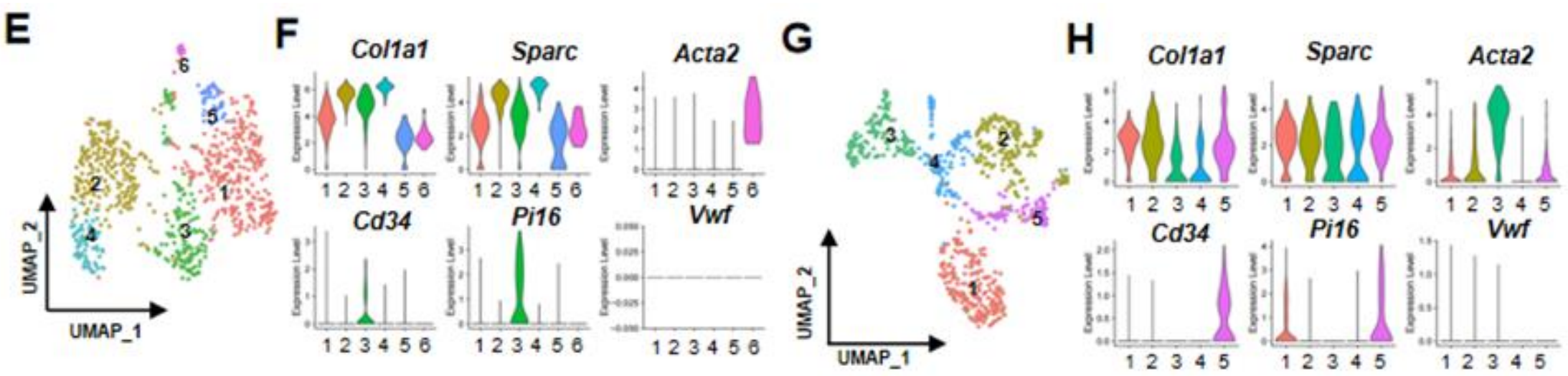

Fig1D-E: The authors write "The high levels of Col1a1 and the mesenchymal marker Vim confirmed their fibroblast identity". This is not necessarily true, as Vim is known to be expressed in EMT cancer cells. The authors should use additional markers to substantiate the identity of these cell cluster.

Response: We appreciate your thoughtful insights. Although vimentin is widely used as one of the classic markers of fibroblasts, it is not exclusive to them; it is also expressed in other mesenchymal-cell types and in tumor cells. Consequently, vimentin alone cannot specifically distinguish fibroblasts from other cell types. In practice, vimentin is usually combined with markers such as Dcn, Col1a1 and CD90 (Thy1) to increase the specificity of fibroblast identification. For this reason, we used Vim, Col1a1, Thy1 and Col3a1 to confirm the fibroblast population (Supplementary Figure2F) .

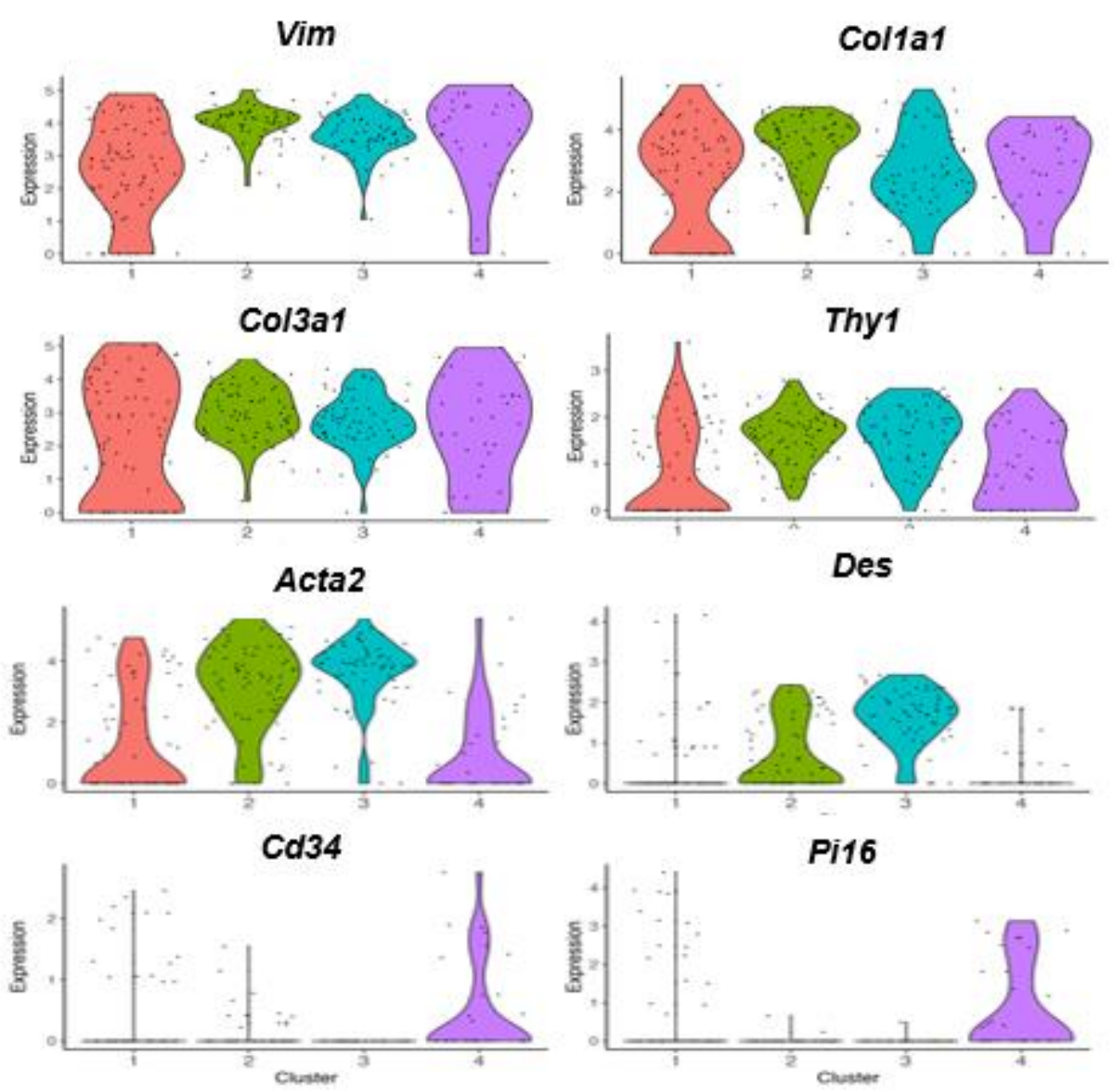

Fig1F,G,I,J: CD34 staining seems to be primarily on blood vessels. This is a major

issue as it may affect the interpretation of all results. The authors should validate these are not endothelial cells/pericytes, by adding markers such as MECA32/CD31+RGS5/MCAM.

Response: Thank you for your suggestion. In tumor sections, CD34 staining is observed both in blood vessels and in the surrounding stroma. To confirm the presence of $CD34^{+}$ fibroblast progenitors and exclude contamination by endothelial or perivascular cells, we performed co-staining for CD34, CD31, RGS5 and Col1a1. We identified $CD34^{+}Col1a1^{+}CD31^{-}/RGS5^{-}$ cells within the tumor tissue, leading us to conclude that a population of CD34+ fibroblasts does indeed exist in the tumor microenvironment（Supplementary Figure3C,D）.

Fig1H: There's very low correlation between the two groups, which does not point to a similarity as the authors suggest.

Response: There may be a misunderstanding. We developed a computational approach to identify transcriptional similarity between cell type clusters in single-cell RNA sequencing (scRNA-seq) data, enabling the generation of biologically plausible lineage hypotheses based on transcriptomic profiles.

It displays the P-values for the correlations: the smaller the P-value, the stronger the correlation. Blue indicates low P-values, whereas red indicates high P-values. Thus, the low P-value for the correlation between S/P cells and fibroblasts signifies a strong association (**Supplementary Figure 2G** in revised manuscript).

This approach enables the identification of transcriptionally similar cell populations that may share common developmental origins or functional characteristics. Clusters that appear closely related in the hierarchical structure suggest potential lineage relationships or shared regulatory programs. The method provides a quantitative framework for generating testable hypotheses about cellular differentiation trajectories and developmental hierarchies based on transcriptomic similarity patterns.

The clustering-based similarity analysis is particularly valuable for:

• Identifying putative progenitor-progeny relationships

• Discovering shared regulatory networks between cell types
• Validating clustering results and identifying potential misclassifications
• Generating hypotheses for lineage tracing experiments
• Understanding the transcriptional landscape of complex tissues

This computational framework provides a robust foundation for exploring cellular relationships in single-cell transcriptome data and generating biologically meaningful hypotheses about cell type evolution and differentiation pathways.

Algorithm Implementation

The method employs a multi-step computational pipeline that leverages principal component analysis (PCA) and hierarchical clustering to quantify inter-cluster relationships:

1. Dimensionality Reduction: The algorithm extracts the first 20 principal components from the PCA reduction of the Seurat object, capturing the major sources of transcriptional variation while reducing computational noise.
2. Cluster Centroid Calculation: For each cell type cluster, we compute the mean expression profile in the PCA space by averaging the principal component scores across all cells within each cluster, generating representative centroids that summarize the transcriptional characteristics of each cell population.
3. Similarity Matrix Construction: Pairwise similarities between cluster centroids are calculated using cosine similarity, which measures the cosine of the angle between two vectors in the high-dimensional PCA space. This metric is particularly suitable for transcriptional data as it captures the direction of gene expression patterns rather than absolute expression levels.
4. Distance-based Clustering: The cosine similarity matrix is converted to a distance matrix (distance = 1 - similarity) and subjected to hierarchical clustering using the McQuitty method, which generates a dendrogram representing the hierarchical relationships between cell type clusters.
5. Visualization: The resulting distance matrix is visualized as a heatmap with hierarchical clustering dendrograms, where darker colors indicate higher similarity (lower distance) between clusters.

Fig1I-J: Para-GC/Mela means normal-adjacent ?

Response: Yes.

Fig1J: What are the different channels in the staining (yellow/pink/red)

Response: The blue fluorescence channel represents DAPI, which stains the nuclei, while the red fluorescence channel represents CD34.

2. Following up on the pericyte issues, in Fig2 the authors write "Although the precise cellular identity of S3 (C3?) remains elusive, we lean towards the view that C3, which expresses pericyte markers, is a subtype of CAFs with a strong resemblance to myCAFs." Why? These seem like clear pericytes, what makes them myCAFs? Especially as C2 seems to have many more myCAF markers (ECM genes). Also, in Supp. Fig6D as the authors used in-situ sequencing to get a better resolution of colocalization of CD34 and other markers of interest it would be highly helpful for their message that they add single channels of each marker or at least some of the main ones to better convey the idea of CD34 CAFs/fibroblasts. The figure as it is now, is unreadable and the markers are hard to distinguish.

Response: We sincerely apologize for the typo—C3 had been incorrectly written as S3; this has now been corrected in the revised manuscript. Single-cell data show that C3 indeed co-expresses both pericyte and CAF markers, making a clear-cut distinction difficult.

The central focus of this study is the differentiation trajectory from $CD34^{+}Pi16^{+}$ fibroblast progenitors into $Acta2^{+}$ CAFs. To determine whether bona-fide pericytes emerge along this path, we cultured sorted $CD34^{+}Pi16^{+}$ progenitors in vitro and exposed them to gastric-cancer cell-conditioned medium or TGF-β. Using Rgs5 as a pericyte marker, we observed no significant change in its expression throughout the differentiation time course, and overall levels remained very low. These results indicate that pericyte differentiation does not occur within this lineage trajectory ,

which was shown in Figure 4 in revised manuscript.

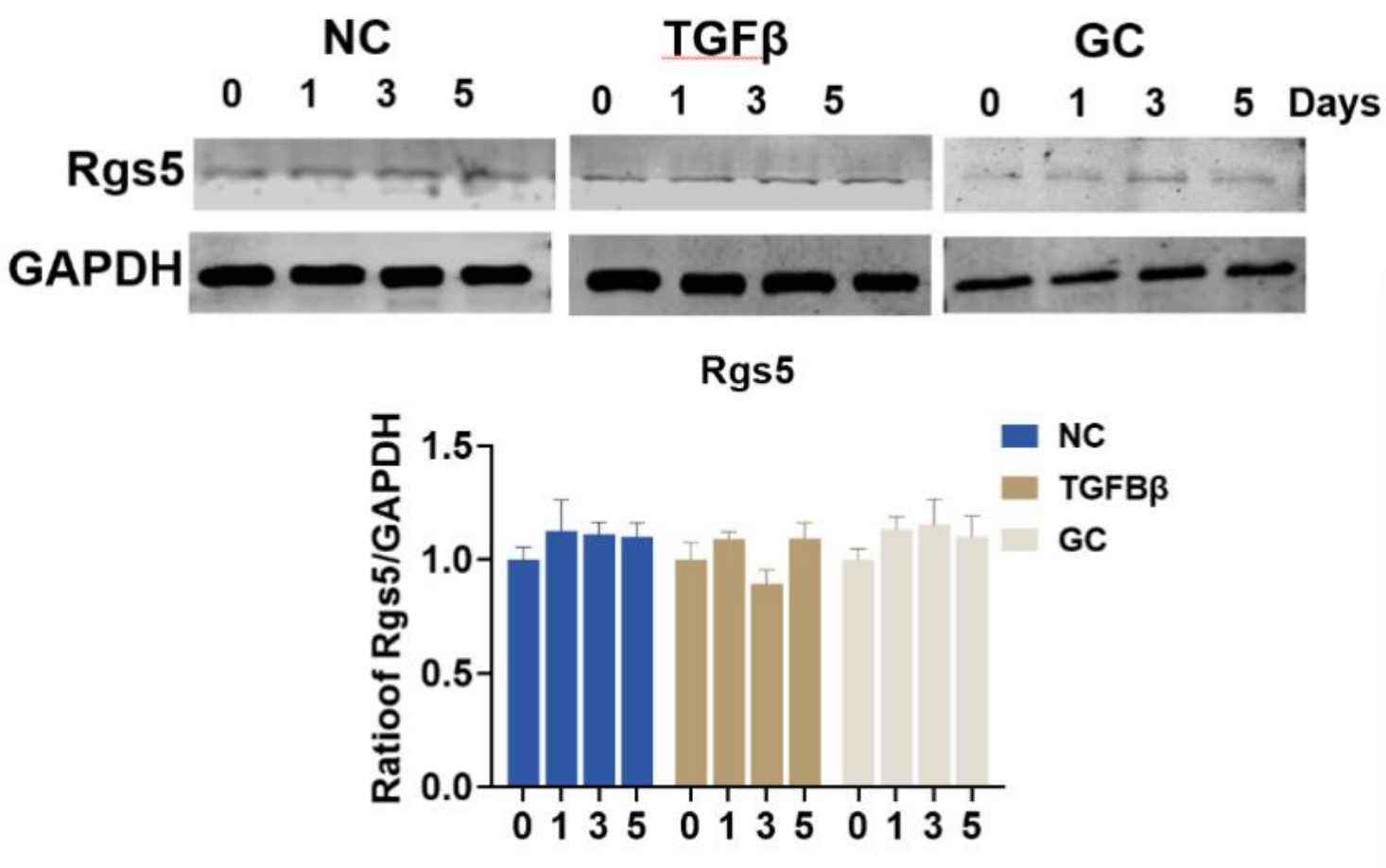

For the in-situ sequencing experiments, technical limitations within our team make it difficult to visually assign each fluorescent channel to its corresponding marker by eye. Instead, we rely on analysis software to calculate inter-marker distances and generate approximate distribution maps. Because this section serves only as a preliminary reference, we will continue to refine our approach; improvements in methodology should yield more definitive conclusions in future work. This part is in the newly revised Supplemental Figure 11.

3. Supp 7, Fig3C-D - The authors write "To validate this phenotypic development, we isolated CD34+ fibroblasts from mouse tumor tissue and cultured them in differentiation medium with B16 culture medium for 14 days. Significant downregulation of CD34 and PI16 but upregulation of α-SMA were observed,..." (supp fig7) and the same assay was performed for gastric CAFs from human tumors. If the researchers used a differentiation medium to mimic the effects of cancer cells, it important to add a control condition where the medium is neutral and should not have an effect. In general, this is a problematic assay to test this specific theory as most fibroblasts grown in 2D for more than a few days will develop a myCAF like phenotype, this should be validated in other forms or in 3D culture.

Response: Thank you for your detailed comments. We isolated $CD34^{+}Pi16^{+}$ fibroblast progenitor cells and subjected them to differentiation in control medium, tumor-cell-conditioned medium, or medium supplemented with TGF-β. Clonogenic

assays, telomere-length measurements, and lineage-specific marker analyses were employed to monitor the differentiation process. Addition of the differentiation inhibitor LIF best preserved stemness, whereas control medium induced only slow differentiation and tumor-cell-conditioned medium markedly accelerated it. The differentiation-related data are presented in **Figure 4** in revised manuscript.

4. Fig3A - The authors performed reanalysis of an existing scRNAseq data set that includes "230,000 fibroblasts across 17 tissues, 50 datasets, 11 disease states and 2 species", yet the analysis presented here includes only ~10,000 cells. Could the authors elaborate in the methods how was the dataset analyzed, which diseases/samples were included, how the cells were filtered etc.

Response: Thank you for your helpful comment and apologize for not providing sufficient methodological details in the initial submission. Our reanalysis was intentionally straightforward and aimed to address a focused question: whether a stromal progenitor (SP)-like population similar to what we observed in our mouse data can also be identified in an external human scRNA-seq resource. To this end, we downloaded the curated human fibroblast Seurat object Human_PS_Fibro.RDS from FibroXplorer (www.fibroxplorer.com/download). This object is a human-only subset provided by the resource and therefore contains 10,355 fibroblasts, rather than the full compendium that includes multiple species (~230,000 cells across 17 tissues, 50 datasets, and 11 disease states). We reused the original unsupervised clustering and low-dimensional embeddings supplied with this Seurat object and did not perform further filtering beyond what was present in the curated dataset. Following the original resource's guidance, we mapped CD34 and PI16 expression onto the provided clusters/UMAPs. Among the six unsupervised clusters in this dataset, cluster 2 showed clear enrichment of both CD34 and PI16. This observation indicates that the SP-like population we define is already apparent within the original unsupervised clustering of the FibroXplorer human subset, thereby independently supporting the conclusion presented in our manuscript.

5. Fig 4 - Generally, the idea behind the experiment presented is to show that depletion of CD34+ linage cells hinders formation of myCAFs later in tumor development and tumor growth in general. The major caveat of this approach is that the depletion of CD34+ cells depletes endothelial cells as well, which are highly important in tumor establishment and growth. The authors should add staining for CD31/MECA32 to show that the main cause of reduced tumor growth is not absence of vascularization . This will help strengthen their theory that the loss of myCAFs is the main cause for tumor reduction.

Response: We appreciated your comments. Indeed, cells of the CD34 lineage are quite heterogeneous. Depleting all CD34-lineage cells would affect a subset of hematopoietic and vascular cells, introducing considerable uncertainty in tumor growth. To specifically target the Cd34+ fibroblast subset, we generated $CD34^{+}Pi16^{+}$ cell double-positive mice and administered diphtheria toxin (DT) to ablate only the $CD34^{+}Pi16^{+}$ population. This strategy markedly reduces off-target effects, limiting toxicity to the fibroblast-progenitor differentiation pathway. After selective ablation, tumor growth was restrained to varying degrees. Interestingly, although the overall number of intratumoral endothelial cells remained unchanged, the vessels that did form were predominantly small-caliber; vessel density increased, yet large, fully structured vessels were scarce (Figure 3I–K).

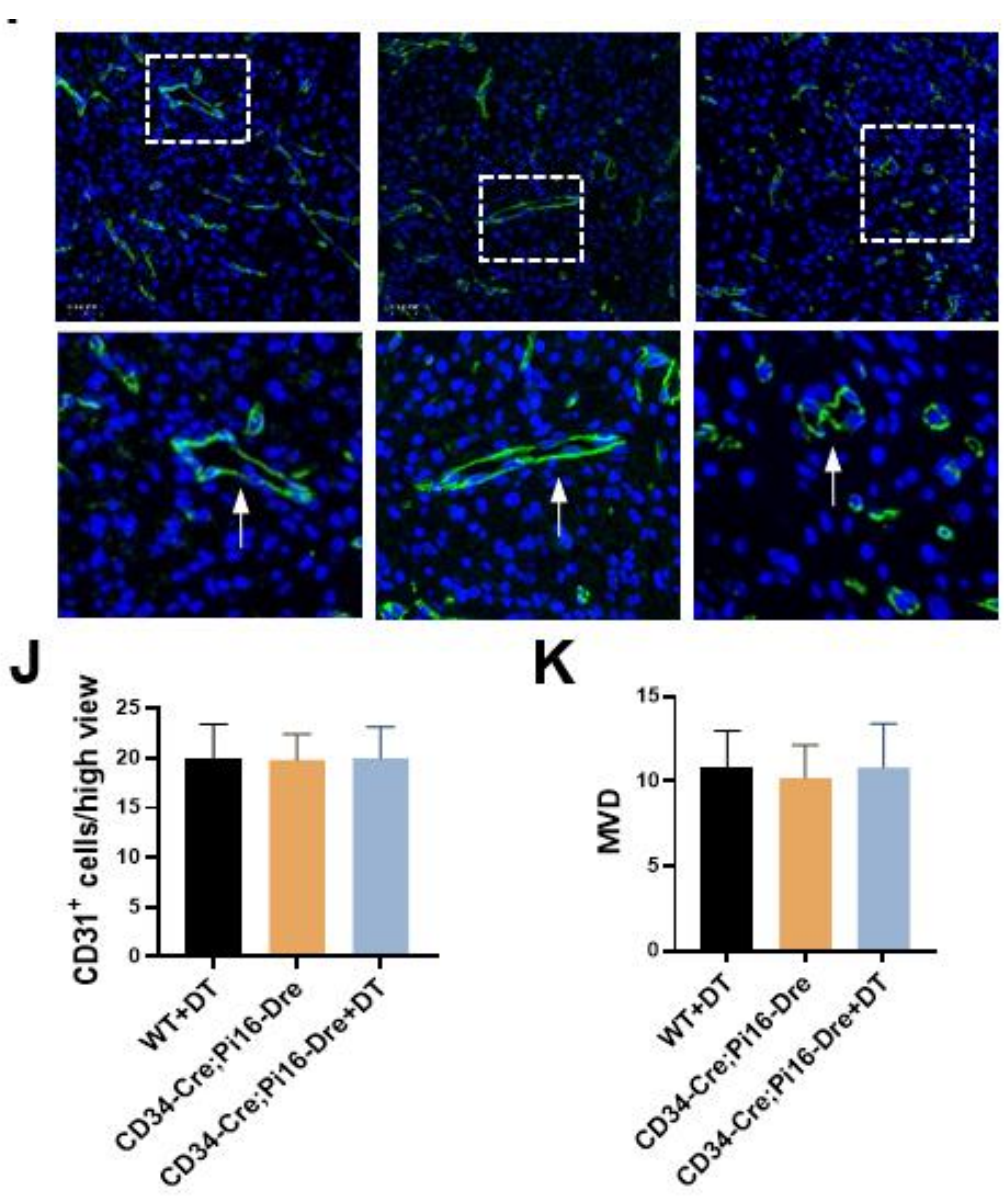

6. Fig5 - Overall the results are very promising, but again the authors should validate

the cause of tumor reduction is not the absence of blood vessels formation, as they mentioned.

Response: We completely agree with the reviewer. To further clarify whether the reduction in tumor size is due to a decrease in blood vessels, we performed CD31 staining on tumor tissues from four drug-treated groups to assess intratumoral endothelial cells and angiogenesis. The results showed no reduction in endothelial cells and no significant difference in microvessel density. In contrast, the positive control group exhibited a significant decrease in microvessel density. Therefore, we conclude that the drugs do not inhibit tumor growth by suppressing angiogenesis (Figure 5E,F).

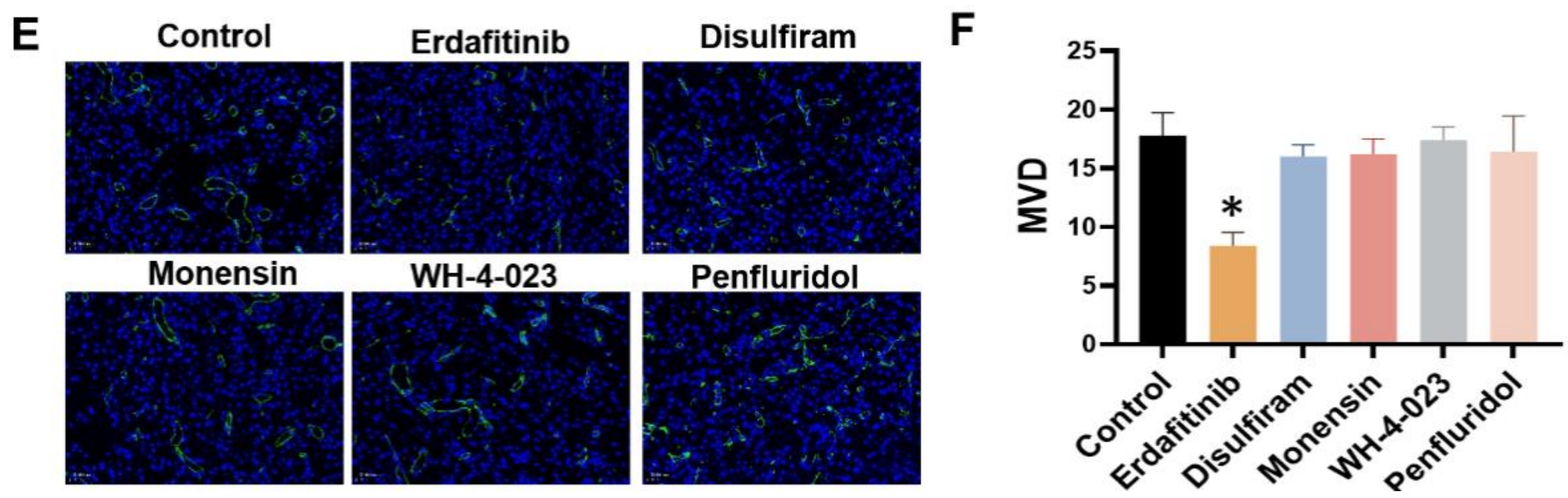

7. Fig6F-G - What is the difference between F and G, it is unclear from the figure legend. Furthermore, some of the graphs show significance but the effect is very minor, how was the significance tested? Also, some of the time points presented don't have error bars, does this mean there was only one sample for this time point?

Response: There may be a misunderstanding. In original Figure 6F (Figure 4H in revised manuscript), we examined the effect of foxs1 knockdown on differentiation in standard differentiation medium, whereas Figure 6G (Figure 4 I in revised manuscript) entiation induced by tumor-conditioned medium; this distinction is now clearly labeled in the revised figure. The data indicate that foxs1 knockdown partially suppresses Acta2 expression and impedes the differentiation process. Each experiment was performed in three independent replicates; the corresponding standard deviations varied, and smaller SDs are less apparent in the graphs. We have revised the figure accordingly.

8. Methods - In the fluorescence staining the authors did not mention how was the MxIF performed (which fluorescent reagents, on which channel) as the tumors express GFP it is important to make sure there is no clash with the staining. If the tumors are not GFP+ in this figure, how was the tumor/stroma areas defined.

Response: Thank you for your detailed comments. We have added the multiplex fluorescence staining protocol to the Methods section. The tumor tissue used for staining was derived from non-fluorescently labeled cells; tumor areas were identified by histopathological morphology, and the subcutaneous xenograft model consisted almost entirely of tumor tissue.

9. Generally, it is not clear from the methods section or figure legends how many times each experiment was repeated.

Response: Thank you for your inquiry. All experimental results were obtained from three independent replicates and are presented as mean ± SD. We have added this statement to the Methods section of the manuscript.

Minor:

Linguistic editing is required throughout the manuscript.

Response: Thanks a lot. We have completed the Linguistic editing of the manuscript.

Fig2B: What are the units in the y axis?

Response: Thank you for your detailed comments. I apologize for not labeling it clearly. Fig. 2B is a UMAP dimensionality reduction plot; the X and Y axes represent UMAP_1 and UMAP_2, respectively.

Supp Fig2B: y axis - Ratio is from 0 to 1, please change to percent.

Response: Thank you for your suggestion. We have made the necessary revisions.

Fig2G - In page 7 the authors write- "we intuitively regrouped them into 3 dominant subpopulations..." Intuitively is not an analytical method for clustering please write the method used for clustering the cells (Louvain or other..).

Response: We thank the reviewer for the helpful suggestion and apologize for our imprecise wording. The fibroblast subpopulations were defined using Seurat's standard graph-based, unsupervised clustering workflow: PCA for dimensionality reduction, construction of a shared nearest neighbor (SNN) graph with FindNeighbors, and community detection with FindClusters (Louvain algorithm, default parameters). After obtaining the initial unsupervised clusters, we consolidated them into three dominant subpopulations based on canonical marker expression and transcriptional similarity, not by ad hoc intuition. We will revise the text accordingly to replace "intuitively" with the explicit description of this clustering procedure.

We would like to once again thank both editors and reviewers for his/her careful reading of our paper, and for the helpful comments and suggestions. We hope that our manuscript could be satisfactory and appropriate for publication in *EMBO molecular medicine*

Yours sincerely

LI ZHANG

Li Zhang, MD, PhD

5th Dec 2025

Dear Prof. Zhang,

Thank you for your appeal on our previous decision and for submitting a revised manuscript to EMBO Molecular Medicine. Following editorial evaluation of the revisions, we sent the manuscript back to the initial referees.
As you will see below, while referees #1 and #3 support publication pending minor revisions (but still mention several remaining concerns), referee #2 raises a major issue related to the lack of a demonstrated role for Foxs1. Given the potential interest of the findings and after further consultation with the referees, we would like to invite you to further revise the manuscript to address this and the other remaining issues.

As EMBO Press usually only allows one round of revisions, please be aware that this will be your last opportunity to address these issues. The revised manuscript will be reviewed again, and we cannot guarantee a positive outcome at this stage.

Should you find that the requested revisions are not feasible within the constraints outlined here and prefer, therefore, to submit your paper elsewhere, we would welcome a message to this effect.

I look forward to seeing a revised form of your manuscript as soon as possible. Use this link to login to the manuscript system and submit your revision: https://embomolmed.msubmit.net/cgi-bin/main.plex

Yours sincerely,

Lise Roth

Lise Roth, Ph.D
Senior Editor
EMBO Molecular Medicine

When preparing your revised manuscript, please refer to our guidelines: https://link.springer.com/journal/44321/submission-guidelines#cms-Revised-submissions. We perform an initial quality control of all revised manuscripts before re-review; failure to include requested items will delay the evaluation of your revision.

We require:

1) A .docx formatted version of the manuscript text (including legends for main figures, EV figures and tables). Please make sure that the changes are highlighted to be clearly visible.

2) Individual production quality figure files as .eps, .tif, .jpg (one file per figure). For guidance, download the 'Figure Guide PDF': https://media.springernature.com/original/springer-cms/rest/v1/content/27825798/data/v1.

3) A .docx formatted letter INCLUDING the reviewers' reports and your detailed point-by-point responses to their comments. As part of the EMBO Press transparent editorial process, the point-by-point response is part of the Review Process File (RPF), which will be published alongside your paper.

4) A complete author checklist, which you can download from our author guidelines. Please insert information in the checklist that is also reflected in the manuscript. The completed author checklist will also be part of the RPF.

5) Please note that all corresponding authors are required to supply an ORCID ID for their name upon submission of a revised manuscript.

6) It is mandatory to include a 'Data Availability' section after the Materials and Methods. Before submitting your revision, primary datasets produced in this study need to be deposited in an appropriate public database, and the accession numbers and database listed under 'Data Availability'. Please remember to provide a reviewer password if the datasets are not yet public.

In case you have no data that requires deposition in a public database, please state so in this section. Note that the Data Availability Section is restricted to new primary data that are part of this study.

7) For data quantification: please specify the name of the statistical test used to generate error bars and P values, the number

(n) of independent experiments (specify technical or biological replicates) underlying each data point and the test used to calculate p-values in each figure legend. The figure legends should contain a basic description of n, P and the test applied. Graphs must include a description of the bars and the error bars (s.d., s.e.m.).

8) At EMBO Press we ask authors to provide source data for the main manuscript figures. You will receive a separate email with instructions for providing source data with your revised manuscript, including how to upload and organize the files.

9) Our journal encourages inclusion of *data citations in the reference list* to directly cite datasets that were re-used and obtained from public databases. Data citations in the article text are distinct from normal bibliographical citations and should directly link to the database records from which the data can be accessed. In the main text, data citations are formatted as follows: "Data ref: Smith et al, 2001" or "Data ref: NCBI Sequence Read Archive PRJNA342805, 2017". In the Reference list, data citations must be labeled with "[DATASET]". A data reference must provide the database name, accession number/identifiers and a resolvable link to the landing page from which the data can be accessed at the end of the reference.

10) We replaced Supplementary Information with Expanded View (EV) Figures and Tables that are collapsible/expandable online. A maximum of 5 EV Figures can be typeset. EV Figures should be cited as 'Figure EV1, Figure EV2" etc... in the text and their respective legends should be included in the main text after the legends of regular figures.

- For the figures that you do NOT wish to display as Expanded View figures, they should be bundled together with their legends in a single PDF file called *Appendix*, which should start with a short Table of Content. Appendix figures should be referred to in the main text as: "Appendix Figure S1, Appendix Figure S2" etc.

- Additional Tables/Datasets should be labeled and referred to as Table EV1, Dataset EV1, etc. Legends have to be provided in a separate tab in case of .xls files. Alternatively, the legend can be supplied as a separate text file (README) and zipped together with the Table/Dataset file.

11) The paper explained: EMBO Molecular Medicine articles are accompanied by a summary of the articles to emphasize the major findings in the paper and their medical implications for the non-specialist reader. Please provide a draft summary of your article highlighting
- the medical issue you are addressing,
- the results obtained and
- their clinical impact.

This may be edited to ensure that readers understand the significance and context of the research. Please refer to any of our published articles for an example.

12) Author contributions: You will be asked to provide CRediT (Contributor Role Taxonomy) terms in the submission system. These replace a narrative author contribution section in the manuscript.

13) A Conflict of Interest statement should be provided in the main text.

14) Every published paper includes a 'Synopsis' to further enhance discoverability. Synopses are displayed on the journal webpage and are freely accessible to all readers. They include a short stand first (maximum of 300 characters, including space) as well as 2-5 one-sentences bullet points that summarizes the paper. Please write the bullet points to summarize the key NEW findings. They should be designed to be complementary to the abstract - i.e. not repeat the same text. We encourage inclusion of key acronyms and quantitative information (maximum of 30 words / bullet point). Please use the passive voice. Please attach these in a separate file or send them by email, we will incorporate them accordingly.

Please also suggest a striking image or visual abstract to illustrate your article as a PNG file 550 px wide x 300-600 px high.

15) Include a Reagents and Tools Table as part of the Methods section, which can be downloaded from our author guidelines.

EMBO Molecular Medicine has a "scooping protection" policy, whereby similar findings that are published by others during review or revision are not a criterion for rejection. Should you decide to submit a revised version, I do ask that you get in touch after three months if you have not completed it, to update us on the status.

***** Reviewer's comments *****

Referee #1 (Comments on Novelty/Model System for Author):

Authors used advanced methodologies to study a timely, clinically pertinent, question.

Referee #1 (Remarks for Author):

Interesting paper, but a bit overwhelming (especially after the addition of substantial new data). Some specific comments below:

The title implies that drug screening contributed to the identification of Foxs1. I do not think this is correct.

In Figure 1A, the S/P population is hard to find.

Figure 1L (callout says "I"); can the authors demonstrate that cKit is S/P cell specific, given its known expression on cancer cells.

A similar question relates to Figure 2J, where "Cd34-lineage-traced tumors" are mentioned. I understood the host is Cd34-labeled?

Figure 4; I do not see why Foxs1 is such a likely TF to choose. The association of several other TFs seems stronger.

Related to that; how where the 4 compounds shown shortlisted from the 40 initial hits?

Note that the MoneNsin typo is still in Figure 5B.

The purpose of quantifying MVD in Figure 5E,F is not clear to me.

The description of Figure 5G,H in the Results section is hard to follow. It is not clear which markers would be expected to stay expressed or decreased.

Figure 6A is hard to interpret.

It would be helpful to perform scRNA-Seq on a selected compound as shown in Figure 6 to establish more in-depth proof of the phenotype obtained. Although markers are shown, a comprehensive comparison and statistical test to establish whether the drugs indeed revert the phenotype would strengthen the study.

Referee #2 (Remarks for Author):

The manuscript has been improved, however, there are still significant defects by the following.

1. Whether transition of CD34+Pi16+fibro to myCAFs mediates tumor growth through foxs1 induction in vivo remains unclear. To address this question, the author would mate CD34/Pi16-Cre ERT2/Rosa26-LSL-tdTomato mice with foxs1 flox/flox mice, prior to tumor injection. Alternatively, the author could implant tumor cells with CD34+fibroblsts expressing foxs1-shRNA or control shRNA into recipient mice. Tumor growth and transition of CD34+Pi16+fibro to myCAFs should be then evaluated.
2. Drug screening data in Fig. 5 and 6 looks related but independent of Fig. 1-4.
Why foxs1expression is not shown in Fig 5? There is no evidence that foxs1 inhibition by the drug treatment mediates the attenuated transition of CD34+Pi16+fibro to myCAFs and reduced tumor growth.
3. In Fig 3J, K, vascular lumen volume data are not shown, although the author claimed in text that vascular lumen volume was altered in the DT-treated mice. The data should also be included in the paper.

Referee #3 (Remarks for Author):

This revised version of the manuscript more accurately reflects the interpretation presented by Yang et al. The added analysis of human scRNA-seq data, together with the use of the dual Cre-Dre mouse model for lineage-specific ablation, substantially strengthens the evidence that this Pi16+ Cd34+ mesenchymal stem cell population contributes to the emergence of pro-tumorigenic Acta2^{+} CAFs specifically myCAF/ECM-producing CAFs and suggests that targeting their transformation may be therapeutically beneficial. The comprehensive set of in vitro and in vivo experiments, incorporating a genetic mouse model and multiple drug candidates targeting Foxs1, as well as the demonstrated loss of Acta2^{+} CAFs and associated tumor reduction, collectively provide a compelling mechanistic framework for pathogenic CAF transformation driven by cancer-cell signaling and Foxs1 activation.
Overall, the manuscript now presents a more thorough investigation and more effectively rules out alternative explanations involving other CD34^{+} cell types within the TME. Using multiple complementary tools, the authors convincingly show that mesenchymal cells/CAFs underline the pro-tumorigenic mechanism described.
Minor comments

• Figures 4C and 7E: Please ensure uniform spacing between image tiles to improve readability.
• Repeated measures analysis: The statistical method used for repeated-measures experiments (e.g., Figures 4H/I and 5C) is not specified, and some of the reported significant differences appear unlikely across the full time course. If only certain time points are significant, please indicate this by placing asterisks above the relevant points and specify the statistical test used.
• Figure 5C: The color scheme for the drug treatments does not match that used in other related figures (5D/F). There also appears to be inconsistency in drug naming (e.g., Erdafitinib in 5C vs. Erdafitiram in 5D). Additionally, the growth curve for the Erdafitinib treatment does not align with the tumor weight shown for the Erdafitiram group. If these represent different experiments, please clarify in the text; if they represent the same experiment, please correct the inconsistencies.
• Figures 5C-D and Supplementary Figure 10B: These are the only instances where "Penfluride" appears, which I assume is meant to be penfluridol. Please correct if so.
• Figure 7F - please match the colors of the different drugs to previous charts

RE: EMM-2024-21040：Foxs1-mediated transformation of CD34+ fibroblast to myCAFs promotes tumor growth

Dear Editors,

We thank the Editors and the Reviewers for their valuable time and constructive comments on our manuscript entitled "Foxs1-mediated transformation of CD34+ fibroblast to myCAFs promotes tumor growth". We have carefully considered all the comments and have incorporated corresponding revisions into the manuscript, with all changes highlighted in the manuscript. The major revisions are as follows:

1.We performed single-cell RNA sequencing on tumor tissues treated with WH-4-023 and control groups, confirming that the drug inhibits the transition from CD34+ fibroblasts to Acta2+ CAFs and suppresses tumor growth.

2.We implanted CD34+ fibroblasts with Foxs1 knockdown and melanoma cells at a 1:3 ratio into the subcutaneous tissue of mice, and found that Foxs1 inhibition reduces the number of Acta2+ CAFs and suppresses tumor growth.

3.We confirmed that all four candidate drugs can inhibit Foxs1 expression.

Below, we provide a point-by-point response to each of the suggestions raised.

Referee 1 (Comments on Novelty/Model System for Author):

Authors used advanced methodologies to study a timely, clinically pertinent, question.

Referee 1 (Remarks for Author):

Interesting paper, but a bit overwhelming (especially after the addition of substantial new data). Some specific comments below:

Response: We appreciate the positive comments from the reviewer and revised the manuscript according to the specific comments.

1. The title implies that drug screening contributed to the identification of Foxs1. I do not think this is correct.

Response: Thank you for the comment. The title has been changed to: "Foxs1-mediated transformation of CD34+ fibroblast to myCAFs promotes tumor growth".

2. In Figure 1A, the S/P population is hard to find.

Response: We understand the question well. Due to the small number of S/P cells, they appeared as a small cluster in the image located next to the fibroblasts, which has been circled in red.

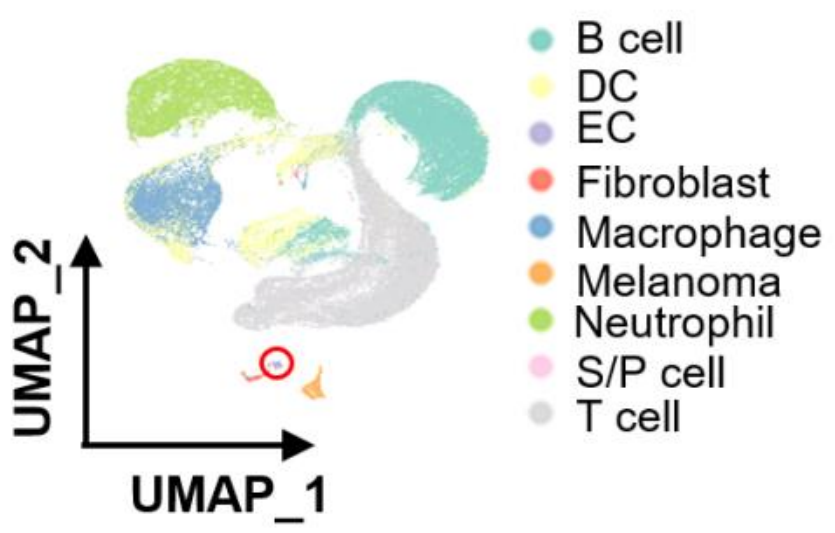

3. Figure 1L (callout says "I"); can the authors demonstrate that cKit is S/P cell specific, given its known expression on cancer cells.

Response: Thank you for the reviewer's question. Figure 1L has been corrected and is now highlighted. In addition, c-Kit is indeed expressed in melanoma and gastric cancer cells, but its expression level shows some heterogeneity in clinical samples. Consequently, it is impossible to determine the enrichment of c-Kit-positive S/P cell in tumor versus non-tumor tissues. Therefore, we have removed all c-Kit-related content from the revised manuscript.

4. A similar question relates to Figure 2J, where "Cd34-lineage-traced tumors" are mentioned. I understood the host is Cd34-labeled?

Response: The reviewer's comment is correct. The term "Cd34-lineage–traced tumor" was inaccurate; we indeed meant "Cd34-labeled." We have corrected this in the revised manuscript to: "In the subcutaneous tumor tissue of CD34-labeled mice".

5. Figure 4; I do not see why Foxs1 is such a likely TF to choose. The association of several other TFs seems stronger.

Response: We thank the reviewer for this important question regarding our rationale for selecting Foxs1. In our investigation of CD34+ fibroblast-to-myofibroblast (myCAF) differentiation, we employed a systematic screening and validation pipeline to select Foxs1 from 16 candidate transcription factors for comprehensive mechanistic characterization.

Based on pseudotime analysis, we identified 9 transcription factors whose expression trends aligned with the progression of changes. We then examined their expression at 0h, 12h, 24h, and 48h following stimulation with tumor-conditioned medium. qPCR results showed that Ets1, Foxs1, Nr2f1, Nr2f2, Tbx2, and Id1 were upregulated at 24h post-stimulation, with Id1 exhibiting significant elevation as early as 12h. Notably, Foxs1 displayed the most pronounced upregulation at the 24h timepoint, which showed highly coordinated expression dynamics with Acta2. The results are shown in S8.

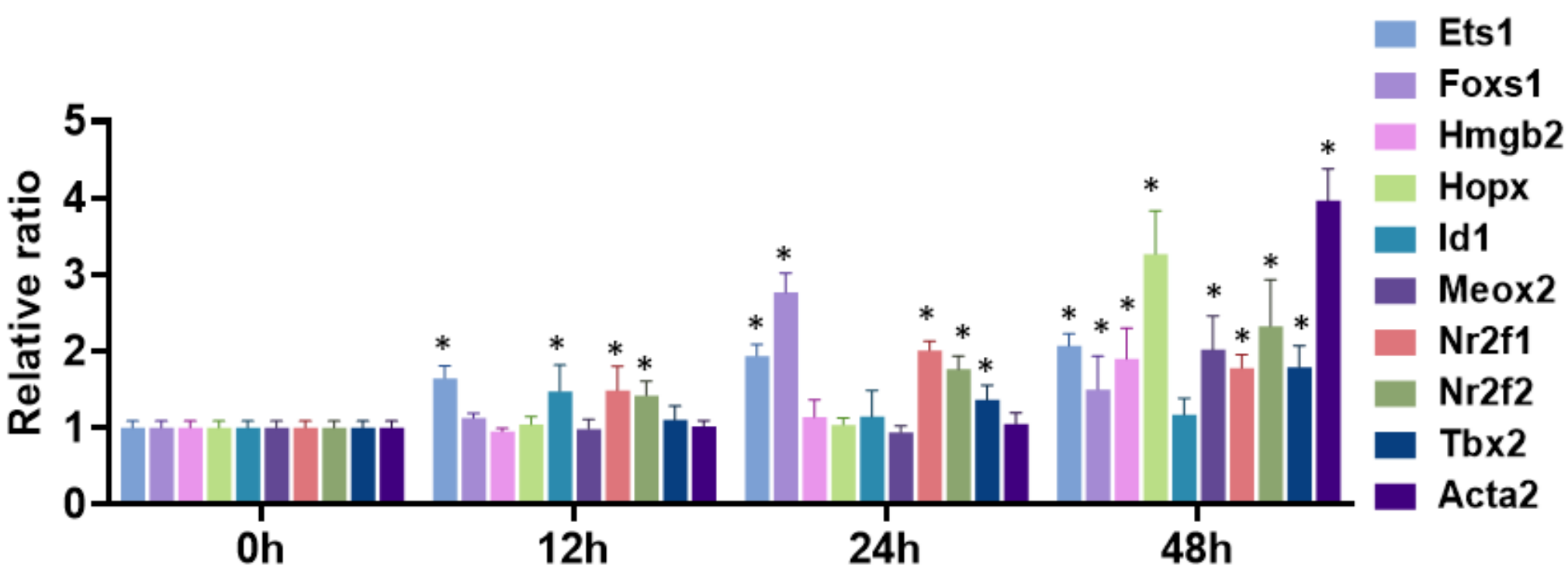

Preliminary experimental results showed that knockdown of these transcription factors led to varying degrees of reduction in Acta2 expression. The knockdown of foxs1 had the greatest impact on Acta2 expression.

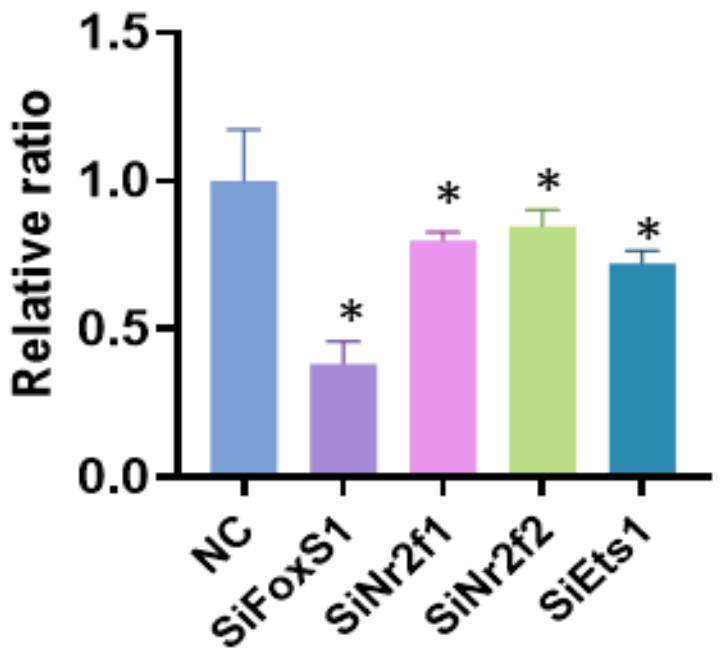

Additionally, two independent studies have also identified and confirmed the critical role of Foxs1 in the fibrotic process.

References:

1. Bates EA et al., J Biol Chem 2024 (PMID: 38280429): Identified FOXS1 as a regulator of TGF-b responsiveness in hepatic stellate cells during liver fibrosis**.**

2. Melchionna R et al., J Exp Clin Cancer Res 2023 (PMID: 38124183): Recognized FOXS1 as a key factor in fibroblast plasticity and CAF heterogeneity. Forms a transcription factor network with TCF4, SOX9, and EGR2 controlling myofibroblast differentiation.

6. Related to that; how where the 4 compounds shown shortlisted from the 40 initial hits?

Response: We thank the reviewer for this important question regarding our selection of four drug candidates from the initial pool of 40 compounds. We would like to clarify the objectives of our study and the rationale behind our drug selection.

It is important to emphasize that the primary goals of this study were to: (1) elucidate the role of the $Cd34^+$ to $Acta2^+$ CAF differentiation pathway in tumor progression, and (2) validate the feasibility of signature matching-based computational screening as a method to identify inhibitors of this differentiation process. This represents a proof-of-concept study demonstrating that targeting CAF lineage development is a viable anti-tumor strategy, rather than a comprehensive drug optimization effort.

Candidate Selection Rationale

We acknowledge that all top 20 candidates from each database represent reasonable choices worthy of further investigation for their potential to modulate this differentiation pathway. We do not exclude the potential value of other top-ranked compounds. Our selection of four candidates for in-depth validation was guided by the following practical considerations:

1. Experimental feasibility and resource constraints: Comprehensive validation of each candidate requires extensive in vitro and in vivo experiments spanning 2-3 months per compound. Given limited resources, we selected four representative candidates for rigorous validation.

2. Compound accessibility: We prioritized commercially available compounds with established clinical use or FDA approval, rather than research-grade compounds with

restricted access (e.g., BRD-, SA-, I- series), to ensure experimental reproducibility and facilitate potential clinical translation.

3. Database representation: To minimize technical bias, we selected two candidates from each database (CMap: Disulfiram #7, Monensin #12; LINCS L1000: Penfluridol #4, WH-4023 #14), demonstrating a ranking-based constraint rather than cherry-picking the four highest scores overall.

Validation Results and Significance

Importantly, all four selected candidates demonstrated significant inhibition of CAF differentiation and tumor suppression in both in vitro and in vivo experiments (Figures 5-6). This 100% success rate (4/4) strongly validates the effectiveness of our signature matching-based screening approach, supporting our core conclusions that: (1) the $Cd34^{+}$ to $Acta2^{+}$ CAF transition represents a druggable target, and (2) signature matching can reliably identify inhibitors of this process.

The emphasis of this work remains on demonstrating the biological significance of this differentiation pathway in tumor growth and validating the signature matching methodology as a viable approach for identifying pathway inhibitors. The drug selection served to assess the feasibility of the overall technical pipeline rather than to identify optimal therapeutic agents. We report these findings as proof-of-concept validation. In the revised manuscript, we have provided the complete list of top 20 candidates with their signature-match scores in Supplementary Figures 9A and 9B to facilitate future investigations by our group and others in the field.

7. Note that the MoneNsin typo is still in Figure 5B.

Response: Thank you for your carefulness. We have revised it in the figure.

8. The purpose of quantifying MVD in Figure 5E,F is not clear to me.

Response: MVD assessment was performed to clarify whether the drug’s inhibitory effect is achieved by reducing angiogenesis. This was also raised by another reviewer, so we conducted MVD analysis to further elucidate the mechanism underlying the drug’s inhibitory action, which has been included in the manuscript.

9. The description of Figure 5G,H in the Results section is hard to follow. It is not clear which markers would be expected to stay expressed or decreased.

Response: We appreciate the reviewer's comments. All four drugs maintained CD34 expression while inhibiting a-SMA expression in vivo. We have revised it in the manuscript accordingly and highlighted the changes.

10. Figure 6A is hard to interpret.

Response: We apologize for the confusion. Figures 6A and 6B demonstrate the induction of Cd34+ CAF differentiation into Acta2+ CAF by conditioned medium from gastric cancer cell line MKN. The four drugs were tested for their inhibitory effects on this process.

Figure 6A shows the expression of CD34, Pi16, a-SMA, SPARC, and Col1A1 after 7 days of induction. The results indicate that all four drugs inhibited a-SMA expression. Notably, WH-4-023 increased CD34 expression, while monensin and WH-4-023 upregulated Pi16 expression.

Figure 6B presents the results after 14 days of treatment. The control groups included: pre-differentiation, spontaneous differentiation for 7 days, and tumor-conditioned medium-induced differentiation for 7 days.

Collectively, these results demonstrate that all four drugs can inhibit, to varying degrees, the gastric cancer cell medium-induced differentiation of Cd34+ CAF into Acta2+ CAF.

11.It would be helpful to perform scRNA-Seq on a selected compound as shown in Figure 6 to establish more in-depth proof of the phenotype obtained. Although markers are shown, a comprehensive comparison and statistical test to establish whether the drugs indeed revert the phenotype would strengthen the study.

Response: It is an constructive comment. As the reviewer's suggestion, we have performed additional experiment: We selected WH-4-023 as a representative compound and examined the cellular profiles of CD34+ cell-derived (Tomato+ cell) lineages in tumors following drug treatment. CD34+ cell lineage-tracing mice were induced with tamoxifen for 2 weeks, followed by subcutaneous implantation of melanoma. Prominent tumor formation was observed 1 week later. The tumors were then treated with WH-4-023, and after 1 week, the tumors were harvested. Tomato+ cells were sorted by flow cytometry for single-cell sequencing. We specifically focused on examining the changes and proportions of CD34+ CAFs and Acta2+ CAFs. The results showed that WH-4-023 treatment significantly increased the proportion of CD34+ CAFs while decreasing the proportion of Acta2+ CAFs, confirming our conclusions. This result has been included in the revised manuscript (Figure EV3).

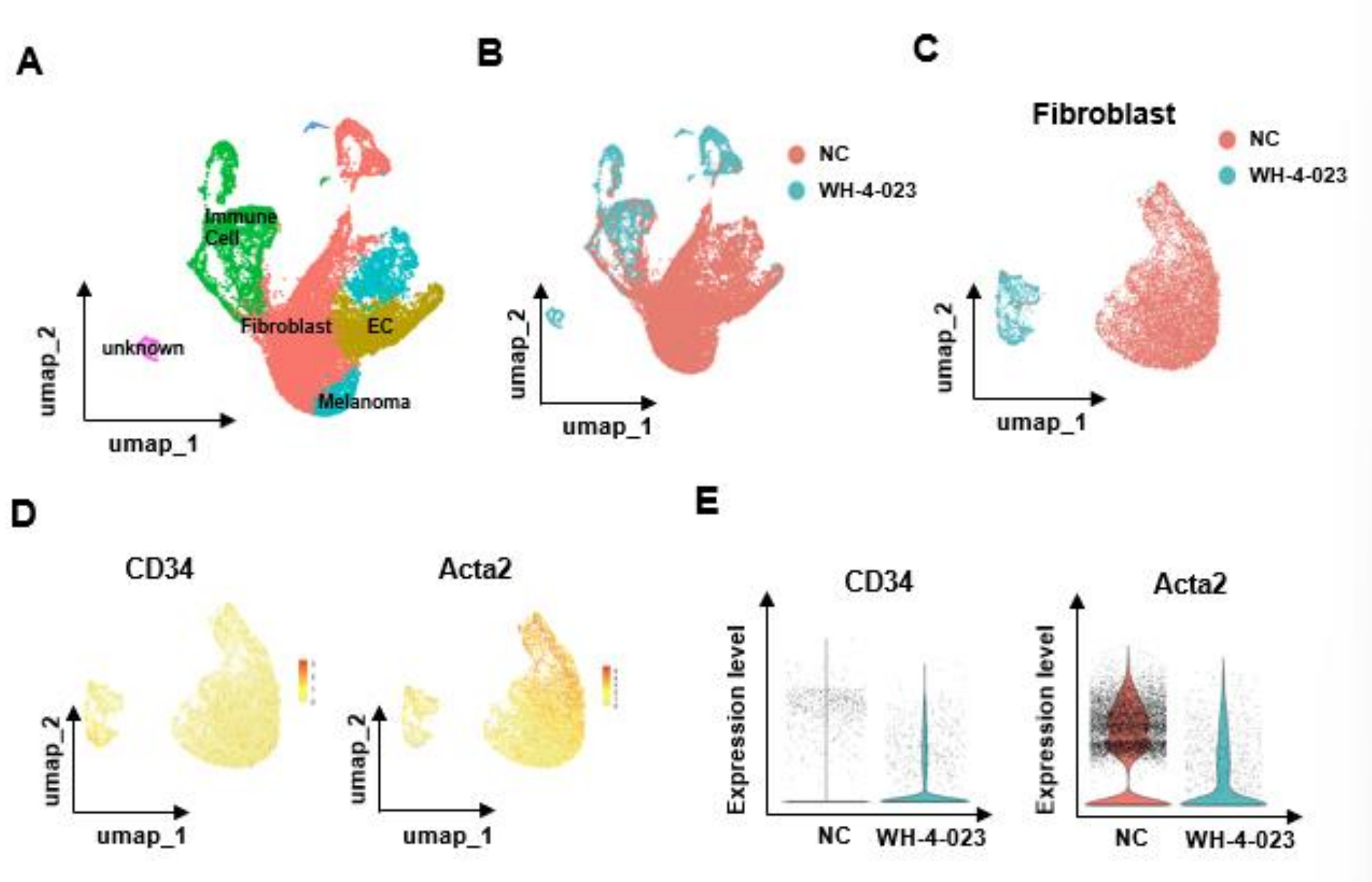

Referee 2 (Remarks for Author):

The manuscript has been improved, however, there are still significant defects by the following.

Response: We appreciate the positive comments from the reviewer and revised the manuscript according to the specific comments.

1. Whether transition of CD34+Pi16+fibro to myCAFs mediates tumor growth through foxs1 induction in vivo remains unclear. To address this question, the author would mate CD34/Pi16-Cre ERT2/Rosa26-LSL-tdTomato mice with foxs1 flox/flox mice, prior to tumor injection. Alternatively, the author could implant tumor cells with CD34+fibroblsts expressing foxs1-shRNA or control shRNA into recipient mice. Tumor growth and transition of CD34+Pi16+fibro to myCAFs should be then evaluated.

Response: Thank you very much for your valuable comments. Following your suggestions, we subcutaneously inoculated CD34+ fibroblasts into mice. The results showed that in the foxs1-shRNA group, tumor growth was slowed and the volume was significantly reduced. In tumor tissues, CD34 and pi16 expression were upregulated, while α-SMA expression was significantly decreased. These findings indicate that knockdown of foxs1 significantly inhibits tumor growth and the transition of CD34+ CAFs to α-SMA+ CAFs. The relevant results are presented in EV1.

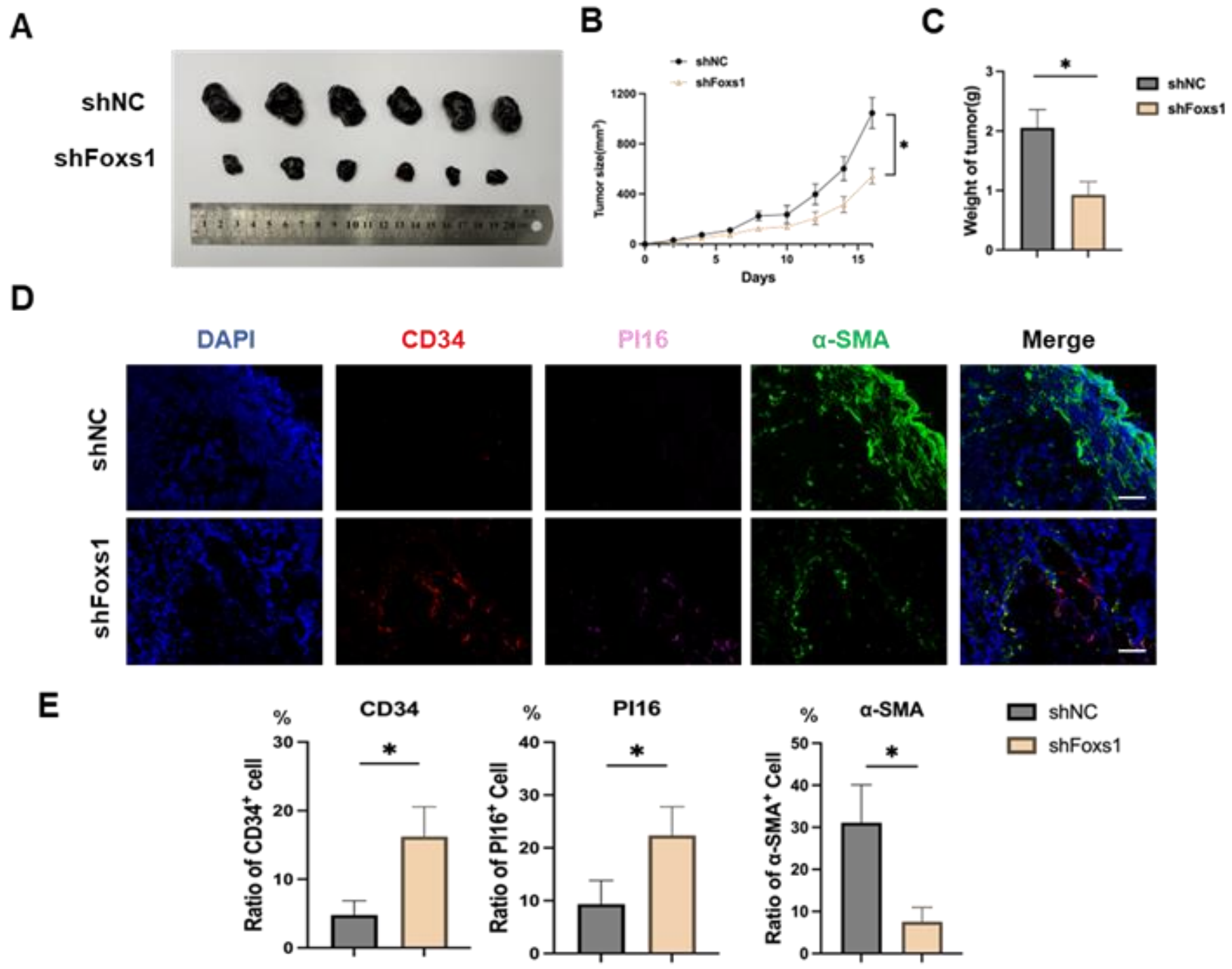

2. Drug screening data in Fig. 5 and 6 looks related but independent of Fig. 1-4. Why foxs1expression is not shown in Fig 5? There is no evidence that foxs1 inhibition by the drug treatment mediates the attenuated transition of CD34+Pi16+fibro to myCAFs and reduced tumor growth.

Response: Thank you for the reviewers'comments. The relationship between the drug and Foxs1 was indeed unclear. We have now examined Foxs1 expression at both the mRNA and protein levels after drug treatment and found that the drug partially suppresses the tumor-conditioned-medium-induced up-regulation of Foxs1. This result has been included in the revised manuscript (Figure EV2C and Figure6 C- E.).

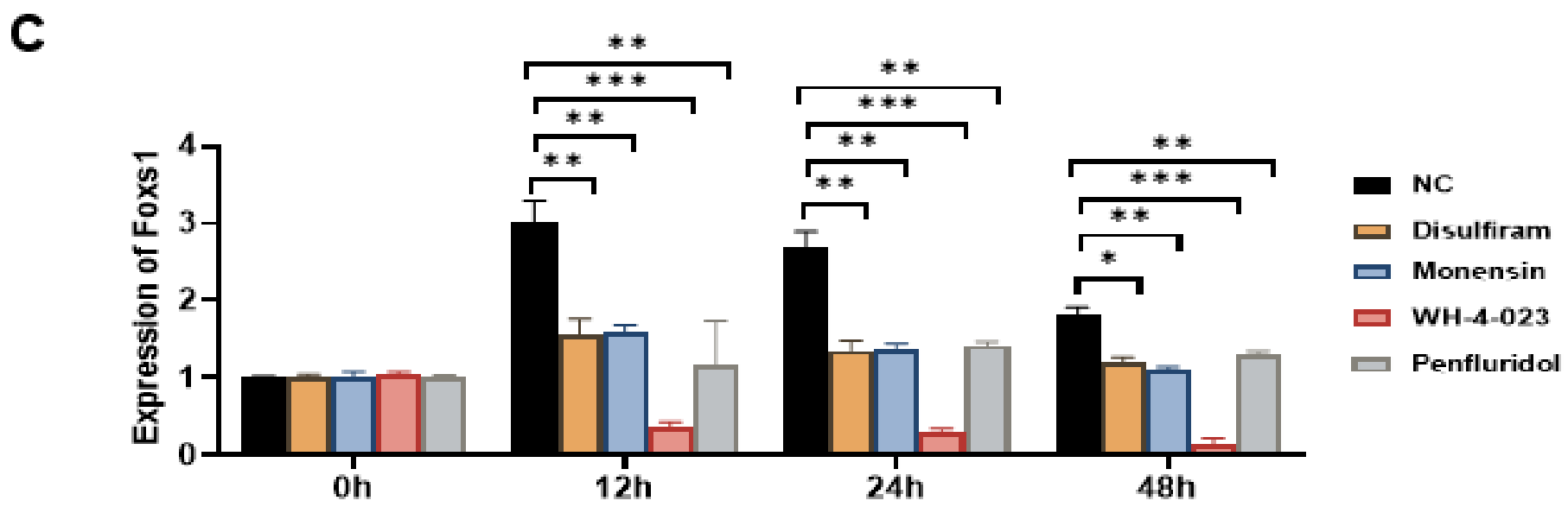

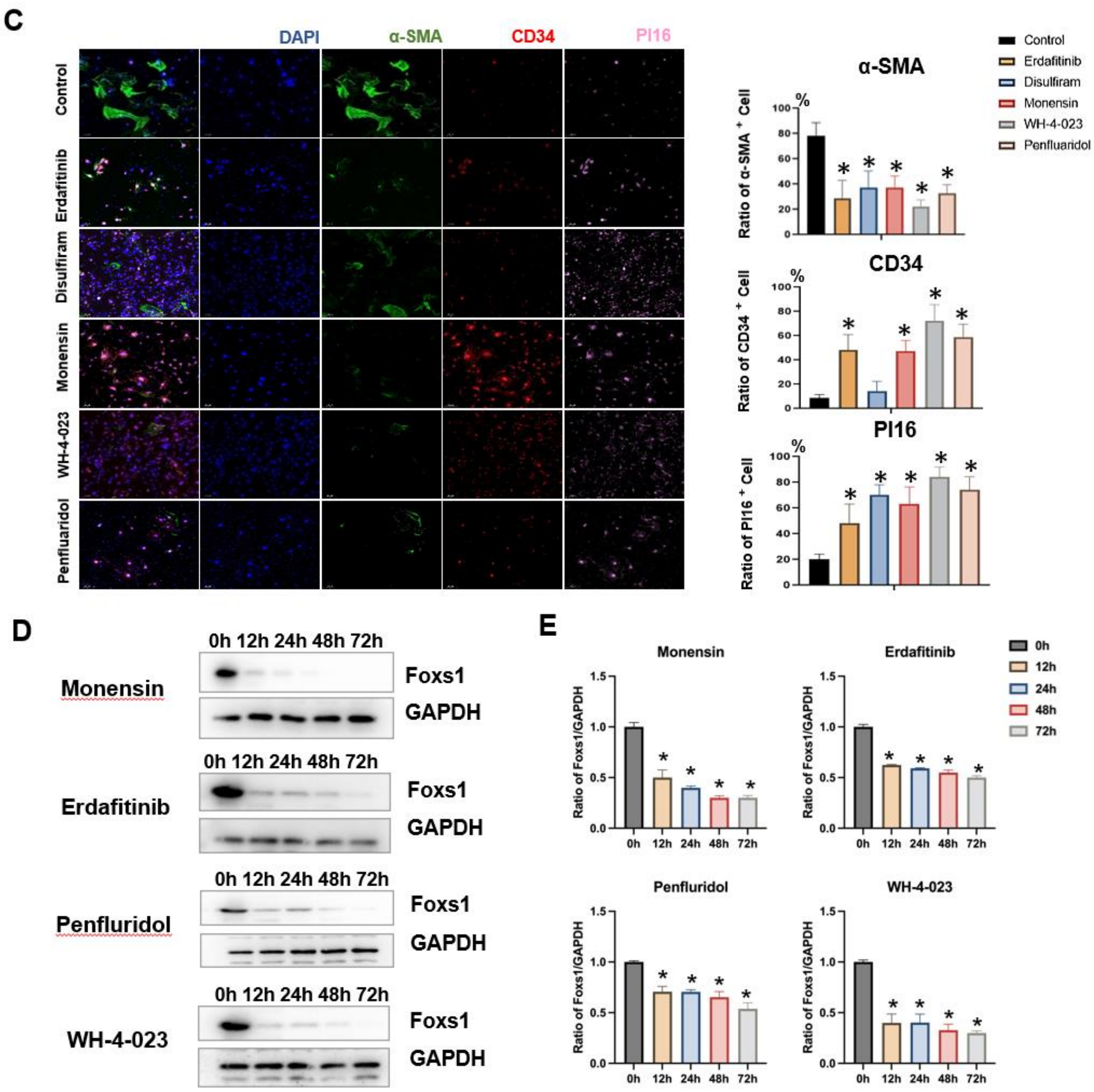

3. In Fig 3J, K, vascular lumen volume data are not shown, although the author claimed in text that vascular lumen volume was altered in the DT-treated mice. The data should also be included in the paper.

Response: We apologize for the omission of statistical analysis in the original submission. In the revised manuscript, we have now performed statistical comparisons of the luminal diameters, and the results are presented in Figure 3L.

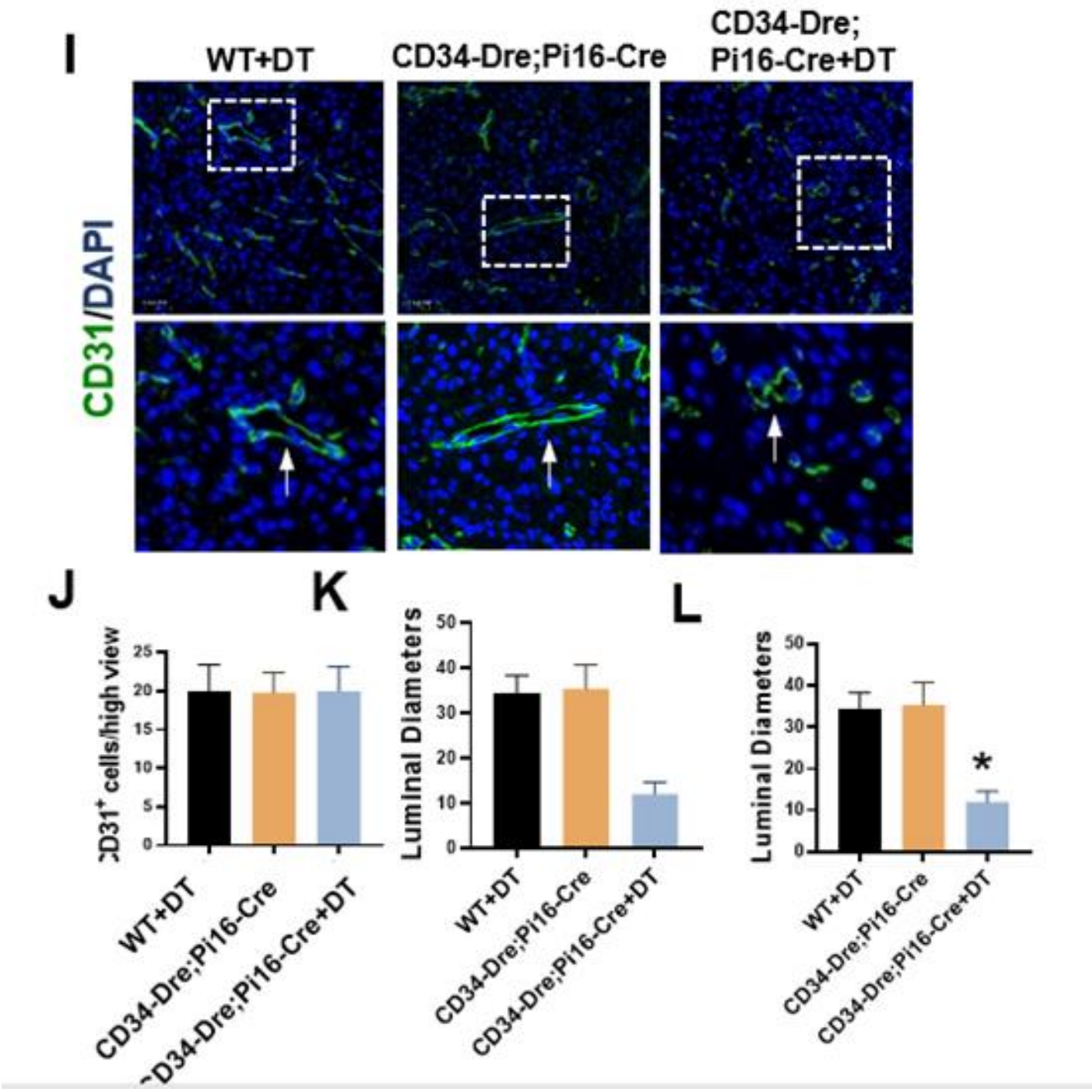

Referee 3 (Remarks for Author):

This revised version of the manuscript more accurately reflects the interpretation presented by Yang et al. The added analysis of human scRNA-seq data, together with the use of the dual Cre-Dre mouse model for lineage-specific ablation, substantially strengthens the evidence that this Pi16+ Cd34+ mesenchymal stem cell population contributes to the emergence of pro-tumorigenic Acta2^{+} CAFs specifically myCAF/ECM-producing CAFs and suggests that targeting their transformation may be therapeutically beneficial. The comprehensive set of in vitro and in vivo experiments, incorporating a genetic mouse model and multiple drug candidates targeting Foxs1, as well as the demonstrated loss of Acta2^{+} CAFs and associated tumor reduction, collectively provide a compelling mechanistic framework for pathogenic CAF transformation driven by cancer-cell signaling and Foxs1 activation.

Overall, the manuscript now presents a more thorough investigation and more effectively rules out alternative explanations involving other CD34^{+} cell types within the TME. Using multiple complementary tools, the authors convincingly show that mesenchymal cells/CAFs underline the pro-tumorigenic mechanism described.

Response: We appreciate the positive comments from the reviewer and revised the manuscript according to the specific comments.

Minor comments

1. Figures 4C and 7E: Please ensure uniform spacing between image tiles to improve readability.

Response: As the reviewer's suggested, we have now adjusted the spacing between image tiles to be uniform.

2. Repeated measures analysis: The statistical method used for repeated-measures experiments (e.g., Figures 4H/I and 5C) is not specified, and some of the reported significant differences appear unlikely across the full time course. If only certain time points are significant, please indicate this by placing asterisks above the relevant points and specify the statistical test used.

Response: Thank you for the reviewers' suggestions. We have revised Figures 4H/I and 5C accordingly. The statistical method used was ANOVA, which has now been indicated. The specific statistical method was chosen according to the number of experimental groups and the replicates within each group. Comparisons between two groups with ≥5 experimental repeats per group were assessed with the t-test. Comparisons across multiple groups with ≥5 experimental repeats per group were assessed with one-way ANOVA followed by Bonferroni post hoc analysis for normally distributed data. Comparisons across multiple groups with 3 experimental repeats per group were assessed with the Kruskal–Wallis test, followed by Bonferroni post hoc analysis. We have now included the detailed description in the **General statistical analyses** section of the Methods. The replicate number for each experiment has been added in the figure legend.

3. Figure 5C: The color scheme for the drug treatments does not match that used in other related figures (5D/F). There also appears to be inconsistency in drug naming (e.g., Erdafitinib in 5C vs. Erdafitiram in 5D). Additionally, the growth curve for the Erdafitinib treatment does not align with the tumor weight shown for the Erdafitiram group. If these represent different experiments, please clarify in the text; if they represent the same experiment, please correct the inconsistencies.

Response: Thank you for your helpful comments. We have unified the colors across figures so that the same drug treatment is now represented by the same color. We have also clarified this issue, i.e. Figures 5B, 5C, and 5D depict the same experiment; when quantifying tumor volumes, the group labels were inadvertently mis-assigned. After checking the original records, we have accurately presented the data.

4. Figures 5C-D and Supplementary Figure 10B: These are the only instances where "Penfluride" appears, which I assume is meant to be penfluridol. Please correct if so.

Response: Thank you for pointing this out. "Penfluride" in Figure 10B was indeed a typographical error; we have corrected it to "penfluridol".

5. Figure 7F - please match the colors of the different drugs to previous charts

Response: Thank you for the reviewers' comments. We have now consistently used the same color to represent all drug-related results.

We are grateful to the editors and reviewers for their time and insightful comments, which have greatly improved our manuscript. We have addressed all the concerns raised and hope the revised version is now acceptable for publication in the journal.

Yours sincerely

Li Zhang, MD, PhD
Professor and Chair of Department of Cardiology

24th Apr 2026

Dear Prof. Zhang,

Thank you for submitting your revised study. The initial referees have evaluated your revised manuscript and as you will see below, they are satisfied with the revisions. We will therefore be able to accept your manuscript once the following editorial matters are addressed:

1/ Manuscript text:
- Please remove the highlighted text and only keep in track changes mode any new modification.
- Please provide institutional email addresses for all three corresponding authors. An ORCID identifier is also missing for Wei Lin.
- Rename "Materials and Methods" to "Methods":
o Mice: please provide origin, age, gender, housing and husbandry conditions. State details of authority granting ethics approval (IRB or equivalent committee(s), provide reference number for approval. Include a statement of compliance with ethical regulations.
o Cells: please indicate origin, and whether the cells were authenticated and tested for mycoplasma contamination.
o Human participants: please include the full statement confirming that the experiments conformed to the principles set out in the WMA Declaration of Helsinki and the Department of Health and Human Services Belmont Report. State details of authority granting ethics approval (IRB or equivalent committee(s), provide reference number for approval. If collected and within the bounds of privacy constraints report on age, sex and gender or ethnicity for all study participants.
o Antibodies: please provide dilutions / concentrations.
o Statistics: please include a statement statement about sample size, blinding, and exclusion/inclusion criteria.
- Rename "Data Availability Statement and Policy" to "Data Availability", move the section after the Methods, and provide a direct URL to access the deposited datasets. Please remove "The original western blot images are provided in the Supplementary File".
- Acknowledgements: please note that fundings 81672363, 82125005, 82100503, 81800364, 81930010 and 8187020, and Medical Engineering Cross Fund of Shanghai Jiao Tong University (YG2025QNB48) are missing in the submission system, please add.
- Please remove Author contributions from the manuscript text, and provide instead CRediT (Contributor Role Taxonomy) terms in the submission system. These replace a narrative author contribution section in the manuscript.
- Please rename "Conflicts of interest" to "Disclosure and competing interests statement".
- Correct the references format to alphabetical order, 10 author names listed before et al, and remove DOIs for published articles .

2/ Figures:
- Please provide individual data points where possible.
- Main and EV figures should be uploaded individually.
- The resolution of the submitted figures is too low for blot and microscopy images. This reduction in resolution is commonly caused by converting original 16-bit TIFF files to RGB format for publication. While this is not inherently problematic, it can raise concerns about image integrity for critical readers. To avoid any misunderstanding and to meet EMBO Press standards, we kindly ask that you resubmit the complete figure set at the captured original data resolution.
- During our standard figure check, we noted potential anomaly in Appendix Fig S1, panels B and D. Please carefully check the composition of the figure, correct if needed, and clarify.
- Figure 1M: quantification fo CD34 and Pi16 are identical in both graphs, please clarify.
- As you have only 3 EV figures, we would encourage you to make some of your Appendix figures as EV figures. The Appendix should contain a table of content with page numbers, and the yellow highlights should be removed.
- Please move the table with primers to after the main figure legends, label it Table 1 and add a legend.
- Please add the heading "Expanded View Figure Legends" above the list of EV figure legends.
- Please address the queries from our data editors in the figure legends:
1. Please note that the exact p values are not provided in the legends of figures 1K, L, M; 2K, 3E, F, H, L; 4A, B, C, E, F, H, I, J, K, M; 5C, D, F, H; 6B, C, E; EV1 B, C, E; EV2 A-C.
2. Please note that information related to n is missing in the legends of figures 1C, F, H; EV1 E, EV3 E.
3. Please note that the error bars are not defined in the legend of figure EV1 E
4. Please note that the scale bar needs to be defined for figure 2D
5. Please note that scale bar and its definition are missing for figures 2J, 3I
6. Please note that the dotted borders are not defined in the legend of figure 2J. This needs to be rectified.
7. Please note that the white arrows are not defined in the legend of figure 1J, 2J, 3G, I. This needs to be rectified.

3/ Author checklist:
- Please fill in:
1. Cell materials/ authentication and mycoplasma contamination

2. Experimental study design and statistics/ inclusion exclusion criteria and statistical tests.
3. Ethics: Helsinki declaration
- Please clarify: Clinical trial reporting/phase II and III
- Please also fill the right column for all completed sections

4/ Synopsis:
Please remove the synopsis text from the manuscript file and upload it separately. Please correct the formatting to include a short stand first (maximum of 300 characters, including space) as well as 2-5 one-sentences bullet points that summarize the paper (maximum of 30 words / bullet point). Please write the bullet points to summarize the key NEW findings. They should be designed to be complementary to the abstract - i.e. not repeat the same text.

The summary figure should be uploaded without the label as a synopsis image and in jpg or png format, with the dimensions 550 pixels wide x 300 pixel high. The text should remain legible.

5/ As part of the EMBO Publications transparent editorial process initiative (see our Editorial at http://embomolmed.embopress.org/content/2/9/329), EMBO Molecular Medicine will publish online a Review Process File (RPF) to accompany accepted manuscripts.
This file will be published in conjunction with your paper and will include the anonymous referee reports, your point-by-point response and all pertinent correspondence relating to the manuscript. Let us know whether you agree with the publication of the RPF and as here, if you want to remove or not any figures from it prior to publication.
Please note that the Authors checklist will be published at the end of the RPF.

I look forward to receiving your revised manuscript.

Yours sincerely,

Lise Roth

Lise Roth, PhD
Senior Editor
EMBO Molecular Medicine

***** Reviewer's comments *****

Referee #1 (Comments on Novelty/Model System for Author):

No further comments

Referee #2 (Remarks for Author):

The authors have adequately addressed my concerns and should be suitable for publication.

Referee #3 (Comments on Novelty/Model System for Author):

THe methods are adequate and while novelty is not extremely high it is appropriate for publication

Referee #3 (Remarks for Author):

The authors addressed my comments

The authors addressed the remaining editorial issues.

15th Jun 2026

Dear Prof. Zhang,

Thank you for submitting your revised files. We are pleased to inform you that your manuscript is accepted for publication and is now being sent to our publisher to be included in the next available issue of EMBO Molecular Medicine.

Please note that the deposited datasets are not yet publicly accessible (see attached screenshot). Could you please update the access settings and let us know once this has been done?

Your manuscript will be processed for publication by EMBO Press. It will be copy edited and you will receive page proofs prior to publication. Please note that you will be contacted by Springer Nature Author Services to complete licensing and payment information.

You may qualify for financial assistance for your publication charges - either via a Springer Nature fully open access agreement or an EMBO initiative. Check your eligibility: https://link.springer.com/journal/44321/how-to-publish-with-us

Should you be planning a Press Release on your article, please notify the production team via this form in order to coordinate publication and release dates: https://forms.cloud.microsoft/e/eH4A8JVP6g

If you have any questions, please do not hesitate to contact the Editorial Office. Thank you for your contribution to EMBO Molecular Medicine.

Yours sincerely,

Lise Roth

Lise Roth, Ph.D
Senior Editor
EMBO Molecular Medicine

>>> Please note that it is EMBO Molecular Medicine policy for the transcript of the editorial process (containing referee reports and your response letter) to be published as an online supplement to each paper. If you do NOT want this, you will need to inform the Editorial Office via email immediately. More information is available here: https://link.springer.com/partners/embo-press/editorial-policies#Peer%20review

EMBO Press Author Checklist

Corresponding Author Name: Li Zhang	
Journal Submitted to: EMBO molecular medicine	
Manuscript Number: EMM-2024-21040-V4	

USEFUL LINKS FOR COMPLETING THIS FORM
The EMBO Journal - Author Guidelines
EMBO Reports - Author Guidelines
Molecular Systems Biology - Author Guidelines
EMBO Molecular Medicine - Author Guidelines

Reporting Checklist for Life Science Articles (updated January
This checklist is adapted from Materials Design Analysis Reporting (MDAR) Checklist for Authors. MDAR establishes a minimum set of requirements in transparent reporting in the life sciences (see Statement of Task: 10.31222/osf.io/9sm4x). Please follow the journal's guidelines in preparing your
Please note that a copy of this checklist will be published alongside your article.

Abridged guidelines for figures
1. Data
The data shown in figures should satisfy the following conditions:
- the data were obtained and processed according to the field's best practice and are presented to reflect the results of the experiments in an accurate and unbiased manner.
- ideally, figure panels should include only measurements that are directly comparable to each other and obtained with the same assay.
- plots include clearly labeled error bars for independent experiments and sample sizes. Unless justified, error bars should not be shown for technical
- if n<5, the individual data points from each experiment should be plotted. Any statistical test employed should be justified.
- Source Data should be included to report the data underlying figures according to the guidelines set out in the authorship guidelines on Data

2. Captions
Each figure caption should contain the following information, for each panel where they are relevant:
- a specification of the experimental system investigated (eg cell line, species name).
- the assay(s) and method(s) used to carry out the reported observations and measurements.
- an explicit mention of the biological and chemical entity(ies) that are being measured.
- an explicit mention of the biological and chemical entity(ies) that are altered/varied/perturbed in a controlled manner.
- the exact sample size (n) for each experimental group/condition, given as a number, not a range;
- a description of the sample collection allowing the reader to understand whether the samples represent technical or biological replicates (including how many animals, litters, cultures, etc.).
- a statement of how many times the experiment shown was independently replicated in the laboratory.
- definitions of statistical methods and measures:
  - common tests, such as t-test (please specify whether paired vs. unpaired), simple χ2 tests, Wilcoxon and Mann-Whitney tests, can be unambiguously identified by name only, but more complex techniques should be described in the methods section;
  - are tests one-sided or two-sided?
  - are there adjustments for multiple comparisons?
  - exact statistical test results, e.g., P values = x but not P values < x;
  - definition of 'center values' as median or average;
  - definition of error bars as s.d. or s.e.m.

Please complete ALL of the questions below.
Select "Not Applicable" only when the requested information is not relevant for your study.

Materials

Newly Created Materials	Information included in the manuscript?	In which section is the information available? (Reagents and Tools Table, Materials and Methods, Figures, Data Availability Section)
New materials and reagents need to be available; do any restrictions apply?	Yes	Materials and Methods

Antibodies	Information included in the manuscript?	In which section is the information available? (Reagents and Tools Table, Materials and Methods, Figures, Data Availability Section)
For **antibodies** provide the following information:<br>- Commercial antibodies: RRID (if possible) or supplier name, catalogue number and or/clone number<br>- Non-commercial: RRID or citation	Yes	Reagents and Tools Table; Materials and Methods

DNA and RNA sequences	Information included in the manuscript?	In which section is the information available? (Reagents and Tools Table, Materials and Methods, Figures, Data Availability Section)
**Short novel DNA or RNA including primers, probes**: provide the sequences.	Yes	Materials and Methods

Cell materials	Information included in the manuscript?	In which section is the information available? (Reagents and Tools Table, Materials and Methods, Figures, Data Availability Section)
**Cell lines:** Provide species information, strain. Provide accession number in repository **OR** supplier name, catalog number, clone number, and/**OR** RRID.	Yes	Reagents and Tools Table; Materials and Methods
**Primary cultures:** Provide species, strain, sex of origin, genetic modification status.	Yes	Materials and Methods
Report if the cell lines were recently **authenticated** (e.g., by STR profiling) and tested for mycoplasma contamination.	Yes	Reagents and Tools Table; Materials and Methods

Experimental animals	Information included in the manuscript?	In which section is the information available? (Reagents and Tools Table, Materials and Methods, Figures, Data Availability Section)
**Laboratory animals or Model organisms:** Provide species, strain, sex, age, genetic modification status. Provide accession number in repository **OR** supplier name, catalog number, clone number, **OR** RRID.	Yes	Materials and Methods
**Animal observed in or captured from the field:** Provide species, sex, and age where possible.	Not Applicable	Not Applicable
Please detail **housing and husbandry conditions**.	Yes	Materials and Methods

Plants and microbes	Information included in the manuscript?	In which section is the information available? (Reagents and Tools Table, Materials and Methods, Figures, Data Availability Section)
**Plants:** provide species and strain, ecotype and cultivar where relevant, unique accession number if available, and source (including location for collected wild specimens).	Not Applicable	Not Applicable
**Microbes:** provide species and strain, unique accession number if available, and source.	Not Applicable	Not Applicable

Human research participants	Information included in the manuscript?	In which section is the information available? (Reagents and Tools Table, Materials and Methods, Figures, Data Availability Section)
If collected and within the bounds of privacy constraints report on age, sex and gender or ethnicity for all study participants.	Yes	Materials and Methods

Core facilities	Information included in the manuscript?	In which section is the information available? (Reagents and Tools Table, Materials and Methods, Figures, Data Availability Section)
If your work benefited from core facilities, was their service mentioned in the acknowledgments section?	Not Applicable	Not Applicable

Design

Study protocol	Information included in the manuscript?	In which section is the information available? (Reagents and Tools Table, Materials and Methods, Figures, Data Availability Section)
If study protocol has been **pre-registered, provide DOI in the manuscript**. For clinical trials, provide the trial registration number **OR** cite DOI.	Not Applicable	Not Applicable
Report the **clinical trial registration number** (at ClinicalTrials.gov or equivalent), where applicable.	Not Applicable	Not Applicable

Laboratory protocol	Information included in the manuscript?	In which section is the information available? (Reagents and Tools Table, Materials and Methods, Figures, Data Availability Section)
Provide DOI OR other citation details if **external detailed step-by-step protocols** are available.	Yes	Materials and Methods

Experimental study design and statistics	Information included in the manuscript?	In which section is the information available? (Reagents and Tools Table, Materials and Methods, Figures, Data Availability Section)
Include a statement about **sample size** estimate even if no statistical methods were used.	Yes	Materials and Methods, Figures
Were any steps taken to minimize the effects of subjective bias when allocating animals/samples to treatment (e.g. **randomization procedure**)? If yes, have they been described?	Yes	Materials and Methods
Include a statement about **blinding** even if no blinding was done.	Yes	Materials and Methods
Describe **inclusion/exclusion criteria** if samples or animals were excluded from the analysis. Were the criteria pre-established?<br><br>If sample or data points were omitted from analysis, report if this was due to attrition or intentional exclusion and provide justification.	Yes	Materials and Methods
For every figure, are **statistical tests** justified as appropriate? Do the data meet the assumptions of the tests (e.g., normal distribution)? Describe any methods used to assess it. Is there an estimate of variation within each group of data? Is the variance similar between the groups that are being statistically compared?	Yes	Materials and Methods, Figure legends

Sample definition and in-laboratory replication	Information included in the manuscript?	In which section is the information available? (Reagents and Tools Table, Materials and Methods, Figures, Data Availability Section)
In the figure legends: state number of times the experiment was **replicated** in laboratory.	Yes	Figure legends
In the figure legends: define whether data describe **technical or biological replicates**.	Yes	Figure legends

Ethics

Ethics	Information included in the manuscript?	In which section is the information available? (Reagents and Tools Table, Materials and Methods, Figures, Data Availability Section)
Studies involving **human participants**: State details of **authority granting ethics approval** (IRB or equivalent committee(s), provide reference number for approval.	Yes	Materials and Methods
Studies involving **human participants**: Include a statement confirming that **informed consent** was obtained from all subjects and that the experiments conformed to the principles set out in the WMA Declaration of Helsinki and the Department of Health and Human Services Belmont Report.	Yes	Materials and Methods
Studies involving **human participants: For publication of patient photos**, include a statement confirming that consent to publish was obtained.	Not Applicable	Not Applicable
Studies involving experimental **animals**: State details of **authority granting ethics approval** (IRB or equivalent committee(s), provide reference number for approval. Include a statement of compliance with ethical regulations.	Yes	Materials and Methods
Studies involving **specimen and field samples:** State if relevant **permits** obtained, provide details of authority approving study; if none were required, explain why.	Not Applicable	Not Applicable

Dual Use Research of Concern (DURC)	Information included in the manuscript?	In which section is the information available? (Reagents and Tools Table, Materials and Methods, Figures, Data Availability Section)
Could your study fall under dual use research restrictions? Please check biosecurity documents and list of **select agents and toxins** (CDC): https://www.selectagents.gov/sat/list.htm	Not Applicable	Not Applicable
If you used a select agent, is the security level of the lab appropriate and reported in the manuscript?	Not Applicable	Not Applicable
If a study is subject to dual use research of concern regulations, is the name of the **authority granting approval and reference number** for the regulatory approval provided in the manuscript?	Not Applicable	Not Applicable

Reporting

The MDAR framework recommends adoption of discipline-specific guidelines, established and endorsed through community initiatives. Journals have their own policy about requiring specific guidelines and recommendations to complement MDAR.

Adherence to community standards	Information included in the manuscript?	In which section is the information available? (Reagents and Tools Table, Materials and Methods, Figures, Data Availability Section)
State if relevant guidelines or checklists (e.g., **ICMJE, MIBBI, ARRIVE, PRISMA**) have been followed or provided.	Not Applicable	Not Applicable
For **tumor marker prognostic studies,** we recommend that you follow the **REMARK** reporting guidelines (see link list at top right). See author guidelines, under 'Reporting Guidelines'. Please confirm you have followed these guidelines.	Not Applicable	Not Applicable
For **phase II and III randomized controlled trials**, please refer to the **CONSORT** flow diagram (see link list at top right) and submit the CONSORT checklist (see link list at top right) with your submission. See author guidelines, under 'Reporting Guidelines'. Please confirm you have submitted this list.	Not Applicable	Not Applicable

Data Availability

Data availability	Information included in the manuscript?	In which section is the information available? (Reagents and Tools Table, Materials and Methods, Figures, Data Availability Section)
Have **primary datasets** been deposited according to the journal's guidelines (see 'Data Deposition' section) and the respective accession numbers provided in the Data Availability Section?	Yes	Data Availability Section
Were **human clinical and genomic datasets** deposited in a public access-controlled repository in accordance to ethical obligations to the patients and to the applicable consent agreement?	Not Applicable	Not Applicable
Are **computational models** that are central and integral to a study available without restrictions in a machine-readable form? Were the relevant accession numbers or links provided?	Not Applicable	Not Applicable
If publicly available data were reused, provide the respective **data citations in the reference list**.	Yes	Materials and Methods; References
