# Supplementary material for: Foxs1-mediated transformation of CD34+ fibroblast to myCAFs promotes tumor growth
Source: EMBO Mol Med. 2026 Jul 4;18(8):3200–25. doi: 10.1038/s44321-026-00476-8 (PMC13469093; doi:10.1038/s44321-026-00476-8)
Supplement: Supplementary file 3 [file 44321_2026_476_MOESM9_ESM.pdf]

Expanded View Figures

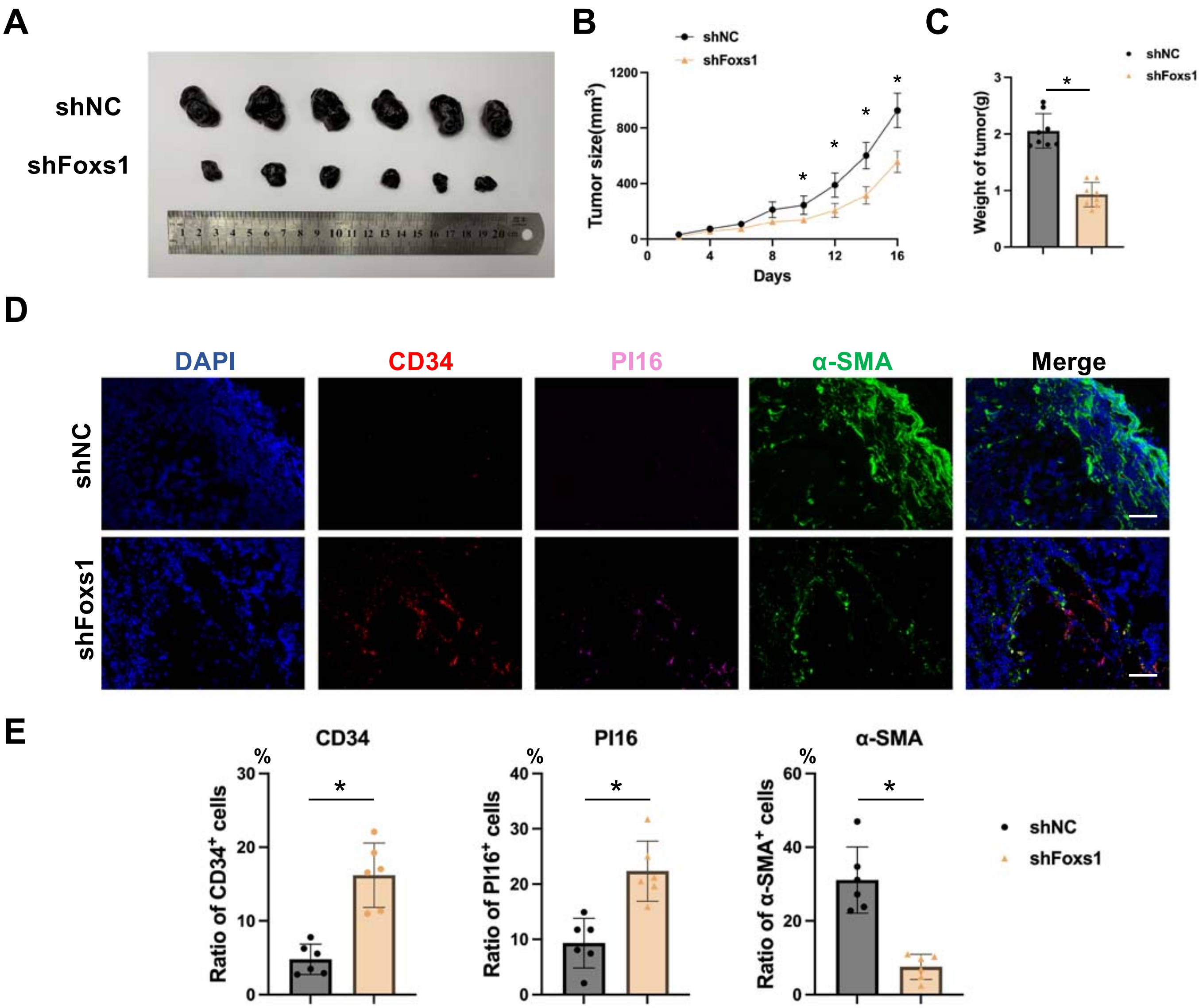

Figure EV1. Foxs1 knockdown in fibroblasts inhibits tumor growth.

(**A**) Photographs of excised tumors from shNC and shFoxs1 mice. (**B**) Tumor volume over time. Data represent mean ± SD; $n = 8$. Statistical significance was assessed at each time point using an unpaired two-tailed Student's *t* test. Significant exact *P* values were: Day 10: shNC vs. shFoxs1, $P = 0.0008$; Day 12: shNC vs. shFoxs1, $P = 1.49 \times 10^{-4}$; Day 14: shNC vs. shFoxs1, $P = 5.66 \times 10^{-6}$; Day 16: shNC vs. shFoxs1, $P = 5.13 \times 10^{-6}$. $^*P < 0.05$. (**C**) Quantification of tumor weight. Data represent mean ± SD; $n = 8$. Statistical significance was assessed using an unpaired two-tailed Student's *t* test; shNC vs. shFoxs1, $P = 6.82 \times 10^{-7}$; $^*P < 0.05$. (**D, E**) IF staining for CD34, PI16, and α-SMA/COL1A1 in tumor tissues from the two mouse groups (**D**), with quantification (**E**). Scale Bars:100 µm. Data are mean ± SD; $n = 5$. Statistical significance was assessed using an unpaired two-tailed Student's *t* test; Significant exact *P* values were: CD34, shNC vs. shFoxs1, $P = 0.0002$; PI16, shNC vs. shFoxs1, $P = 0.0011$; α-SMA, shNC vs. shFoxs1, $P = 0.0001$; $^*P < 0.05$.

© The Author(s)

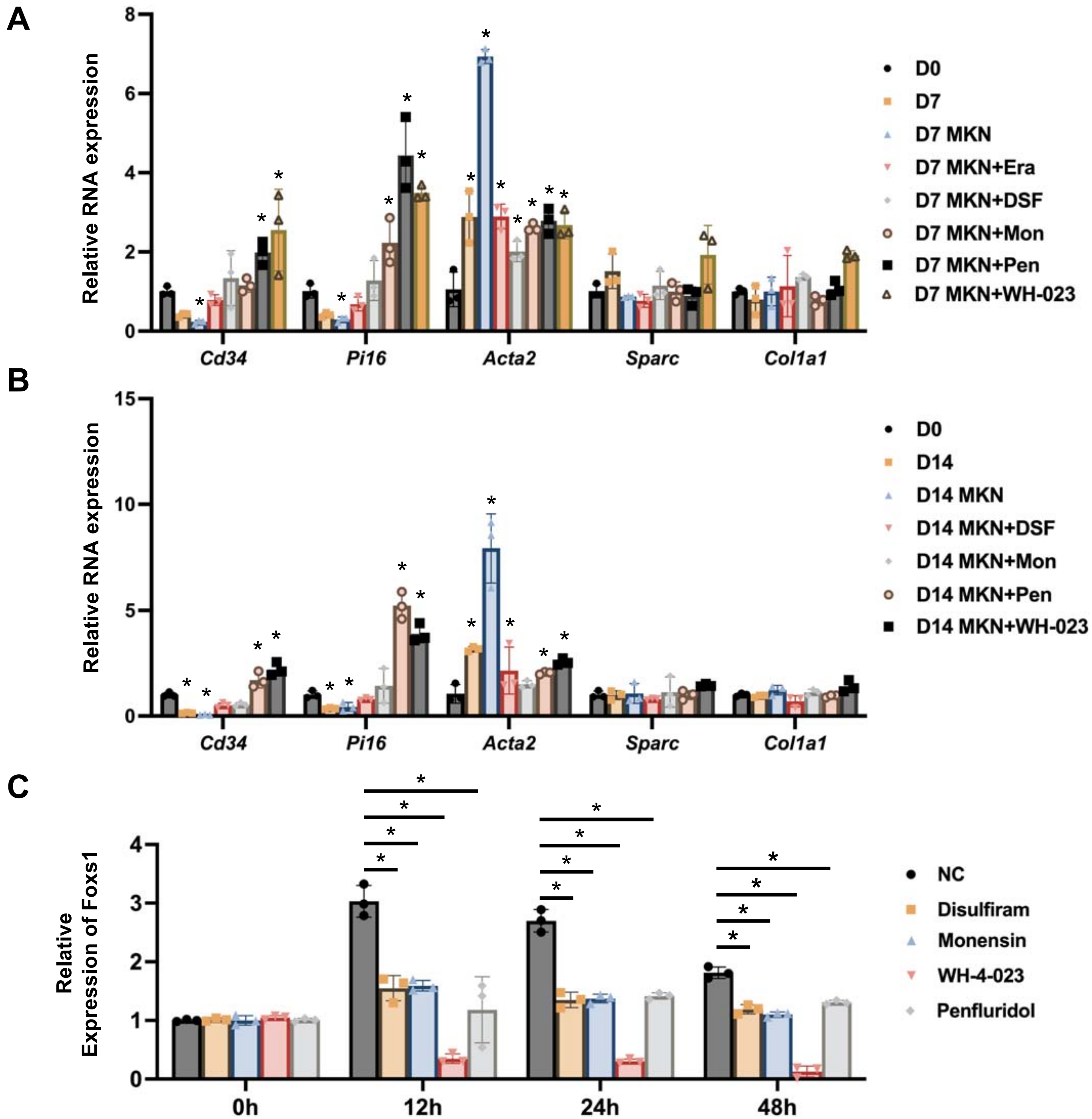
A
Relative RNA expression
D0
D7
D7 MKN
D7 MKN+Era
D7 MKN+DSF
D7 MKN+Mon
D7 MKN+Pen
D7 MKN+WH-023
Cd34
Pi16
Acta2
Sparc
Col1a1
B
Relative RNA expression
D0
D14
D14 MKN
D14 MKN+DSF
D14 MKN+Mon
D14 MKN+Pen
D14 MKN+WH-023
C
Relative Expression of Foxs1
NC
Disulfiram
Monensin
WH-4-023
Penfluridol
0h
12h
24h
48h

 © The Author(s)

Figure EV2. Drug candidates inhibit Foxs1 expression.

(**A**, **B**) qPCR analysis of *Cd34*, *Pi16*, *Acta2*, *Sparc*, and *Col1a1* expression in *Cd34⁺Pl16⁺* CAFs cultured under the indicated conditions for 7 days (**A**) or 14 days (**B**). Data represent mean ± SD; $n = 3$ independent experiments. Statistical significance was assessed using two-way ANOVA followed by Šídák's multiple comparisons test comparing each condition with the D0 group within each gene. Significant Šídák-adjusted *P* values were: (**A**) *Cd34*: D7 MKN, $P = 0.0011$; D7 MKN+Pen, $P = 0.0199$; D7 MKN + WH-023, $P = 0.0023$. *Pi16*: D7 MKN, $P = 0.0024$; D7 MKN+Mon, $P = 8.97 \times 10^{-5}$; D7 MKN+Pen, $P = 1.75 \times 10^{-9}$; D7 MKN + WH-023, $P = 7.84 \times 10^{-6}$. *Acta2*: D7, $P = 4.45 \times 10^{-6}$; D7 MKN, $P = 1.52 \times 10^{-18}$; D7 MKN+Era, $P = 4.65 \times 10^{-6}$; D7 MKN + DSF, $P = 0.015$; D7 MKN+Mon, $P = 3.25 \times 10^{-6}$; D7 MKN+Pen, $P = 6.35 \times 10^{-5}$; D7 MKN + WH-023, $P = 0.0014$. (**B**) *Cd34*: D14, $P = 6.43 \times 10^{-5}$; D14 MKN, $P = 0.0005$; D14 MKN+Pen, $P = 0.0434$; D14 MKN + WH-023, $P = 7.25 \times 10^{-5}$. *Pi16*: D14, $P = 0.0016$; D14 MKN, $P = 0.0213$; D14 MKN+Pen, $P = 7.41 \times 10^{-13}$; D14 MKN + WH-023, $P = 6.29 \times 10^{-11}$. *Acta2*: D14, $P = 3.53 \times 10^{-11}$; D14 MKN, $P = 0.0032$; D14 MKN + DSF, $P = 0.0081$; D14 MKN+Pen, $P = 0.003$; D14 MKN + WH-023, $P = 3.27 \times 10^{-6}$. $^*P < 0.05$. (**C**) qPCR profiling of Foxs1 in drug candidate-treated $Cd34^{+}Pi16^{+}$ cells for 0,12, 24, 48 h. Data are mean ± SD; $n = 3$ independent experiments. Statistical significance was assessed at each time point using an unpaired two-tailed Student's *t* test against the NC group. Significant exact *P* values were: 12 h: NC vs. Disulfiram, $P = 0.0018$; NC vs. Monensin, $P = 0.0010$; NC vs. WH-4-023, $P = 7.98 \times 10^{-5}$; NC vs. Penfluridol, $P = 0.0069$. 24 h: NC vs. Disulfiram, $P = 0.0006$; NC vs. Monensin, $P = 0.0004$; NC vs. WH-4-023, $P = 2.91 \times 10^{-5}$; NC vs. Penfluridol, $P = 0.0004$. 48 h: NC vs. Disulfiram, $P = 0.001$; NC vs. Monensin, $P = 0.0003$; NC vs. WH-4-023, $P = 2.80 \times 10^{-5}$; NC vs. Penfluridol, $P = 0.0011$. $^*P < 0.05$.

© The Author(s)

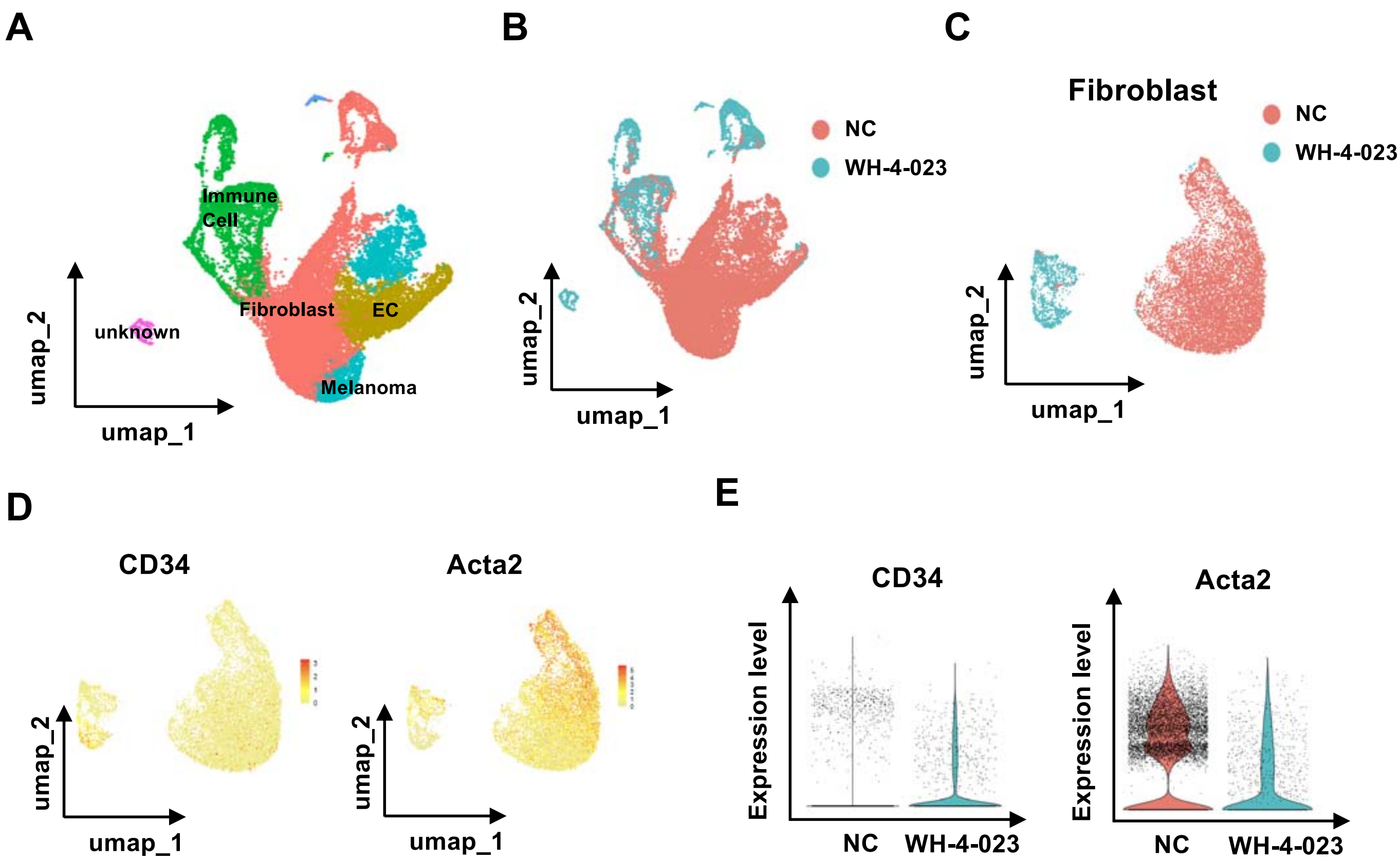

Figure EV3. WH-4-023 inhibits the transition of CD34$^+$ fibroblasts into Acta2+CAFs.

(**A**) UMAP plot of the color-coded major cell types of Tomato$^+$ cells encompassing *Cd34*$^+$ cell-derived TME of *Cd34*-CreERT2; Rosa26-LSL-tdTomato mice. (**B**) UMAP plot of Tomato$^+$ cells by samples. (**C**) UMAP plots of total fibroblasts of Cd34-CreERT2/Rosa26-LSL-tdTomato mice melanoma samples. (**D**) UMAP plots of CD34 and Acta2 of fibroblast in UMAP plots. (**E**) and Violin plots of CD34 and Acta2 expression by samples. Statistical significance was assessed using two-tailed Student's *t* test. $n = 6$ (10,752 cells in total).NC: 9460 cells; WH-4-023: 1292 cells.

 © The Author(s)
